# Inhibition of Aurora‐A/N‐Myc Protein–Protein Interaction Using Peptidomimetics: Understanding the Role of Peptide Cyclization**

**DOI:** 10.1002/cbic.202300649

**Published:** 2023-11-27

**Authors:** Robert S. Dawber, Diana Gimenez, Matthew Batchelor, Jennifer A. Miles, Megan H. Wright, Richard Bayliss, Andrew J. Wilson

**Affiliations:** ^1^ Astbury Centre for Structural Molecular Biology University of Leeds Woodhouse Lane Leeds LS2 9JT UK; ^2^ School of Chemistry University of Leeds Woodhouse Lane Leeds LS2 9JT UK; ^3^ School of Molecular and Cellular Biology University of Leeds Woodhouse Lane Leeds LS2 9JT UK; ^4^ School of Chemistry University of Birmingham Edgbaston, Birmingham B15 2TT UK

**Keywords:** Aurora-A kinase, constrained peptides, N-Myc, protein-protein interactions

## Abstract

Using N‐Myc_61‐89_ as a starting template we showcase the systematic use of truncation and maleimide constraining to develop peptidomimetic inhibitors of the N‐Myc/Aurora‐A protein–protein interaction (PPI); a potential anticancer drug discovery target. The most promising of these – N‐Myc_73‐94‐N85C/G89C‐**mal**_ – is shown to favour a more Aurora‐A compliant binding ensemble in comparison to the linear wild‐type sequence as observed through fluorescence anisotropy competition assays, circular dichroism (CD) and nuclear magnetic resonance (NMR) experiments. Further *in silico* investigation of this peptide in its Aurora‐A bound state, by molecular dynamics (MD) simulations, imply (i) the bound conformation is more stable as a consequence of the constraint, which likely suppresses dissociation and (ii) the constraint may make further stabilizing interactions with the Aurora‐A surface. Taken together this work unveils the first orthosteric N‐Myc/Aurora‐A inhibitor and provides useful insights on the biophysical properties and thus design of constrained peptides, an attractive therapeutic modality.

## Graphical abstract

A maleimide constraint predisposes an N‐Myc derived peptidomimetic towards Aurora‐A recognition by restricting the accessible conformational landscape of the unbound peptide, promoting a more stable helical bound state and making additional non‐covalent contacts with Aurora‐A.

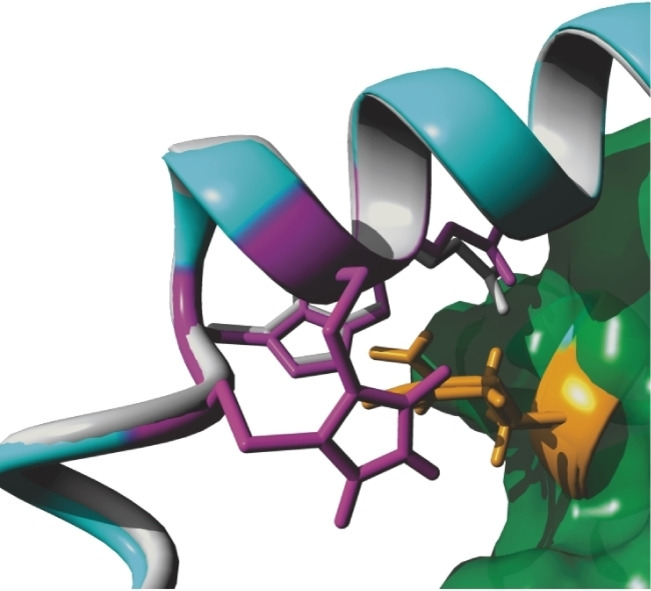

## Introduction

**Figure 1:**
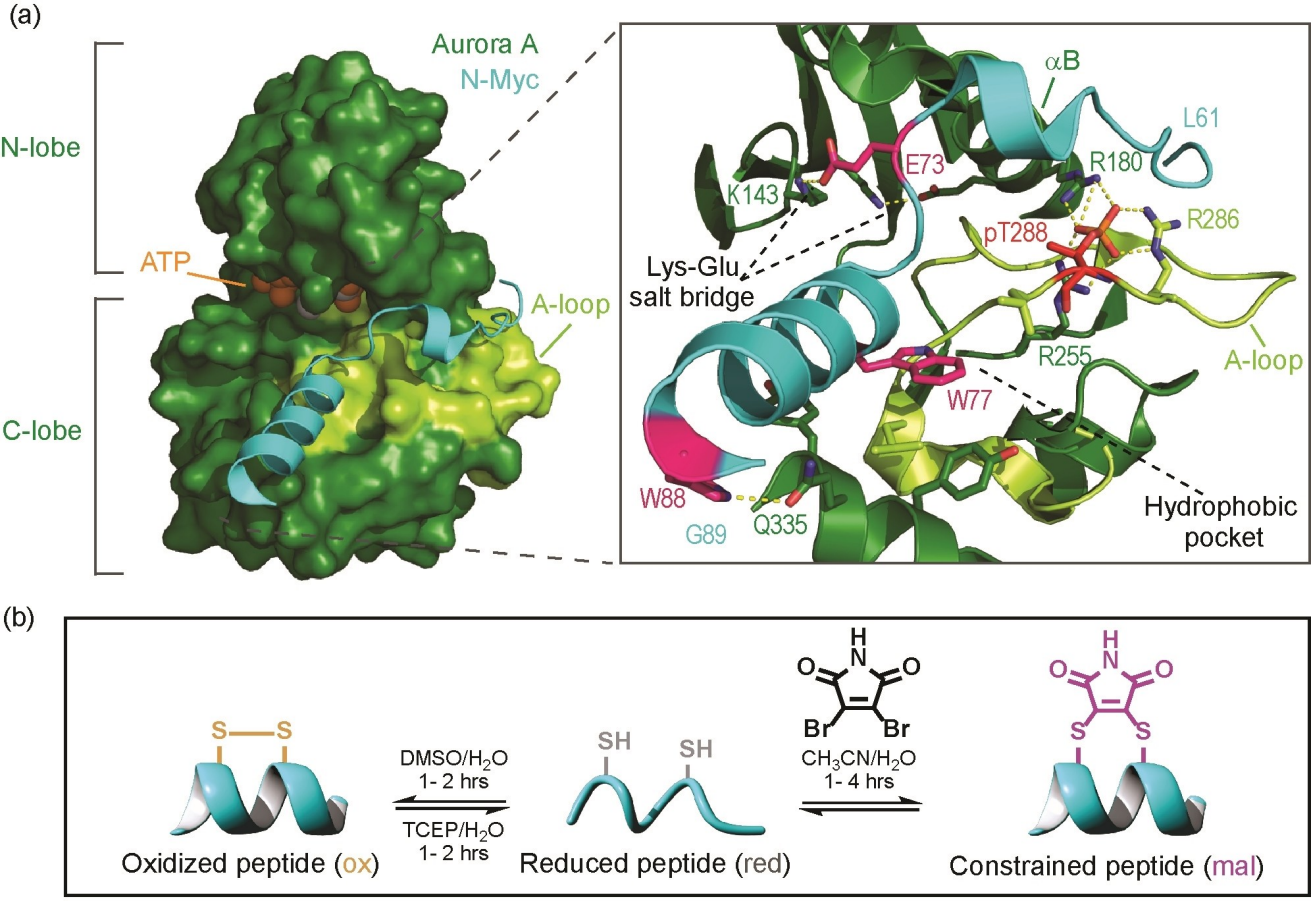
**Key features of the Aurora‐A/N** *‐* **Myc complex**. (a) Crystal structure of the Aurora‐A_C290A/C393A_ catalytic domain (forest‐green) in complex with N*‐*Myc_61‐89_ (cyan; PDB: 5G1X). Magnified view of the Aurora‐A/N*‐*Myc PPI interface with A‐loop and P+1 pocket shown (limon). Key residues shown as sticks, with pThr288^AurA^ residue (red) and hot‐spot residues (deep‐pink) highlighted. Charge‐reinforced or polar contacts between side chains are shown by dashed yellow lines. (b) Scheme illustrating the use of reversible chemistry for the synthesis of maleimide constrained peptides.

The Aurora kinases play essential roles in mitosis and have received attention as targets for drug‐development.^[1]^ Aurora‐A represents a promising target for development of anticancer therapeutics; however whilst potent and selective active site inhibitors have been identified, these have yet to be approved for therapeutic use. Such compounds might be expected to have a narrow therapeutic window because Aurora‐A has many roles, including a critical function in bipolar mitotic spindle assembly.[^1^, ^2^] Aurora‐A is an incomplete kinase; its localization and activation are regulated through protein–protein interactions (PPIs). Targeting these PPI interfaces may represent an alternative approach to ATP‐competitive inhibitors. One candidate PPI is the N‐Myc/Aurora‐A interaction.^[3]^ *MYCN* (the gene that encodes for the N‐Myc protein) was discovered through its association with neuroblastoma, a cancer of the nervous system that affects children.[^4^, ^5^] Elevated levels of N‐Myc have also been observed in several other cancers.^[6]^ In 2016, Richards *et al*. published an X‐ray co‐crystal structure of Aurora‐A in complex with an intrinsically disordered region of N‐Myc (residues 28–89);^[7]^ although residues 28–60 were not observed in the structure, residues 61–89 of N‐Myc were found to undergo a disorder to order transition to interact in an α‐helical conformation with the cleft between the Aurora‐A N‐ and C‐lobes formed by the αB‐/αC‐helices, the A‐loop, and the αG‐helix (Figure 1a). The formation of this complex has been proposed to explain why Aurora‐A stabilizes N‐Myc against degradation, amplifying the effects of N‐Myc overexpression.

Certain ATP‐competitive Aurora‐A inhibitors have been shown to perturb the N‐Myc/Aurora‐A interaction due to inhibitor‐induced conformational changes in Aurora‐A that disrupt the PPI interface; competition co‐precipitation experiments found that MLN8054, but not CCT137690, disrupted the N‐Myc/Aurora‐A interaction.^[7]^ Similarly, Weiss and co‐workers developed a series of conformation‐disrupting Aurora‐A inhibitors based on diaminopyrimidine and pyrazolopyrimidine scaffolds, such as CD532.^[8]^ Although CD532 was shown to be a potent N‐Myc/Aurora‐A inhibitor that reduced N‐Myc protein levels in xenographs, concomitant inhibition of kinase activity might still perturb other cellular essential functions.^[9]^ An alternative approach has been to use Aurora‐A degraders/PROTACS:^[10]^ The first‐in‐class Aurora‐A/N‐Myc degrader, HLB‐0532259 was developed from a novel Aurora‐A‐binding ligand that engages the N‐Myc/Aurora‐A complex. HLB‐0532259 was shown to promote degradation of Aurora‐A and N‐Myc with superior potency and excellent selectivity in comparison to established allosteric Aurora‐A inhibitors.^[11]^ Despite these advances, it would be desirable to have orthosteric inhibitors of N‐Myc/Aurora‐A interaction available as tools to dissect out the mechanistic role of this interaction and to serve as alternative leads for drug discovery.

The larger PPI surface and the N‐Myc helical epitope make a peptidomimetic approach attractive. Peptides have emerged as promising alternatives to small molecules given the advantages they present in terms of potent and selective target affinity; however, they suffer from poor (proteolytic) stability and suboptimal cell permeability, motivating the development of constrained peptides.^[12]^ Here we describe the first competitive inhibitor of the N‐Myc/Aurora‐A interaction. Using a peptidomimetic approach we show that a truncated and constrained peptide has enhanced inhibitory potency in biophysical experiments when compared to its unconstrained analogue. This represents a starting point for the development of reagents that could be used to explore inhibition of N‐Myc/Aurora‐A interaction for cancer chemotherapy. Moreover, using fluorescence anisotropy, circular dichroism, NMR structural analyses of the unbound peptides and molecular dynamics simulations for the Aurora‐A bound peptides, we show that introduction of the constraint confers enhanced potency through a combination of subtle effects. Whilst the constraint does not pre‐organize the peptide in an α‐helical conformation, it likely: (i) restricts the accessible conformational landscape of the peptide, raising it in energy and pre‐disposing the unbound peptide to Aurora‐A recognition; (ii) stabilizes the bound conformation to suppress dissociation; (iii) introduces stabilizing contacts between constraint and protein.

## Results and Discussion

### N‐Myc_61‐89_ peptide truncation and sequence variation

Fluorescence anisotropy titrations previously established that N‐Myc_61–89_ binds to Aurora‐A with low μM affinity.^[7]^ Using either Trp>Ala or Glu>Lys variants, three residues in N‐Myc_61‐89_ were found to have a strong effect on binding affinity (PDB: 5G1X; Figure 1a): tryptophan residues Trp77^N‐Myc^, Trp88^N‐Myc^ and Glu73^N‐Myc^, which presumably participates in a salt bridge with Aurora‐A via Lys143^AurA^. Based on prior studies Leu61^N‐Myc^ was considered to make an energetic contribution to binding, however, despite a number of non‐covalent contacts (e. g. π–π stacking between Phe67^N‐Myc^ and His176^AurA^, hydrogen bonds between Ser64^N‐Myc^ and His280^AurA^, Ser64^N‐Myc^ and Arg286^AurA^, Arg65^N‐Myc^ and Arg180^AurA^, Glu73^N‐Myc^ and Ala172^AurA^) we considered this region ripe for removal. Thus, we started these studies by probing the effects on binding of removing residues from the 61–75 region (Table 1, Figure S1). A short N*‐*Myc variant spanning the helical region (N*‐*Myc_73‐89_) was prepared and its IC_50_ value determined through competition against a fluorescein labelled N*‐*Myc_61‐89_ sequence (Table 1). Despite maintaining all three confirmed hot‐spot residues (Glu73^N‐Myc^, Trp77^N‐Myc,^ and Trp88^N‐Myc^), removal of residues 61–72 decreased the inhibitory potency of N*‐*Myc (N*‐*Myc_73‐89_ IC_50_=179±19 μM verses N*‐*Myc_61‐89_ IC_50_=42±4 μM). A slightly shorter variant (N*‐*Myc_76‐89_) also exhibited similarly diminished potency (IC_50_=215±11 μM). To further investigate the relevance of the Glu73^N‐Myc^/Lys143^AurA^ salt bridge for N*‐*Myc/Aurora‐A binding, additional variants with Glu73>phosphoserine (pSer) and Glu73>Ser substitutions were assessed (N*‐*Myc_73‐89‐E73pS_ and N*‐*Myc_73‐89‐E73S_; Table 1). The effects of both Glu73^N‐Myc^ substitutions on potency were subtle, suggesting that this side chain does not make a major contribution to binding. We then assessed the effects of sequence elongation at the C‐terminus, by preparing N*‐*Myc_73‐94_ and N‐Myc_73‐97_, with C‐terminal elongations of five and eight residues, respectively (Table 1). Overall, the results indicate that the five residues beyond Gly89^N‐Myc^ contribute little to binding, as only further elongation of the sequence increased potency (N*‐*Myc_73‐97_, IC_50_=79±4 μM).

**Table 1:** Effects of sequence truncation, elongation, and single point variations in N‐Myc_61‐89_.

Peptide	Sequence^[a]^	IC_50_^[b]^ (μM)	MRE_222_^[c]^ (deg cm^−1^ dmol^−1^ res^−1^)	% Helicity^[d]^
N*‐*Myc_61‐89_	Ac ‐LSPSRGFAEHSSEPPSWVTEMLLENELWG ‐NH_2_	42±4	−2059±64	6±1
N*‐*Myc_73‐89_	Ac‐ EPPSWVTEMLLENELWG ‐NH_2_	179±19	−4079±71	13±1
N*‐*Myc_76‐89_	Ac‐ SWVTEMLLENELWG ‐NH_2_	215±11	−4342±142	14±1
N*‐*Myc_73‐94_	Ac‐ EPPSWVTEMLLENELWGSPAEE ‐NH_2_	199±13	−4235±181	13±1
N*‐*Myc_73‐97_	Ac‐ EPPSWVTEMLLENELWGSPAEEDAF ‐NH_2_	79±4	−4886±55	14±1
N*‐*Myc_73‐89‐E73pS_	Ac‐ pSPPSWVTEMLLENELWG ‐NH_2_	140±18	−5394±235	16±1
N*‐*Myc_73‐89‐E73S_	Ac‐ SPPSWVTEMLLENELWG ‐NH_2_	217±13	−5541±66	17±1

[a] One letter code for amino acids. [b] IC_50_ values given as the mean value and corresponding standard deviation (SD) determined from triplicate competition FA assays against fluorescein labelled WT N*‐*Myc_61‐89_ FAM (50 nM) in the presence of Aurora‐A_122‐403C290A/C393A_ (15 μM) (n=3). All assays were performed in 25 mM Tris, 150 mM NaCl, 5 mM MgCl_2_, pH 7.5 and left to equilibrate for 2 h at room temperature before measuring (Figure S1 for data). [c] Mean residue ellipticity and [d] estimated % Helicity as measured in 25 mM Tris, 150 mM NaCl, 5 mM MgCl_2_, pH 7.5 at 5 °C by CD spectroscopy and calculated using equations 6 and 7 (n=2).

### Analyses of constrained peptides by fluorescence anisotropy competition and circular dichroism

The effects of introducing a chemical constraint within the helical region of N*‐*Myc_61‐89_ were next explored. To identify suitable positions for introduction of a constraint, we adopted a systematic approach.[^13^, ^14^] In this approach, cysteine pairs were introduced in the helical region of N*‐*Myc_61‐89_ at all non‐hot‐spot residues at *i* and *i*+4 spacings, to give a series of 7 variants (see ESI, Table S1). Each variant was then constrained using dibromomaleimide[^15^, ^16^] to afford the maleimide (**mal**) constrained variants (Figure 1b), or allowed to oxidize to yield the disulfide bridged variant (**ox**), or reduced to its free‐thiol form (**red**). The sequences of the resultant 21 variants along with their measured IC_50_, MRE_222_ values and % helicity estimates (determined using circular dichroism spectroscopy) are given in the supporting information (Table S1 and Figures S2–S4). In all cases, the maleimide constraint was found to have a limited or negative impact on the inhibitory potency of the peptide. However, we found that some positions were more tolerant to modification than others, with constrained peptides N*‐*Myc_61‐89‐L82C/E86C‐**mal**_, N*‐*Myc_61‐89‐E80C/E84C‐**mal**_, and N*‐*Myc_61‐89‐T79C/L83C‐**mal**_ showing only minor loss in potency when compared to the native peptide.

Although we found that extending of the N*‐*Myc helical region by an additional five residues at the C‐terminus has a limited effect in terms of potency (N*‐*Myc_73‐94_; IC_50_=199±13 μM, Table 1), this sequence offered additional positions for the introduction of a constraint in comparison to N*‐*Myc_61‐89_ (note: N‐Myc_73‐97_ had higher potency, however our motivation was to identify the shortest sequence into which a constraint could be productively incorporated). More specifically, constrained variants involving the replacement of the *i* and *i*+4 pairs: Asn85^N‐Myc^/Gly89^N‐Myc^, and Glu86^N‐Myc^/Ser90^N‐Myc^, could be explored. These pairs of residues were replaced with cysteines, and the peptides generated were constrained (**mal**), oxidized (**ox**), or reduced (**red**). The sequences and key CD spectral data for these 6 new variants are shown in Table 2. Using competition FA, both new maleimide constrained variants showed improved IC_50_ potencies compared to the unmodified peptide (N*‐*Myc_73‐94_ IC_50_=199±13 μM; Table 2, Figure S5). Indeed, maleimide constrained N*‐*Myc_73‐94‐N85C/G89C‐**mal**_ (IC_50_=49±5 μM) was found to exhibit 4‐fold improvement (ΔΔG ~3.5 kJ mol^−1^ relative to N*‐*Myc_73‐94_) in potency to disrupt N*‐*Myc/Aurora‐A compared to the WT peptide. The inhibitory potency exhibited by the constrained N*‐*Myc_73‐94‐N85C/G89C‐**mal**_ variant was found to be in the same range as that observed for the longer WT sequence (N*‐*Myc_61‐89_, IC_50_=42±4 μM; Table 1), despite lacking the N*‐*terminal turn region and being ~25 % shorter. The second variant, N*‐*Myc_73‐94‐E86C/S90C‐**mal**_ (IC_50_=111±18 μM) was also found to be 2‐fold more potent than the control peptide. For the most potent sequence (N*‐*Myc_73‐94‐N85C/G89C‐**mal**_), a number of features should be noted: (i) Gly89^N‐Myc^ is replaced with cysteine to introduce the **mal** constraint and glycine is known to have low helix propensity;[^17^, ^18^] Ser90^N‐Myc^ has the potential to act as a helix‐capping residue^[19]^ before Pro91^N‐Myc^ which likely interrupts the helical structure.[^17^, ^20^] Finally, based on the Aurora‐A/N*‐*Myc crystal structure, a **mal** constraint introduced at Asn85^N‐Myc^ and Gly89^N‐Myc^ is unlikely to be completely solvent‐exposed and may form direct contacts with the surface of Aurora‐A surface. Overall, these results highlight a crucial role in selecting an appropriate sequence length to allow identification of optimal sites for constraining peptide ligands.

**Table 2:** Effects of sequence modification, oxidation and maleimide constraint in N*‐*Myc_73‐94_.

Peptide	Sequence^[a]^	IC_50_^[b]^ (μM)	MRE_222_^[c,e]^ (deg cm^−1^ dmol^−1^ res^−1^)	% Helicity^[d,e]^
N*‐*Myc_73‐94_ WT	Ac‐ EPPSWVTEMLLENELWGSPAEE ‐NH_2_	199±13	−4235±181	13±1
N*‐*Myc_73‐94‐N85C/G89C_ (red)	Ac‐ EPPSWVTEMLLE **C** ELW **C** SPAEE ‐NH_2_	123±10	−4383±64^[e]^	13±1
N*‐*Myc_73‐94‐N85C/G89C_ (ox)	Ac‐ EPPSWVTEMLLE **C** ELW **C** SPAEE ‐NH_2_	130±9	−5255±217	15±1
N*‐*Myc_73‐94‐N85C/G89C_ (mal)	Ac‐ EPPSWVTEMLLE **C** ELW **C** SPAEE ‐NH_2_	49±5	−7223±168	21±1
N*‐*Myc_73‐94‐E86C/S90C_ (red)	Ac‐ EPPSWVTEMLLEN **C** LWG **C** PAEE ‐NH_2_	203±4	−6397±64^[e]^	18±1
N*‐*Myc_73‐94‐E86C/S90C_ (ox)	Ac‐ EPPSWVTEMLLEN **C** LWG **C** PAEE ‐NH_2_	172±23	−7454±130	21±1
N*‐*Myc_73‐94‐E86C/S90C_ (mal)	Ac‐ EPPSWVTEMLLEN **C** LWG **C** PAEE ‐NH_2_	111±18	−5715±64	17±1

[a] One letter code for amino acids. Highlighted in grey: free‐SH cysteines; in gold: disulfide bridged cysteines −S−S− and in magenta: maleimide constrained cysteines. [b] IC_50_ values given as the mean value and corresponding standard deviation (SD) determined from triplicate competition FA assays against fluorescein labelled WT N*‐*Myc_61‐89_ FAM (50 nM) in the presence of Aurora‐A_122‐403‐C290A/C393A_ (15 μM) (n=3). All assays were performed in 25 mM Tris, 150 mM NaCl, 5 mM MgCl_2_, pH 7.5 and left to equilibrate for 2 hours at room temperature before measuring. [c] Mean residue ellipticity and [d] estimated % Helicity as measured in 25 mM Tris, 150 mM NaCl, 5 mM MgCl_2_, pH 7.5 at 5 °C by CD spectroscopy and calculated using equations 6 and 7 (n=2). [e] 5 mol equivalents of TCEP were added to the samples to ensure that no oxidized species were present.

**Figure 2:**
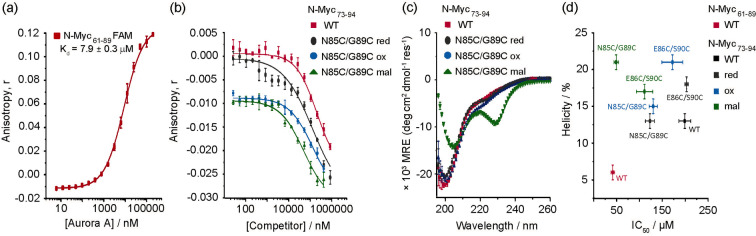
**FA assays**, **circular dichroism and potency vs. secondary structure correlations in N** *‐* **Myc_73‐94_ variants**: (a) FA direct titration of FAM‐Ahx‐N‐Myc_61‐89_ with Aurora‐A_122‐403‐C290A/C393A_ ; (b) Competition FA results for peptides in Table 2 against N*‐*Myc_61‐89_ (25 mM Tris, 150 mM NaCl, 5 mM MgCl_2_, pH 7.5, 15 μM Aurora‐A_122‐403‐C290A/C393A_, 50 nM FAM‐Ahx‐N‐Myc_61‐89_, 25 °C). (c) Comparative CD spectra of N*‐*Myc_73‐94_ WT vs. N*‐*Myc_73‐94‐N85C/G89C_ reduced (**red** in grey), oxidized (**ox** in blue) and maleimide constrained (**mal** in green) variants. (d) Plot of % helicity against IC_50_ values for N*‐*Myc_73‐94_ and its variants shown in Table 2.

Next, we assessed if the effects observed on peptide potency upon constraining could be correlated with changes in peptide conformation (Figure 2). The control N*‐*Myc_73‐94_ and N*‐*Myc_73‐94‐E86C/S90C_ variants showed similar CD spectra, characterized by the presence of a minima in mean residue ellipticity (MRE) at a wavelength of ∼200 nm, slightly shifted to ∼205 nm for N‐Myc_73‐94‐E86C/S90C‐**ox**_ (Figure S5 and S6). Qualitatively, the variants exhibited only limited differences in helicity, with all adopting predominantly random coil conformations in solution (estimated % helicity ~17 to 21; Table 2). In contrast, N*‐*Myc_73‐94‐N85C/G89C‐**mal**_ exhibited a different CD spectrum, indicating that the **mal** constraint in this position destabilizes the random coil conformation of the peptide and biases it in favor of another defined conformation (Figure 2c). Both the shift in minima to higher wavelength (from ~200 nm to ~205 nm) and the increase in positive signal at 193 nm are indicative of increased α‐helix propensity (% helicity=21±1; Table 2). However, the emergence of a negative signal at 230 nm was unexpected, as it has been proposed to be more characteristic of “turn” like structures (although a positive signal at ~215 nm, also characteristic of turn structures is absent).^[21]^ The signal at 230 nm may arise as a consequence of the interaction between the chromophores of the **mal** group and the peptide backbone. In this regard, abnormal CD spectra have been associated with the effects of other conformational constraints.^[22]^ Overall, the data indicates that the conformational ensemble has reduced random coil character.

**Figure 3:**
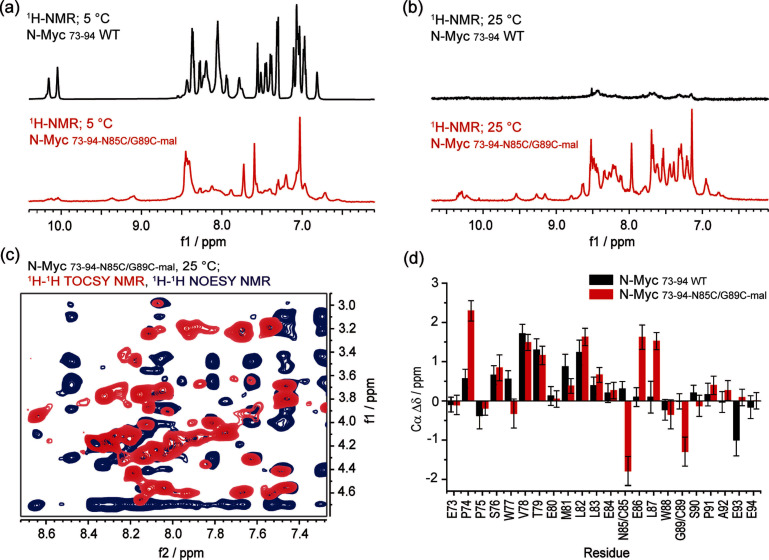
**NMR secondary structure analysis of N** *‐* **Myc_73‐94_ and constrained N** *‐* **Myc_73‐94‐N85/G89C‐mal_**. (a) ^1^H‐NMR spectra of N*‐*Myc_73‐94_ (in black) and N*‐*Myc_73‐94‐N85/G89C‐**mal**_ (in red) showing the amide‐bond region of the peptides as observed at 5 °C; (b) ^1^H‐NMR trace of N*‐*Myc_73‐94_ (in black) and N*‐*Myc_73‐94‐N85/G89C‐**mal**_ (in red) showing the amide‐bond region of the peptides as observed at 25 °C; (c) Superposition of the ^1^H‐^1^H TOCSY (in red) and ^1^H‐^1^H NOESY spectra (in blue) at the NH−Hα region of N*‐*Myc_73‐94‐N85/G89C‐**mal**_; (d) Secondary Δδ_Cα_ chemical shifts by residue calculated for N*‐*Myc_73‐94_ at 5 °C (black bars) and N*‐*Myc_73‐94‐N85/G89C‐**mal**_ at 25 °C (red bars) as based in their NMR ^1^H and ^1^H‐^13^C HSQC spectra (error bars derive from variation in peak width). Note resonances from 9.00–10.5 correspond to exchangeable indole NH protons and other unassigned exchangeable NH/OH protons.

Analyses of the potency vs. helicity data for N*‐*Myc_73‐94_ based peptides (Figure 2d, Table 2) revealed that the **mal** constrained analogue for the N*‐*Myc_73‐94‐E86C/S90C_ series is the most potent, despite little difference in helicity relative to the other variants. For N*‐*Myc_73‐94‐N85C/G89C_ variants, a clearer correlation is observed with both the free‐thiol (red) and disulfide bridged (**ox**) analogues displaying similar potency and helicity, and the **mal** variant exhibiting an increase in both potency and helicity (Figure 2c). Overall, the full dataset for all peptides studied indicates a complex relationship between constraint position, peptide conformation and inhibitory potency (Tables 2, S1 and Figures 3d, S7).

### Comparison of N‐Myc_73‐94‐N85C/G89C‐mal_ and N‐Myc_73‐94_ by NMR analyses

Given the higher potency and unique CD spectrum observed for N*‐*Myc_73‐94‐N85C/G89C‐**mal**_ when compared to the linear WT sequence, we further investigated the solution structure of the unbound peptide by NMR spectroscopy. Samples of N*‐*Myc_73‐94_ and N*‐*Myc_73‐94‐N85C/G89C‐**mal**_ were prepared, and their spectra analyzed at 5 °C. The NMR spectra for N*‐*Myc_73‐94_ at this temperature showed well resolved ^1^H resonances (Figure 3a), whilst constrained N*‐*Myc_73‐94‐N85C/G89C‐**mal**_ exhibited broadened resonances of lower intensity (Figures S8–18 for full spectra at 5 °C). Although TOCSY spectra for both peptides had some similarity (Figure S18) many N*‐*Myc_73‐94‐N85C/G89C‐**mal**_^1^H signals were not observable (Cys *res* 85 and 89) precluding full assignment (see Figures S16–18). We hypothesize such behavior arises as a consequence of the maleimide constraint restricting the rate of interconversion between different conformers such that they match the NMR timescale, broadening the resonances to the point of coalescence. Such behavior has previously been observed in molten globules and partially folded proteins.[^23^, ^24^] To confirm this hypothesis, we carried out ^1^H variable temperature (VT) NMR experiments on both samples (Figure 3a–b, Figures S19–22). Upon increasing the temperature, we observed changes in the unconstrained peptide (N*‐*Myc_73‐94_); an increase in temperature of only 5 °C was sufficient to induce broadening of the ^1^H resonances (Figures S19–20). Different behavior was found for the constrained peptide (N*‐*Myc_73‐94‐N85C/G89C‐**mal**_): upon gradually increasing the temperature from 5 to 25 °C, we observed a clear shift to higher signal intensities accompanied by resonance narrowing and improved resolution, particularly in the amide NH and Hα regions (Figure S21–22). Reducing the temperature to 0 or −3 °C restored the exchange/broadening effect, supporting the notion that slow exchange between conformers occurs.

Complete ^1^H/^13^C NMR assignment of the constrained peptide could be achieved at 25 °C using the corresponding TOCSY/NOESY spectra (Figure 3c; Figures S23–29). To gain further structural insight on N*‐*Myc_73‐94_ and N*‐*Myc_73‐94‐N85C/G89C‐**mal**_, their Cα secondary shifts (Δδ_Cα_) were calculated using published random coil Cα shift (δ_RC_) values for disordered proteins (Figure 3d, note: in the absence of a reference value for a maleimide linked cysteine residue, the value for cysteine was used).^[25]^ Cα carbons experience a downfield shift when they are located in α‐helical regions[^26^, ^27^, ^28^] and it has been shown that Cα secondary shifts, Δδ_Cα_ (i. e. the observed Cα shifts, δ – the expected Cα shifts for that residue in a random coil conformation, δ_RC_), offer an accurate predictor of secondary structure.^[28]^ A continuous series of downfield (*i. e*. positive) Cα secondary shifts were observed for WT N*‐*Myc_73‐94_ between Ser76^N‐Myc^ and Leu87^N‐Myc^ at 5 °C, indicating propensity for helix‐formation (Figure 3d, Table S2).^[28]^ Of note, the magnitude of the observed downfield Cα secondary shifts (Δδ_Cα_≤0.5–1.5 ppm) are lower than those typically seen in heavily stabilized α‐helical structures (Δδ_Cα_≥2.0–3.5 ppm),^[29]^ in agreement with the predominantly random coil conformation of N*‐*Myc_73‐94_ observed by CD (Figure 2). The downfield shifts are particularly pronounced at the N‐terminus of the proposed α‐helical region (Ser76^N‐Myc^ to Gly89^N‐Myc^ based on the crystal structure; Figure 3e), with Val78^N‐Myc^, Thr79^N‐Myc^, Met81^N‐Myc^, and Leu82^N‐Myc^ affording the largest Δδ_Cα_. Towards the C‐terminus, the magnitude of Cα secondary shifts decreases considerably to Δδ_Cα_ ~0 ppm, indicating unwinding around Leu87^N‐Myc^/Trp88^N‐Myc^.

For N*‐*Myc_73‐94‐N85C/G89C‐**mal**_ at 25 °C, as for the wild type peptide at a 5 °C, the data suggest there may be secondary structure propensity despite the increase in temperature. When compared, both peptides showed a similar trend in the relative magnitude of Cα secondary shifts at each residue (Figure 3d, Table S3). The most notable divergence between the peptides is observed in the constrained N*‐*Myc region corresponding to residues 85–89, where relatively high Cα secondary shifts were observed for Glu86^N‐Myc^/Leu87^N‐Myc^. This indicates the introduction of the chemical constraint may exert a helix stabilizing effect in a region where the WT peptide shows low helical propensity (*i. e*., Δδ~δ_RC_). Both maleimide derivatized residues, Cys85^N‐Myc^ and Cys85^N‐Myc^, exhibited two out‐of‐trend negative Δδ_Cα_ values, suggesting that their secondary chemical shifts are either highly affected by the presence of the constraint, or misrepresented due to the lack of an appropriate δ_RC_ reference for the modified amino‐acid. ^1^H secondary shifts were also calculated for both peptides (Figures S30–31); regions of continuous up‐field (i. e. negative) shifts indicate helicity.[^25^, ^27^] In agreement with Cα secondary shifts, a continuous series of up‐field Δδ_Hα_ secondary shifts were observed between Glu73^N‐Myc^ and Gly89^N‐Myc^ for the linear control peptide at 5 °C, of relatively small magnitude (Δδ_Hα_~0.2), indicating weak helix propensity. The calculated Δδ_Hα_ values were indicative of two regions: one at the N‐terminus of the peptide displaying higher Δδ_Hα_ values around Val78^N‐Myc^, and a second towards the C‐terminus, where consistently lower Δδ_Hα_ are observed. For the constrained peptide at 25 °C we found a similar general trend, with Δδ_Hα_ values in the same range but slightly lower in magnitude.

### Molecular dynamics (MD) analyses on N‐Myc_73‐94‐N85C/G89C‐mal_ and N‐Myc_73‐94_

**Figure 4:**
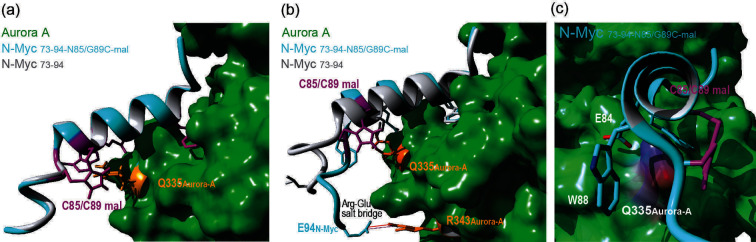
**MD studies of N** *‐* **Myc_73‐94_ and constrained N** *‐* **Myc_73‐94‐N85/G89C‐mal_ in the presence of Aurora‐A**: (a) Overlay of the minimum energy structures observed for N*‐*Myc_73‐94_ (in grey) and N*‐*Myc_73‐94‐N85/G89C‐**mal**_ (in cyan) bound to Aurora‐A (the maleimide constraint and key interacting residues are highlighted in purple); (b) Average structures of unmodified N*‐*Myc_73‐94_ (shown in grey) and N*‐*Myc_73‐94‐N85/G89C‐**mal**_ (shown in cyan) in the presence of Aurora‐A (the maleimide constraint is shown highlighted in purple); (c) Axial view of the averaged structure of N*‐*Myc_73‐94‐N85/G89C‐**mal**_ (in cyan) in the presence of Aurora‐A showing the arrangement of the maleimide group and key residues (W88 and E84) around Gln335_AurA_.

Finally, to investigate how the constraint might affect the secondary structure of the peptide in the presence of Aurora‐A, we employed MD analysis (Figure 4, Figures S32–S37).^[14]^ We based our analyses on the reported X‐ray structure of the N*‐*Myc/Aurora‐A complex (PDB : 5G1X),^[7]^ where the sequence of N*‐*Myc was extended at the C‐terminus to include either the natural residues 89–94 or the maleimide constrained G85C/N89C fragment. Results from this analysis were broadly consistent with the structural insights inferred using both CD spectra and NMR techniques, with N*‐*Myc_73‐94_ showing two well defined regions in its minimum energy structure: a helical segment spanning the N‐terminus – residues 73–86 – and a small transient turn region – residues 88–94 – that seems to wrap around the Aurora‐A αG‐helix (Figure 4a, Figure S30). MD simulations for N*‐*Myc_73‐94‐N85C/G89C‐**mal**_ revealed a similar and consistent energy minimum Aurora‐A bound structure to that observed for the linear peptide, indicating that both peptides could access similar conformations in their Aurora‐A bound states (Figure 4a and Figures S32–33). We also observed that the maleimide group sits orientated directly towards Aurora‐A in proximity to Gln335^AurA^, which seems to be conformationally trapped between the maleimide and residues Trp88^N‐Myc^ and Glu84^N‐Myc^ in N‐Myc. Analyses of the time averaged structure of both peptides bound to Aurora‐A (Figure 4b–c) revealed that for N*‐*Myc_73‐94_ fraying of the helix occurs towards the C‐terminus, whilst in comparison, the average structure observed for constrained N*‐*Myc_73‐94‐N85C/G89C‐**mal**_ remains closer to the energy minimum state, with the maleimide constraint potentially interacting via a π‐amide interaction with Gln335^AurA^ and preventing the C‐terminus from unfolding and undocking from the protein surface. In addition, results from these models also suggest that such an arrangement, where the C‐terminus is kept in proximity to Aurora‐A due to the maleimide constraint, enables a transient/dynamic close electrostatic interaction between Glu94^N‐Myc^ and Arg343^AurA^, which does not take place in the WT peptide due to helix unwinding.

## Conclusions

In summary, here we show for the first time the systematic application of maleimide‐constraining to identify peptidomimetic leads for inhibition of the N*‐*Myc/Aurora‐A interaction. Using N*‐*Myc_61‐89_ as a starting point and through rational evaluation of the effects of sequence truncation, elongation, and maleimide constraining we identified N*‐*Myc_73‐94‐N85C/G89C‐**mal**_ as a minimal constrained peptide with enhanced inhibitory potency when compared to its linear parent peptide. Correlation between potency (determined by competition FA) and conformational analyses (by CD) indicated complex structure/activity relationships for the interaction of all the different N*‐*Myc variants with Aurora‐A, suggesting that α‐helicity of the peptides does not solely determine inhibitory potency. Indeed, the most potent lead, N*‐*Myc_73‐94‐N85C/G89C‐**mal**_, was found to exhibit a unique CD spectrum; this may represent a maleimide‐stabilized turn‐like secondary structure although this could also arise from exciton coupling with the maleimide group, however, the data is consistent with reduced random coil character. Overall, our results suggest that the constraint restricts the accessible conformational landscape of the peptide to an ensemble which is more compatible with recognition of Aurora‐A. This in combination with enhanced helicity at the C‐terminus, in the Aurora‐A bound complex and additional non‐covalent contacts between constraint and protein as observed in MD simulations provide a plausible explanation for the enhanced potency of N*‐*Myc_73‐94‐N85C/G89C‐**mal**_. Overall, and excitingly, our work reports the first steps towards the rational structure/folding‐guided development of constrained peptide inhibitors of the N*‐*Myc/Aurora‐A PPI as potential anticancer therapeutics. We will report on further efforts towards this goal in due course.

## Experimental Section

**Peptide synthesis and purification**: The peptides were synthesised using standard Fmoc‐based solid‐phase methods manually or on a LibertyBlue microwave‐assisted automated peptide synthesiser (CEM; Mathews, NC, USA). In manual syntheses, 20 % piperidine in DMF was used for Fmoc deprotection and DIPEA (6 equiv.)/HCTU (4.5 equiv.) were used for amino acid (3 equiv.) coupling at room temperature for 1 h in each step. In automated syntheses, Fmoc deprotection was achieved by treatment with 20 % piperidine and 5 % formic acid in DMF under microwave‐assisted conditions. Oxyma/DIC was used for amino acid coupling with standard microwave‐assisted coupling methods. 5(6)‐carboxyfluorescein (4.5 equiv.) was added using Oxyma (4.5 equiv.)/DIC (6 equiv.) at room temperature overnight. Peptides were acetylated using acetic anhydride (10 equiv.) and DIPEA (10 equiv.) in DMF for 40 min. All peptides were cleaved by the cleavage cocktail (TFA : H2O : TIPS : DODT=92.5 : 2.5 : 2.5 : 2.5) for 3 h at room temperature and precipitated using excess cold diethyl ether. Crude peptides were purified on a 1260 Infinity II preparative HPLC system (Agilent; Santa Clara, CA, USA). Purified peptides were isolated by lyophilisation.

**Peptide maleimide constraining and oxidation**: Peptide constraining was performed in acetonitrile/phosphate buffer (ratio 2 : 1; peptide concentration 4 mg mL^−1^; phosphate buffer: 20 mM phosphate, 150 mM NaCl, pH=7.8. TCEP (solution in buffer, 2 equiv.) was added, and the peptide was agitated for 30 min to reduce any disulfide bonds. Thereafter, dibromomaleimide (solution in acetonitrile, 2 equiv.) was added and the peptide was agitated for 2 h. The reaction was monitored by LC–MS. Upon completion of the reaction, peptides were purified by preparative HPLC and freeze‐dried. Oxidation of the peptides to their disulfide bridged variants was accomplished by direct air oxidation of the pure free sulfhydryl materials in buffer at room temperature overnight.

**Fluorescence anisotropy (FA) assays**: All assays were performed using 384‐well plates (Greiner Bio‐one, UK). Aurora‐A _122‐403‐C290A/C393A_ protein was produced as described previously.^[30]^ All samples were prepared in 25 mM Tris, 150 mM NaCl, 5 mM MgCl_2_, pH 7.5, and tested in triplicate using an EnVisionTM 2103 MultiLabel plate reader (Perkin Elmer; Waltham, MA, USA). The parameters were set as follows: Excitation wavelength=480 nm (30 nm bandwidth) and emission wavelength=535 nm (30 nm bandwidth). Measured data were processed and analysed as previously described.^[31]^ Specifically, the perpendicular intensity (*P*) and parallel intensity (*S*) were subtracted by the control values and used for calculations of intensity and anisotropy using the following Equations 1, 2, 3, 4, and 5:

(1) $$I=\left(2PG\right)+S$$

(2) $$r=\left(S-PG\right)$$

(3) $$L_{b}=\frac{r-r_{min}}{\lambda \left(r_{max}-r\right)+r-r_{min}}$$

(4)

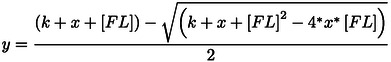

(5) $$y=r_{max}+\frac{r_{min}-r_{max}}{1+{\left(x/x_{0}\right)}^{p}}$$

Where *I*=total intensity; *P*=perpendicular intensity; *S*=parallel intensity; *G*=instrument factor; *r*=anisotropy; *L*_b_=ligand bound fraction; λ=change in intensity between bound and unbound states which was 1 in this instance, [*FL*]=fluorescent ligand concentration; *k*=*K*_d_; *y*=*L*_b_ *[*FL*] and *x*=added protein concentration. For competition FA assays, the average anisotropy and the average standard deviation of the values derived from equation (2) were calculated and fit to a sigmoidal logistic model (equation (5)) using Origin 2021. For peptides where full displacement was not observed, a minimum anisotropy r_min_=−0.03 (obtained for peptides that did achieve full displacement) was used to restrain the fitting. Results are reported as IC_50_±SD, with data points representing the mean of three replicates and error bars indicating the corresponding SD. Where applicable, ΔΔG was determined as follows: ΔΔG=−RT ln (ΔIC_50_).

**Circular dichroism (CD)**: All samples were prepared in Tris buffer (25 mM Tris, 150 mM NaCl, 5 mM MgCl_2_, pH 7.5) with the exception of free sulfhydryl peptides, to which Tris(2‐carboxyethyl)phosphine (TCEP; 5 mol eq. relative to the peptide) was added. CD spectra were recorded using an APP Chirascan CD spectropolarimeter and 1 mm pathlength quartz cuvettes. Sample concentrations were typically 30–120 μM, as determined by UV−V absorption based on a tryptophan extinction coefficient of 5600 M^−1^ cm^−1^ and, for mal‐constrained variants, a maleimide coefficient of 1700 M^−1^ cm^−1^.[^16^, ^32^] Once diluted to this degree samples had a pH matching the buffer alone. Spectra were recorded in duplicate at 5 °C, over wavelengths ranging from 290 to 180 nm, with data collected every 1 nm (1 nm s^−1^). A background spectrum for CD buffer alone was also recorded and background ellipticity values were subtracted from raw sample ellipticity values (*θ*) when calculating mean residue ellipticities (MREs) using Equation 6:

(6) $$MRE=\frac{{(\left[\theta \right]}_{\lambda }-{\left[\theta \right]}_{0})\;x\;M_{w}}{n\;x\;l\;x\;c}$$

Where [*θ*]_λ_ is the observed ellipticity at a given wavelength λ in mdeg, [*θ*]_0_ is the ellipticity observed for the buffer, *M_w_* =molar weight of the peptide in g mol^−1^, *n*=number of residues; *c*=sample concentration (mg mL^−1^); *l*=pathlength of the cuvette in mm. Estimates of peptide % helicity were made using Equation 7, ^[33]^

(7) $$\%\;helicity=\frac{{(MRE}_{222}-{MRE}_{coil})\;x\;100\;}{-42500\left(1-\left(\frac{3}{n}\right)\right)}$$

where MRE_222_ is the MRE value at 222 nm, MRE_coil_=640‐45*T* (with *T* in °C)=415 deg cm^2^ dmol^−1^ res^−1^ at 5 °C, and *n* is the number of backbone amide bonds including the N‐terminal acetyl.

**Peptide NMR analysis and secondary chemical shift calculation**: NMR studies were recorded on a Bruker AV4 NEO 11.75 T (500 MHz ^1^H) NMR spectrometer (500‐4C) at either 278 or 298 K, using water suppression by means of excitation sculpting with gradients using perfect echo.[^34^, ^35^] All samples were prepared in a buffer/D_2_O mixture (90/10 vol/vol) to achieve a final concentration of 3 mM (buffer: 25 mM potassium phosphate, 50 mM NaCl, 5 mM MgCl_2,_ pH=7.5). For each sample a complete set of experiments was performed, where ^1^H‐NMR, ^1^H‐^1^H TOCSY (20 and 80 ms), and ^1^H‐^1^H COSY were employed to assign the identity of each amino acid present in the peptide sequence, and ^1^H‐^1^H NOESY was employed to establish the inter‐residue connectivity and spatial correlations. Folded and unfolded ^1^H‐13C{^1^H} HSQC spectra were used in all cases to characterize the ^13^C nuclei and to support full ^1^H assignment. Secondary shifts for Cα and Hα nuclei were calculated by subtraction of coil values from measured shifts for each residue.^[25]^

**Molecular dynamics (MD) analysis**: All peptide–protein complexes were subjected to duplicate MD simulations using YASARA structure.^[36]^ Maleimide constraints were modelled in YASARA by swapping the pertinent amino acids for cysteines and connecting to maleimide fragments. In addition, N*‐*Myc_73‐94_ (residues 90–94 not resolved in the original crystal structure) were modelled prior to any analysis using helical dihedral angles and then minimised structures were generated using the energy minimization function with default settings. The modelled complexes were subjected to MD simulations using YASARA structure macro for fast MD run (www.yasara.org/md_runfast.mcr).^[37]^ Briefly, the AMBER14 forcefield was used and the temperature was set as 298.0 K with the timestep set as 1*2.50 fs. Each complex was run for 80 ns. Minimum energy and average structures were analysed from these experiments and figures created using the same software.

## Supporting Information

The authors have cited additional references within the Supporting Information.[^38^, ^39^]

## Conflict of interest

The authors declare no conflict of interest.

1

## Supporting information

As a service to our authors and readers, this journal provides supporting information supplied by the authors. Such materials are peer reviewed and may be re‐organized for online delivery, but are not copy‐edited or typeset. Technical support issues arising from supporting information (other than missing files) should be addressed to the authors.

Supporting Information

Supporting Information

Supporting Information

## Data Availability

The data that support the findings of this study are available in the supplementary material of this article.
