# Supplementary material for: Inhibition of Aurora‐A/N‐Myc Protein–Protein Interaction Using Peptidomimetics: Understanding the Role of Peptide Cyclization
Source: Chembiochem. 2023 Nov 27;25(2):e202300649. doi: 10.1002/cbic.202300649 (PMC10962542; doi:10.1002/cbic.202300649)

ChemBioChem

Supporting Information

Inhibition of Aurora-A/N-Myc Protein–Protein Interaction Using Peptidomimetics: Understanding the Role of Peptide Cyclization

Robert S. Dawber, Diana Gimenez, Matthew Batchelor, Jennifer A. Miles, Megan H. Wright, Richard Bayliss,* and Andrew J. Wilson*

Supplementary data and figures..........2
Supplementary methods..........33
Abbreviations..........33
References..........33
Analytical HPLC traces and high-resolution mass spectra of synthesised peptides..........35

Supplementary data and figures

Peptide[a]	Sequence			IC_{50}[b] (µM)	MRE_{222}[c] (deg cm^{-1} $dmol^{-1}$ res^{-1})	% Helicity[d]
N-Myc $_{61-89}$	Ac-	LSPSRGFAEHSSEPPSWVTEMLLENELWG	-NH$_2$	42 ± 4	-2059 ± 64	6 ± 1
N-Myc $_{61-89}^{L83C/L87C}$ red	Ac-	LSPSRGFAEHSSEPPSWVTEMLCENECWG	-NH$_2$	181 ± 9	-1746 ± 74	6 ± 1
N-Myc $_{61-89}^{L83C/L87C}$ ox	Ac-	LSPSRGFAEHSSEPPSWVTEMLCENECWG	-NH$_2$	103 ± 11	-2223 ± 37	7 ± 1
N-Myc $_{61-89}^{L83C/L87C}$ mal	Ac-	LSPSRGFAEHSSEPPSWVTEMLCENECWG	-NH$_2$	129 ± 3	-1947 ± 80	6 ± 1
N-Myc $_{61-89}^{L82C/E86C}$ red	Ac-	LSPSRGFAEHSSEPPSWVTEMCLENCLWG	-NH$_2$	120 ± 14	-2348 ± 49	7 ± 1
N-Myc $_{61-89}^{L82C/E86C}$ ox	Ac-	LSPSRGFAEHSSEPPSWVTEMCLENCLWG	-NH$_2$	219 ± 23	-2630 ± 119	8 ± 1
N-Myc $_{61-89}^{L82C/E86C}$ mal	Ac-	LSPSRGFAEHSSEPPSWVTEMCLENCLWG	-NH$_2$	69 ± 18	-2581 ± 28	8 ± 1
N-Myc $_{61-89}^{M81C/N85C}$ red	Ac-	LSPSRGFAEHSSEPPSWVTECLLECELWG	-NH$_2$	164 ± 6	-2198 ± 93	7 ± 1
N-Myc $_{61-89}^{M81C/N85C}$ ox	Ac-	LSPSRGFAEHSSEPPSWVTECLLECELWG	-NH$_2$	92 ± 13	-2894 ± 119	9 ± 1
N-Myc $_{61-89}^{M81C/N85C}$ mal	Ac-	LSPSRGFAEHSSEPPSWVTECLLECELWG	-NH$_2$	133 ± 3	-3783 ± 38	11 ± 1
N-Myc $_{61-89}^{E80C/E84C}$ red	Ac-	LSPSRGFAEHSSEPPSWVTCMLLCNELWG	-NH$_2$	111 ± 5	-2562 ± 106	8 ± 1
N-Myc $_{61-89}^{E80C/E84C}$ ox	Ac-	LSPSRGFAEHSSEPPSWVTCMLLCNELWG	-NH$_2$	107 ± 19	-2656 ± 28	8 ± 1
N-Myc $_{61-89}^{E80C/E84C}$ mal	Ac-	LSPSRGFAEHSSEPPSWVTCMLLCNELWG	-NH$_2$	86 ± 7	-4575 ± 35	13 ± 1
N-Myc $_{61-89}^{T79C/L83C}$ red	Ac-	LSPSRGFAEHSSEPPSWVCEMLCENELWG	-NH$_2$	152 ± 15	-1907 ± 23	6 ± 1
N-Myc $_{61-89}^{T79C/L83C}$ ox	Ac-	LSPSRGFAEHSSEPPSWVCEMLCENELWG	-NH$_2$	75 ± 8	-1951 ± 84	6 ± 1
N-Myc $_{61-89}^{T79C/L83C}$ mal	Ac-	LSPSRGFAEHSSEPPSWVCEMLCENELWG	-NH$_2$	99 ± 2	-3781 ± 69	11 ± 1
N-Myc $_{61-89}^{V78C/L82C}$ red	Ac-	LSPSRGFAEHSSEPPSWCTEMCLENELWG	-NH$_2$	≥ 500	-1692 ± 70	6 ± 1
N-Myc $_{61-89}^{V78C/L82C}$ ox	Ac-	LSPSRGFAEHSSEPPSWCTEMCLENELWG	-NH$_2$	≥ 500	-2823 ± 48	8 ± 1
N-Myc $_{61-89}^{V78C/L82C}$ mal	Ac-	LSPSRGFAEHSSEPPSWCTEMCLENELWG	-NH$_2$	≥ 500	-1767 ± 72	6 ± 1
N-Myc $_{61-89}^{S76C/E80C}$ red	Ac-	LSPSRGFAEHSSEPPCWVTCMLLENELWG	-NH$_2$	140 ± 18	-2428 ± 25	7 ± 1
N-Myc $_{61-89}^{S76C/E80}$ ox	Ac-	LSPSRGFAEHSSEPPCWVTCMLLENELWG	-NH$_2$	84 ± 9	-2141 ± 88	7 ± 1
N-Myc $_{61-89}^{S76C/E80}$ mal	Ac-	LSPSRGFAEHSSEPPCWVTCMLLENELWG	-NH$_2$	147 ± 13	-4939 ± 128	14 ± 1

Table S1. Systematic N-Myc $_{61-89}$ maleimide constraining scan. [a] One letter code for amino acids. Highlighted in grey : free -SH cysteines; in gold: disulphide bridged cysteines -S-S- and in purple: maleimide constrained cysteines. [b] IC_{50} values given as the mean value and corresponding standard deviation (SD) determined from triplicate competition FA assays against fluorescein labelled WT N-Myc $_{61-89}$ FAM (50 nM) in the presence of Aurora-A $_{122-403-C290A/C393A}$ (15 µM)(n= 3). All assays were performed in 25 mM Tris, 150 mM NaCl, 5 mM $MgCl_2$, pH 7.5 and left to equilibrate for 2 hours at room temperature before measuring. [c] Mean residue ellipticity and [d] % Helicity as measured in 25 mM Tris, 150 mM NaCl, 5 mM $MgCl_2$, pH 7.5 at 5 °C by CD spectroscopy and calculated using equations 6 and 7. Values represent the means and standard deviation (SD) from a duplicate (n=2). 5 mol equivalents of TCEP were added to free-thiol samples to ensure that no oxidized species were present.

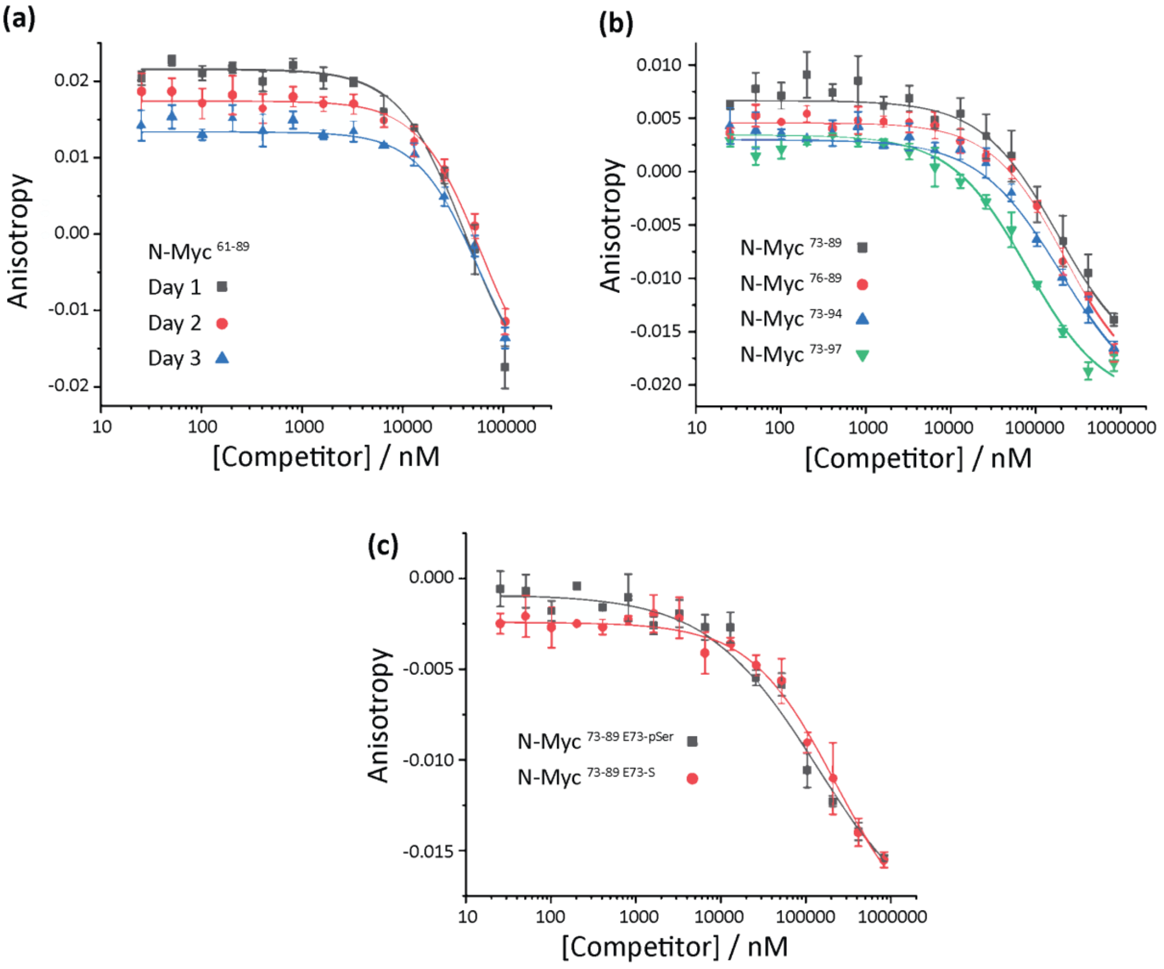

Figure S1. Competition FA results for peptides in Table 1. (25 mM Tris, 150 mM NaCl, 5 mM $MgCl_2$, pH 7.5, 15 µM Aurora-A $_{122\text{-}403\text{-}C290A/C393A}$, 50 nM FAM-Ahx-N-Myc $_{61\text{-}89}$, 25 °C).

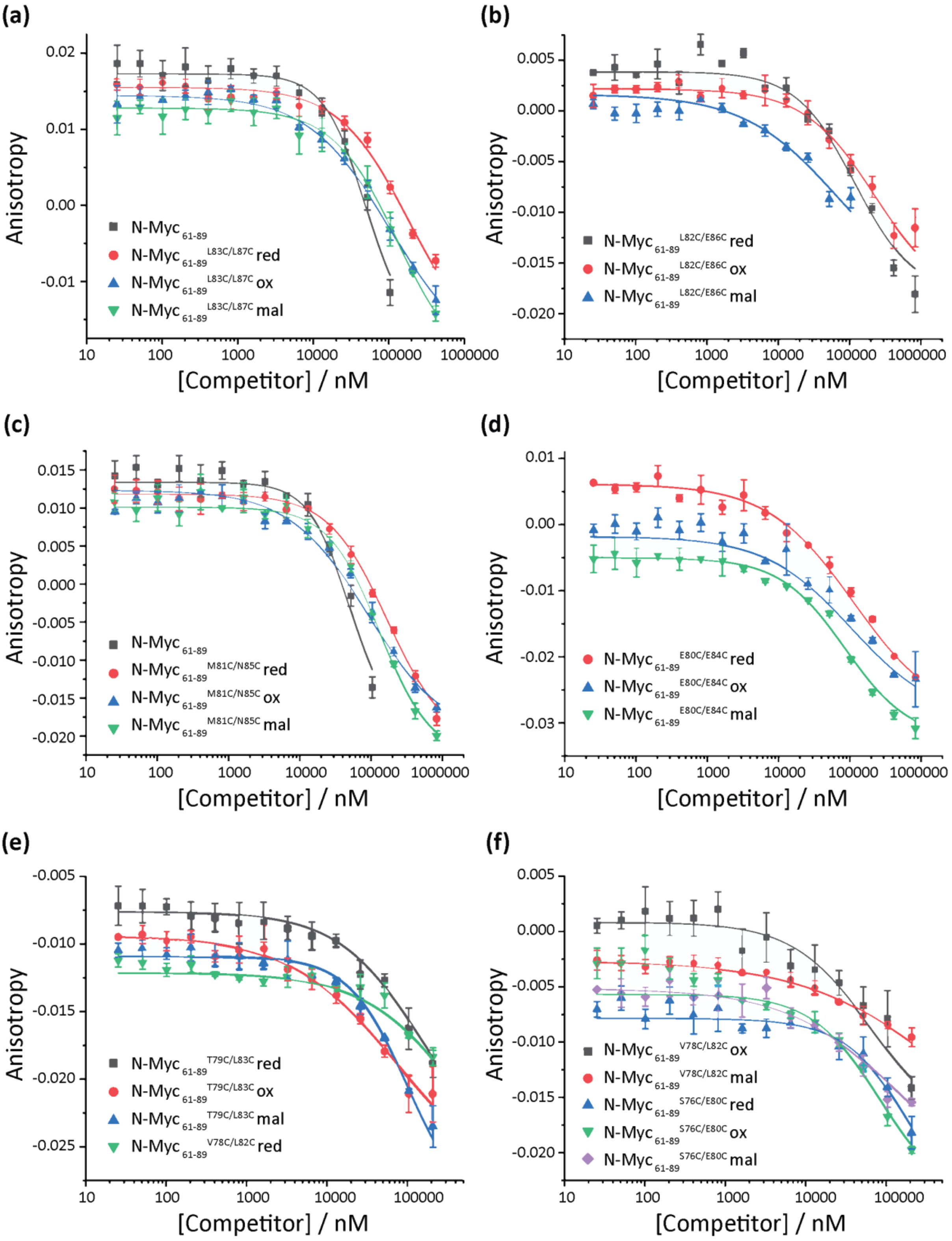

Figure S2. Competition FA results for peptides in Table S1. (25 mM Tris, 150 mM NaCl, 5 mM $MgCl_2$, pH 7.5, 15 µM Aurora-A $_{122\text{-}403\text{-}C290A/C393A}$, 50 nM FAM-Ahx-N-Myc $_{61\text{-}89}$, 25 °C).

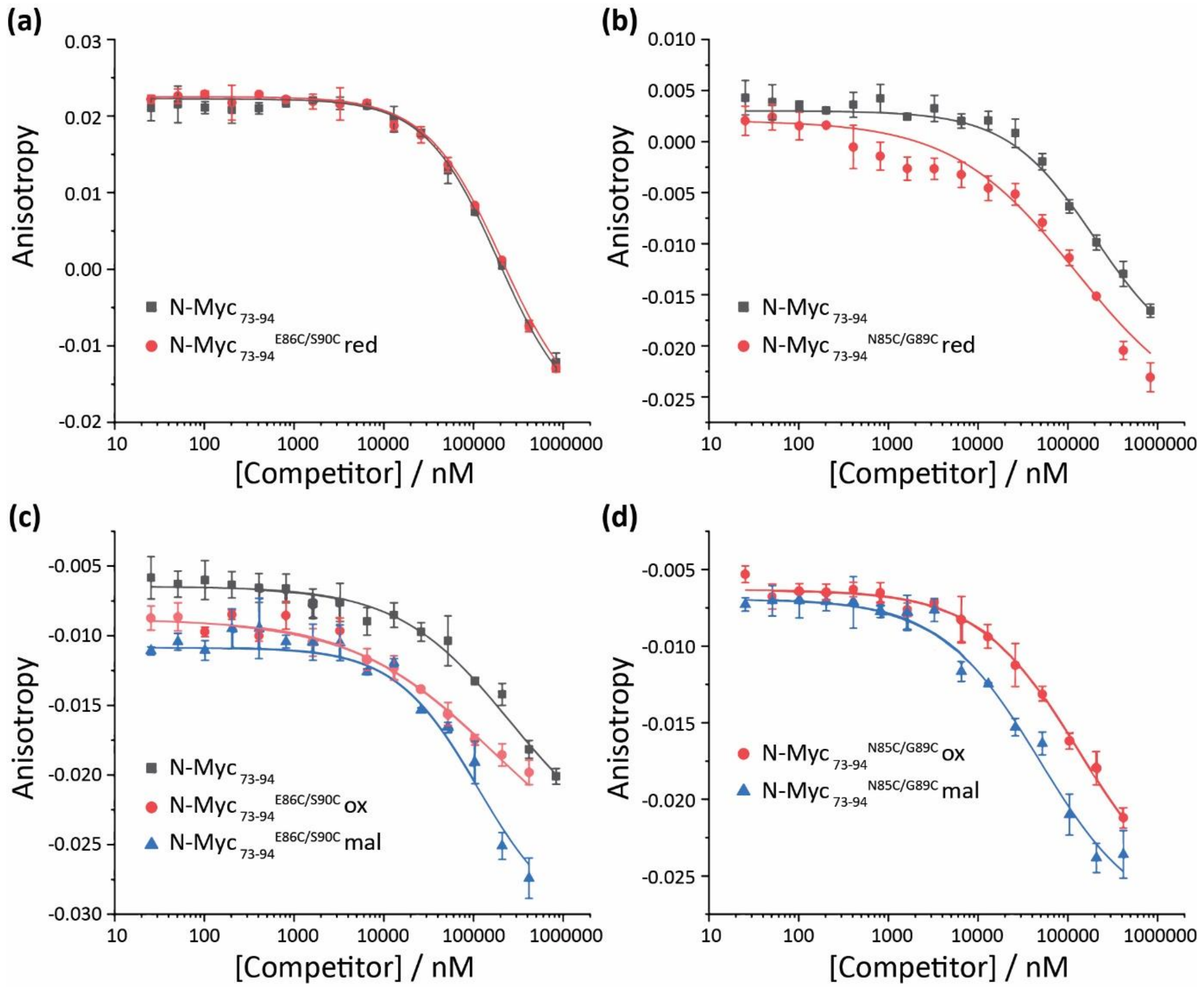

Figure S3. Competition FA results for peptides in Table 2. (25 mM Tris, 150 mM NaCl, 5 mM $MgCl_2$) pH 7.5, 15 µM Aurora-A $_{122\text{-}403\text{-}C290A/C393A}$, 50 nM FAM-Ahx-N-Myc $_{61\text{-}89}$, 25 °C).

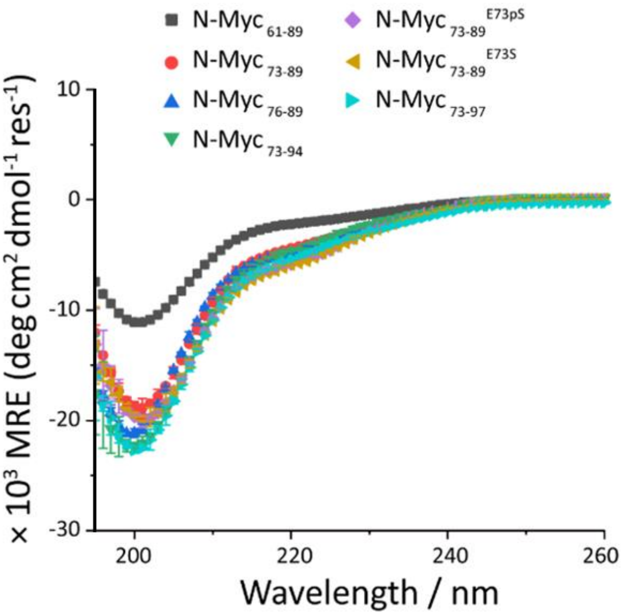

Figure S4. Averaged duplicate CD spectra of N-Myc variants in Table 1. (25 mM Tris, 150 mM NaCl, 5 mM $MgCl_2$, pH 7.5, 5 °C).

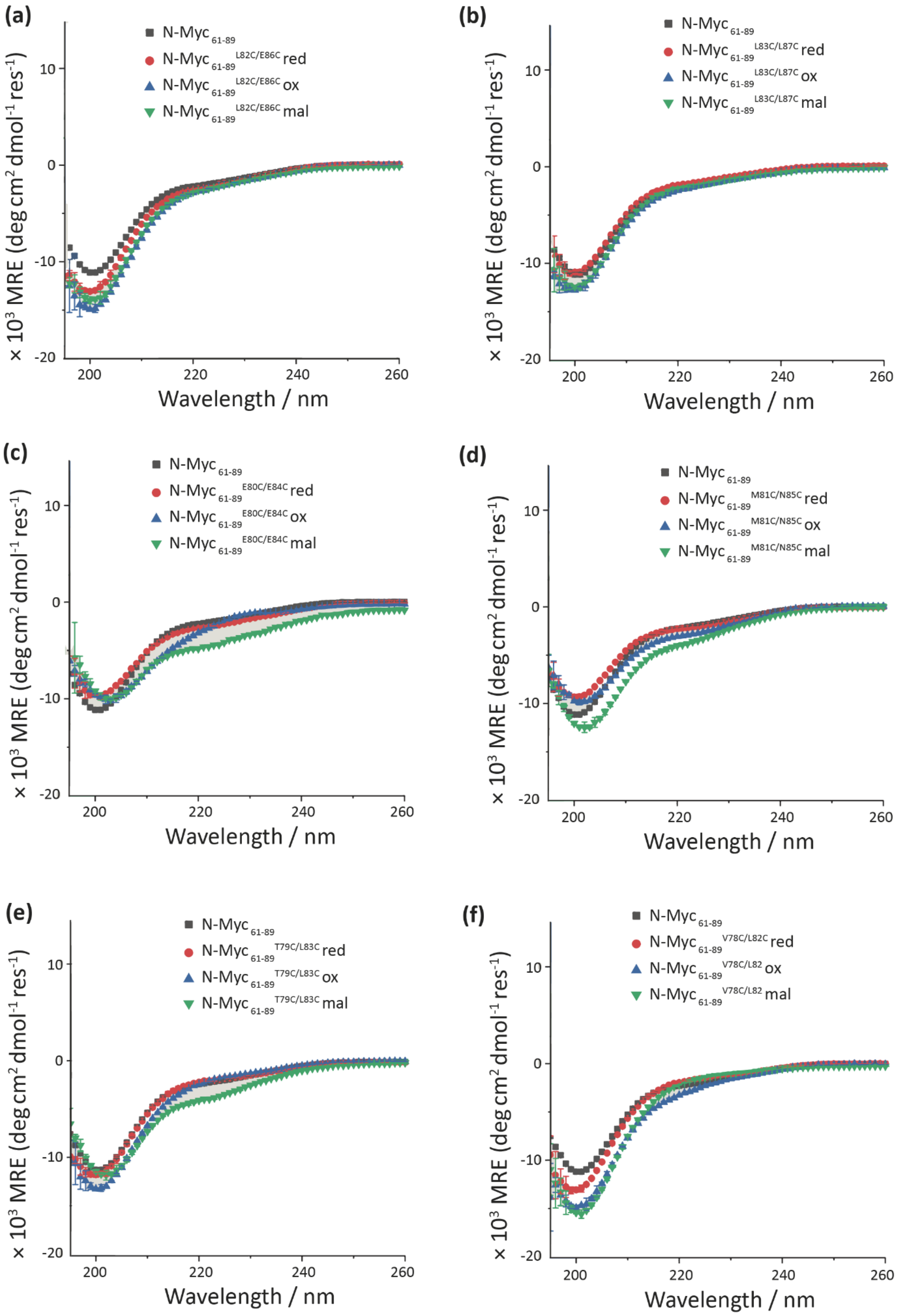

(a)
N-Myc 61-89
N-Myc 61-89 L82C/E86C red
N-Myc 61-89 L82C/E86C ox
N-Myc 61-89 L82C/E86C mal
(b)
N-Myc 61-89
N-Myc 61-89 L83C/L87C red
N-Myc 61-89 L83C/L87C ox
N-Myc 61-89 L83C/L87C mal
(c)
N-Myc 61-89
N-Myc 61-89 E80C/E84C red
N-Myc 61-89 E80C/E84C ox
N-Myc 61-89 E80C/E84C mal
(d)
N-Myc 61-89
N-Myc 61-89 M81C/N85C red
N-Myc 61-89 M81C/N85C ox
N-Myc 61-89 M81C/N85C mal
(e)
N-Myc 61-89
N-Myc 61-89 T79C/L83C red
N-Myc 61-89 T79C/L83C ox
N-Myc 61-89 T79C/L83C mal
(f)
N-Myc 61-89
N-Myc 61-89 V78C/L82C red
N-Myc 61-89 V78C/L82 ox
N-Myc 61-89 V78C/L82 mal
× 10³ MRE (deg cm² dmol⁻¹ res⁻¹)
Wavelength / nm

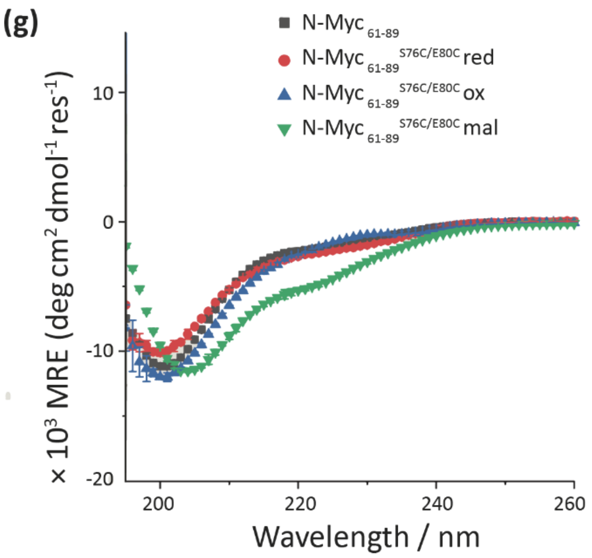

Figure S5. Averaged duplicate CD spectra of N-Myc variants in Table S1. (25 mM Tris, 150 mM NaCl, 5 mM $MgCl_2$, pH 7.5, 5 °C).

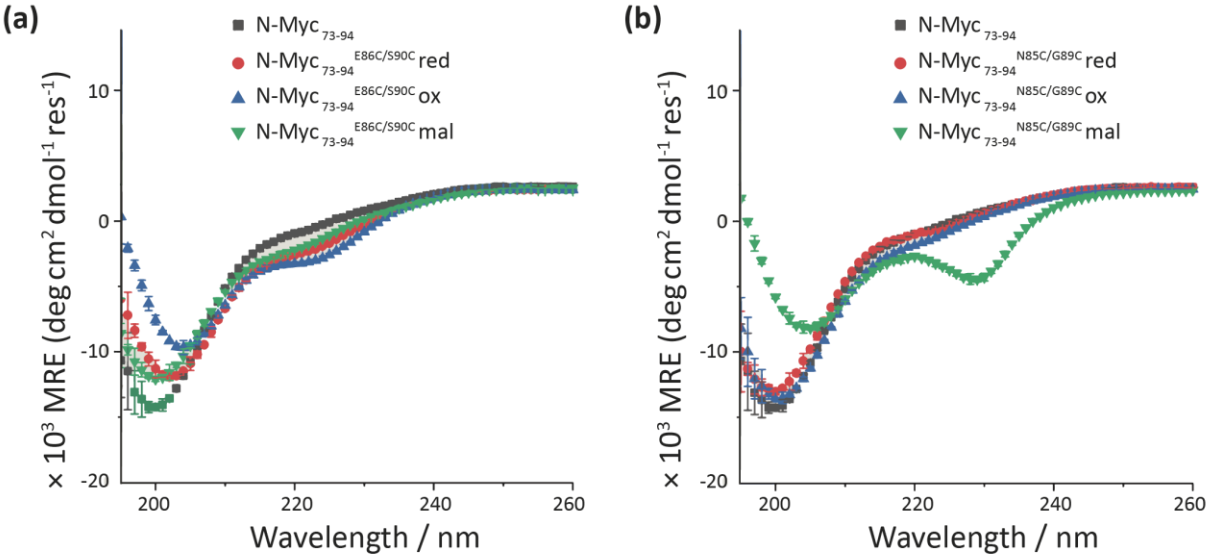

Figure S6. Averaged duplicate CD spectra of N-Myc variants in Table 2. (25 mM Tris, 150 mM NaCl, 5 mM $MgCl_2$, pH 7.5, 5 °C).

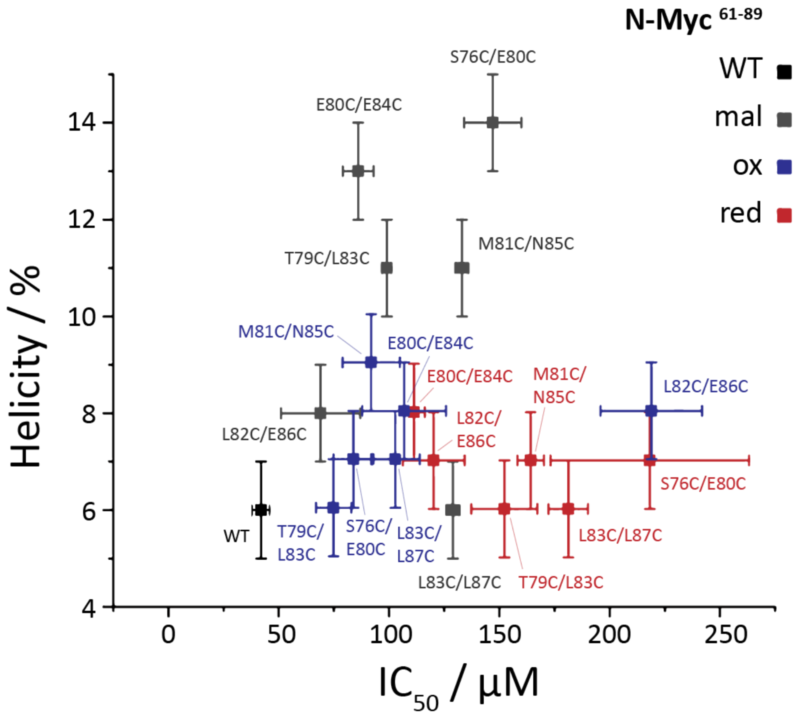

Figure S7. Plot of % helicity against IC_{50} for N-Myc $_{61\text{-}89}$ variants shown in Tables 1 and S2.

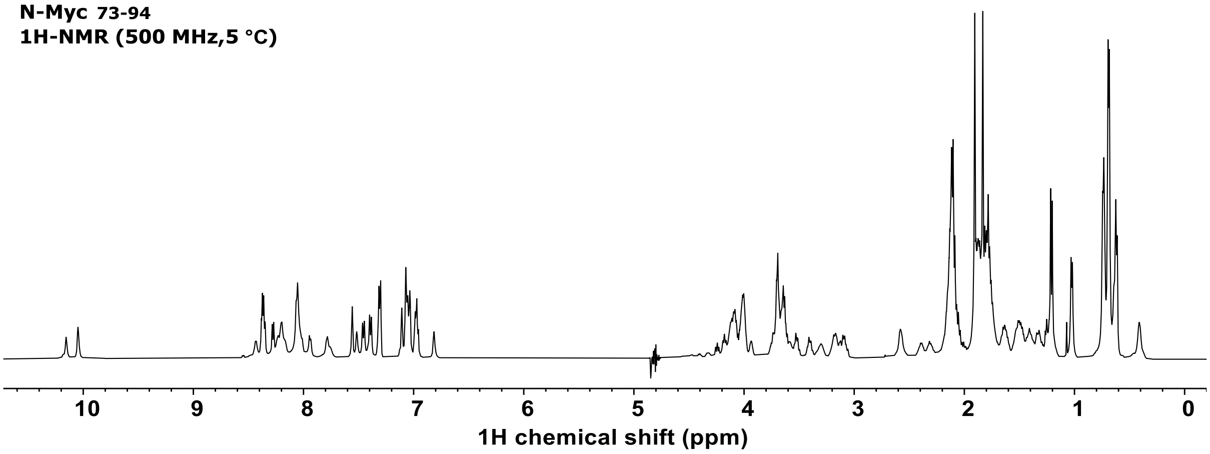

Figure S8. ^{1}H-NMR (500 MHz) trace of N-Myc $_{73\text{-}94}$ (buffer/D_2O 90/10 vol/vol at 5 °C. Buffer: 25 mM potassium phosphate, 50 mM NaCl, 5 mM $MgCl_2$, pH= 7.5). Note the presence of exchangeable tryptophan indole NH protons at 10-10.25 ppm.

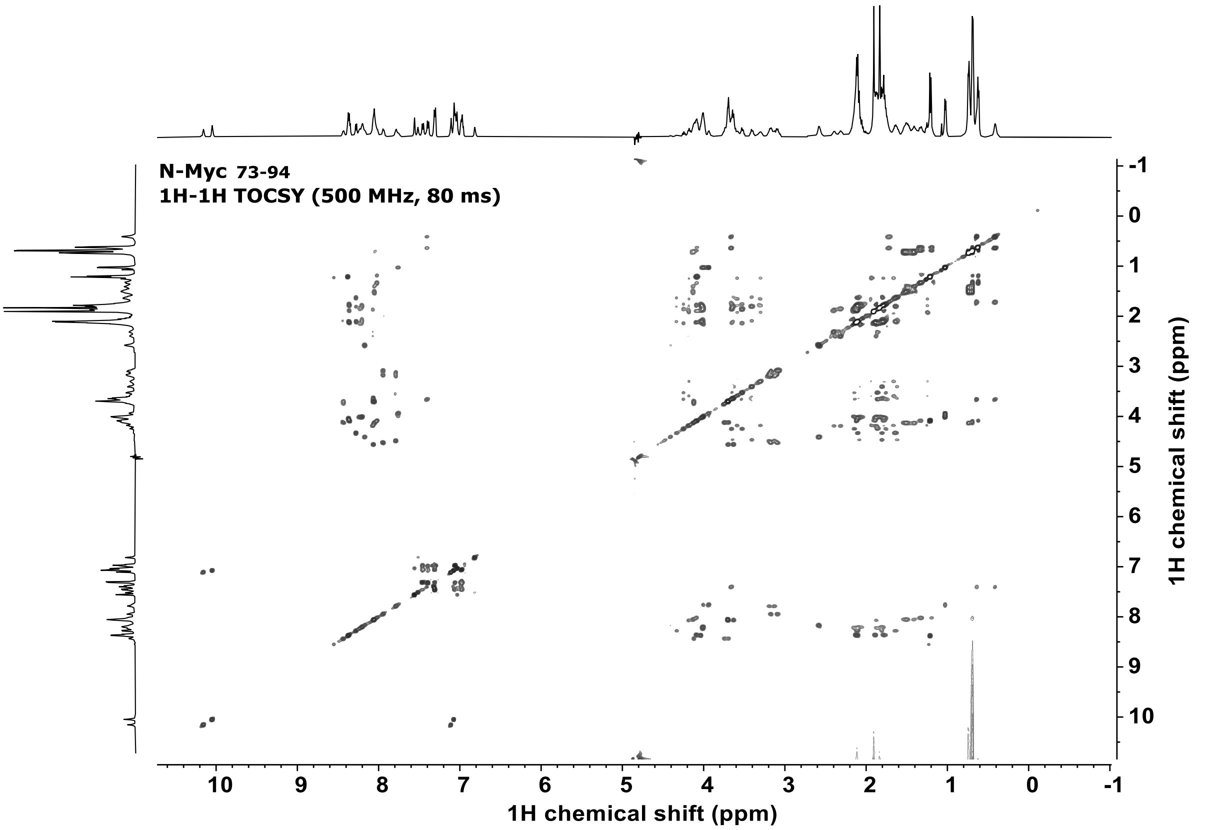

Figure S9. ^{1}H-^{1}H TOCSY NMR (500 MHz) trace of N-Myc $_{73\text{-}94}$ (buffer/D_2O 90/10 vol/vol at 5 °C. Buffer: 25 mM potassium phosphate, 50 mM NaCl, 5 mM $MgCl_2$, pH= 7.5).

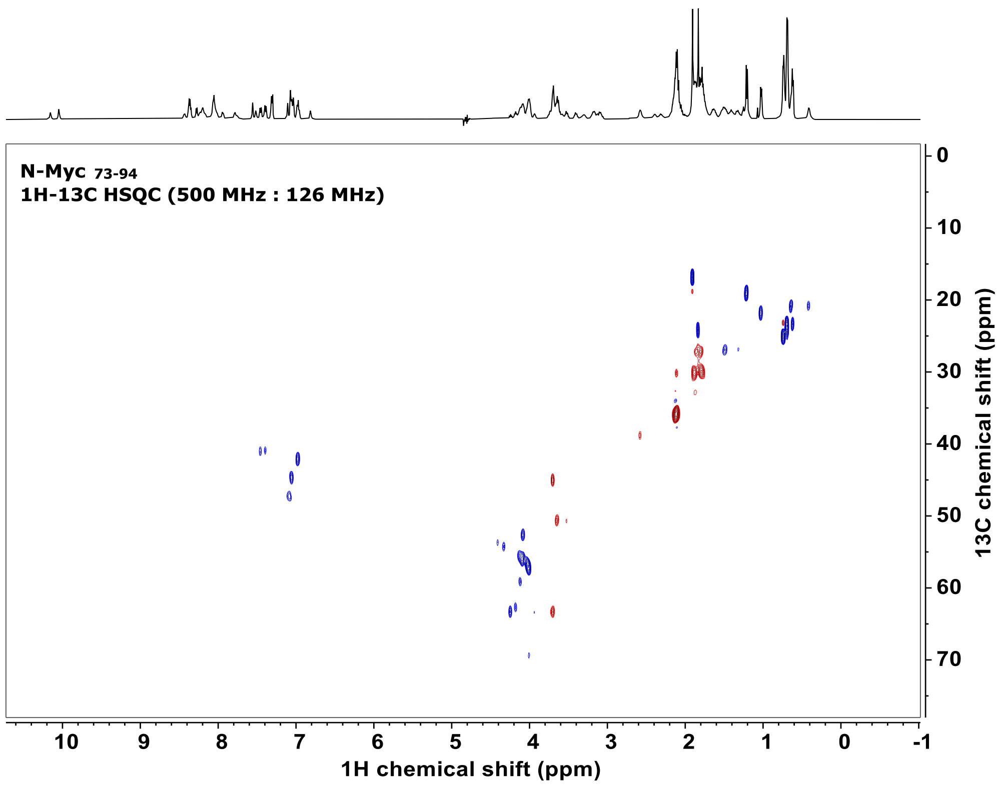

Figure S10. **^{1}H-^{13}C HSQC NMR (500:126 MHz) trace of N-Myc $_{73\text{-}94}$** (buffer/D_2O 90/10 vol/vol at 5 °C. Buffer: 25 mM potassium phosphate, 50 mM NaCl, 5 mM $MgCl_2$, pH= 7.5).

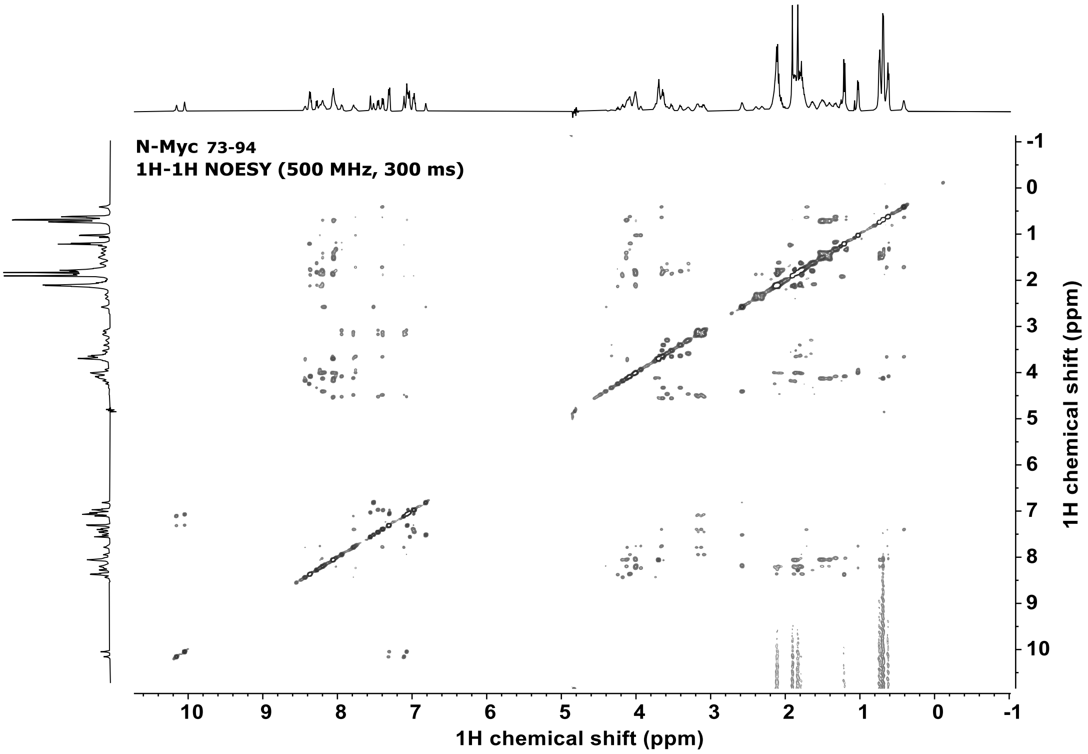

Figure S11. **^{1}H-^{1}H NOESY NMR (500 MHz) trace of N-Myc $_{73\text{-}94}$** (buffer/D_2O 90/10 vol/vol at 5 °C. Buffer: 25 mM potassium phosphate, 50 mM NaCl, 5 mM $MgCl_2$, pH= 7.5).

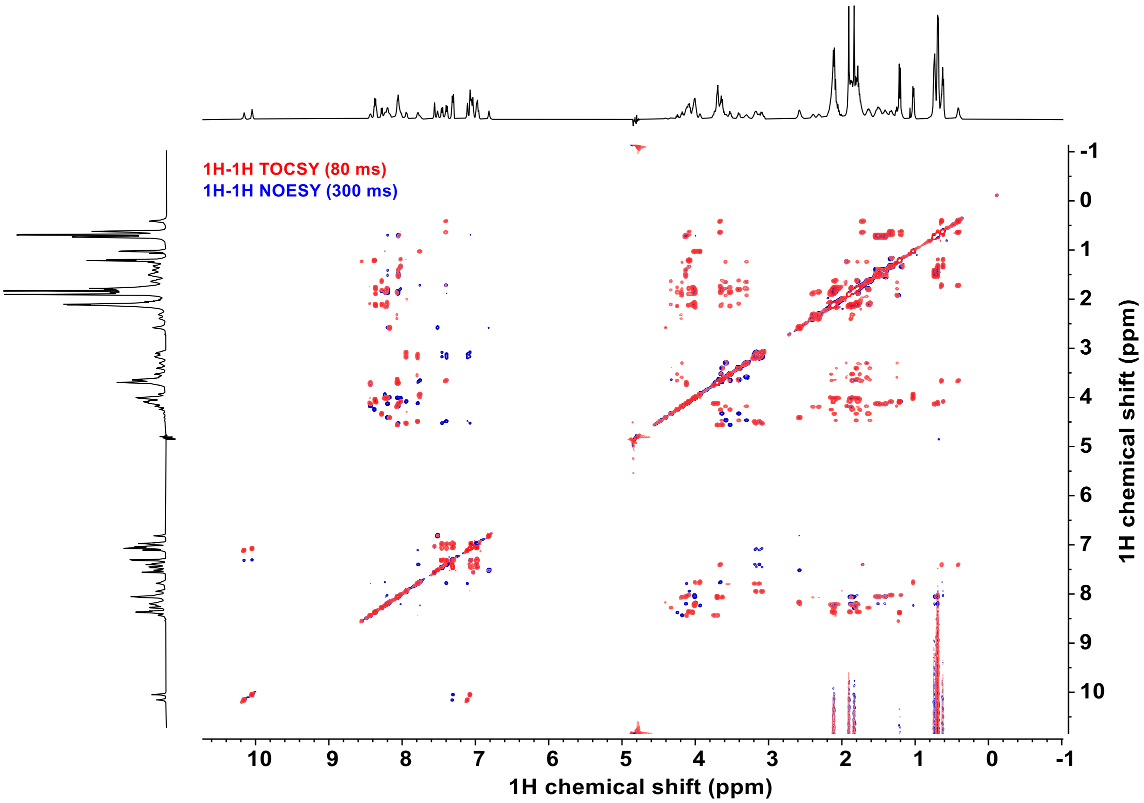

Figure S12. Overlaid ^{1}H-^{1}H TOCSY (in red) and ^{1}H-^{1}H NOESY spectra (in blue) of N-Myc $_{73\text{-}94}$ (buffer/D_2O 90/10 vol/vol at 5 °C. Buffer: 25 mM potassium phosphate, 50 mM NaCl, 5 mM $MgCl_2$, pH= 7.5).

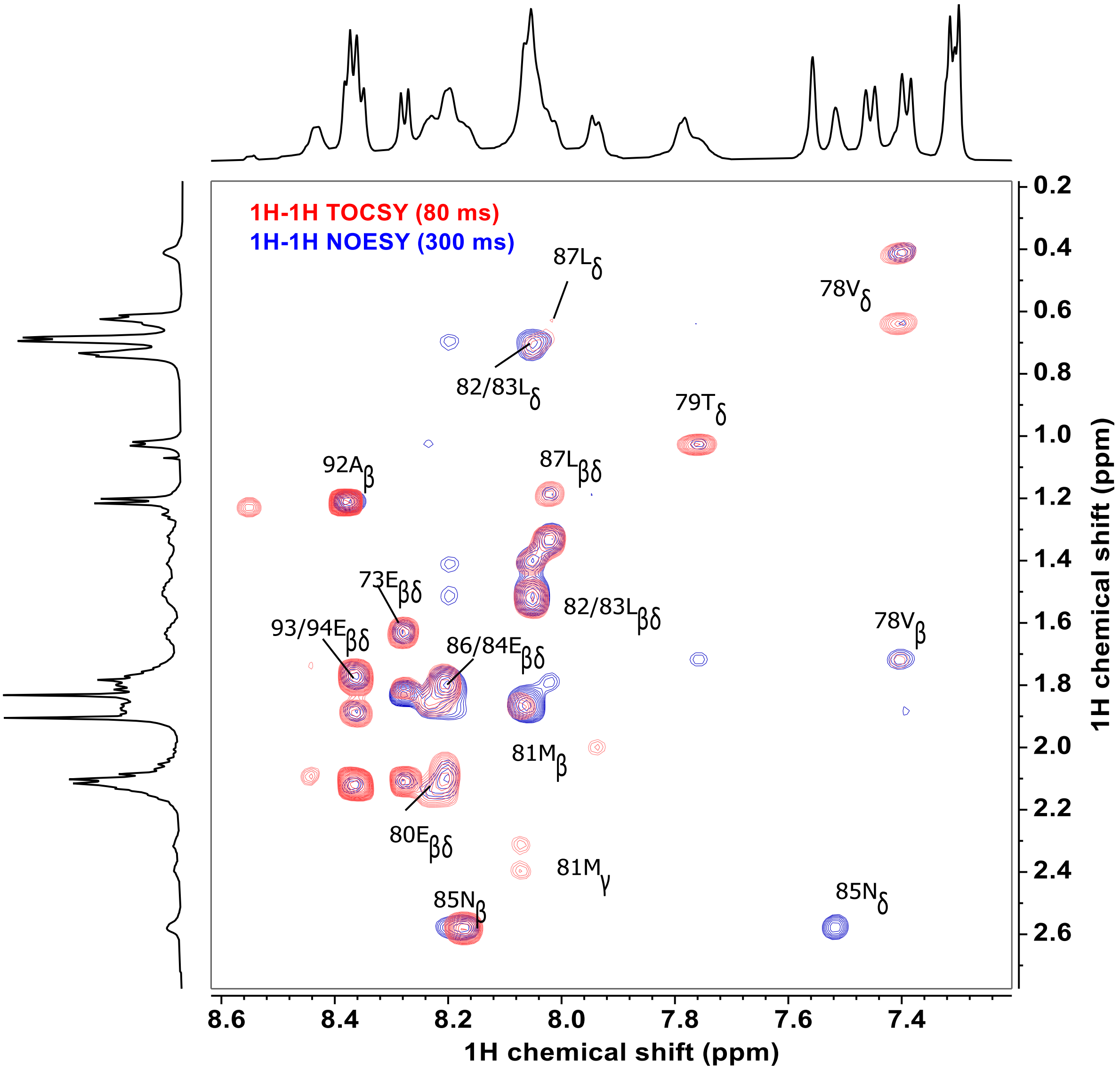

Figure S13. Magnified overlaid ^{1}H-^{1}H TOCSY spectra (red) and ^{1}H-^{1}H NOESY spectra (blue) of N-Myc $_{73-94}$ at the amide NH-side chain region showing ^{1}H signal assignments.

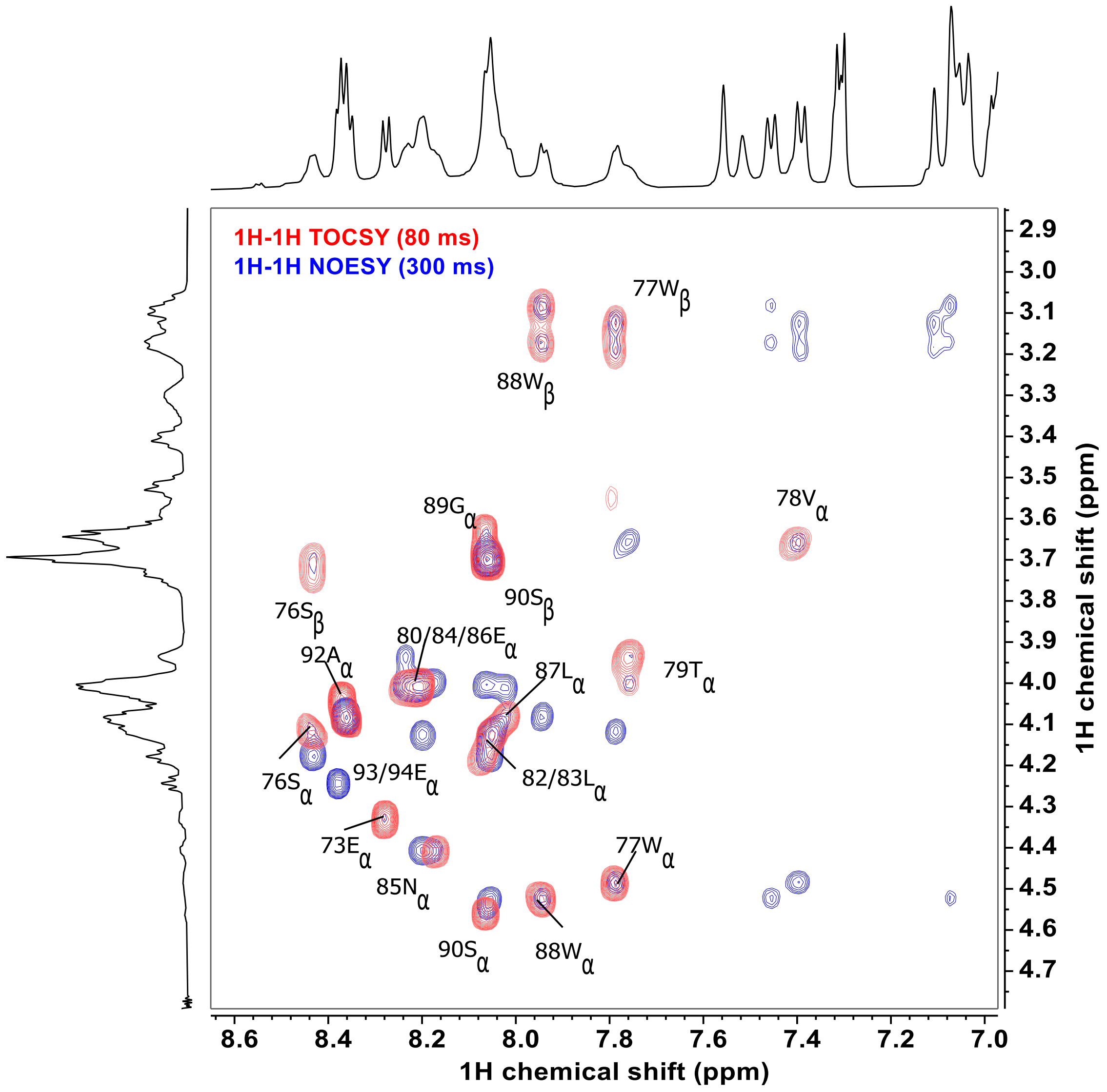

Figure S14. Magnified overlaid ^{1}H-^{1}H TOCSY spectra (red) and ^{1}H-^{1}H NOESY spectra (blue) of N-Myc $_{73\text{-}94}$ at the NH-Hα region showing ^{1}H signal assignments.

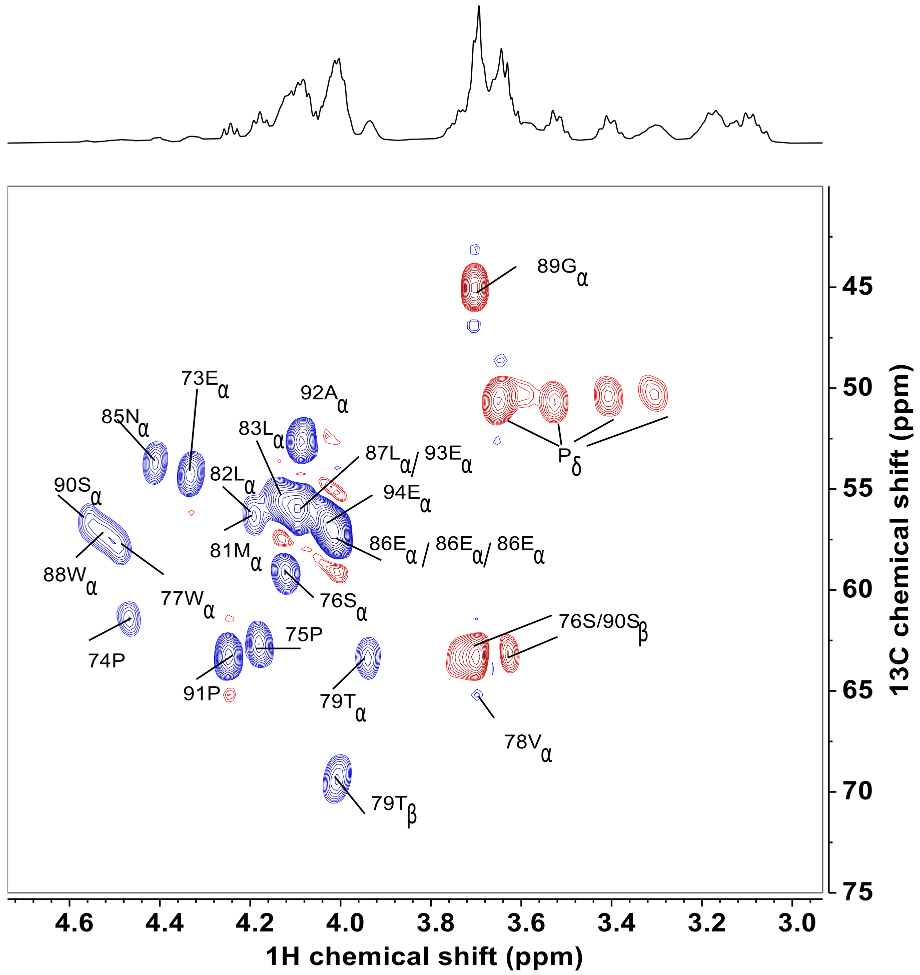

Figure S15. Magnified insert of the ^{1}H-^{13}C HSQC spectra of N-Myc $_{73\text{-}94}$ at the Cα region showing ^{13}C signal assignments.

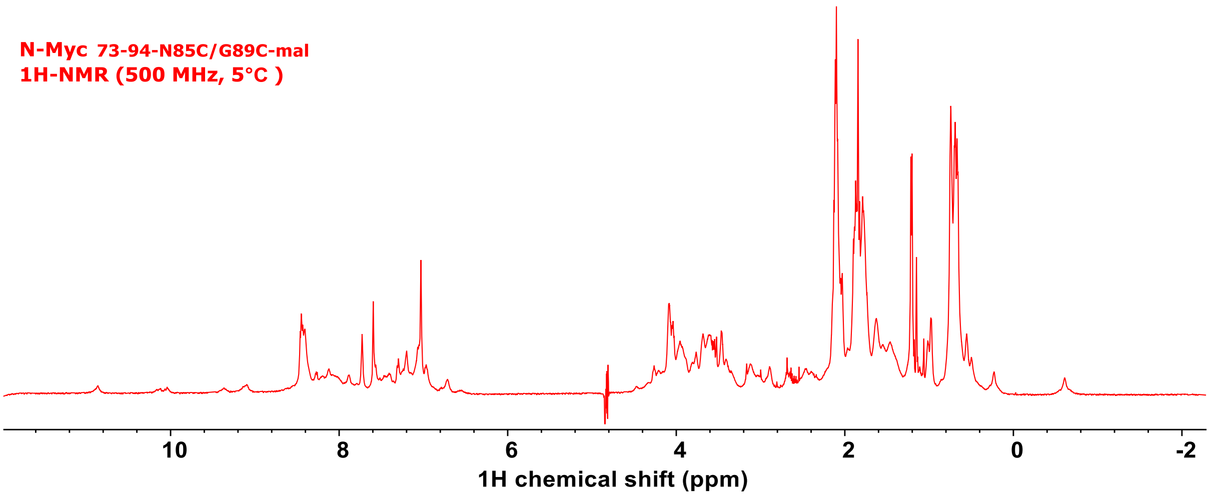

Figure S16. **^{1}H-NMR (500 MHz) trace of N-Myc $_{73\text{-}94\text{-}N85C/G89C\text{-}mal}$** (buffer/$D_2O$ 90/10 vol/vol at 5 °C. Buffer: 25 mM potassium phosphate, 50 mM NaCl, 5 mM $MgCl_2$, pH= 7.5). Note the presence of exchangeable tryptophan indole NH protons at 10-10.4 ppm, maleimide NH proton at 10.9 ppm and presence of additional unassigned exchangeable NH/OH protons at 9.0-9.75 ppm.

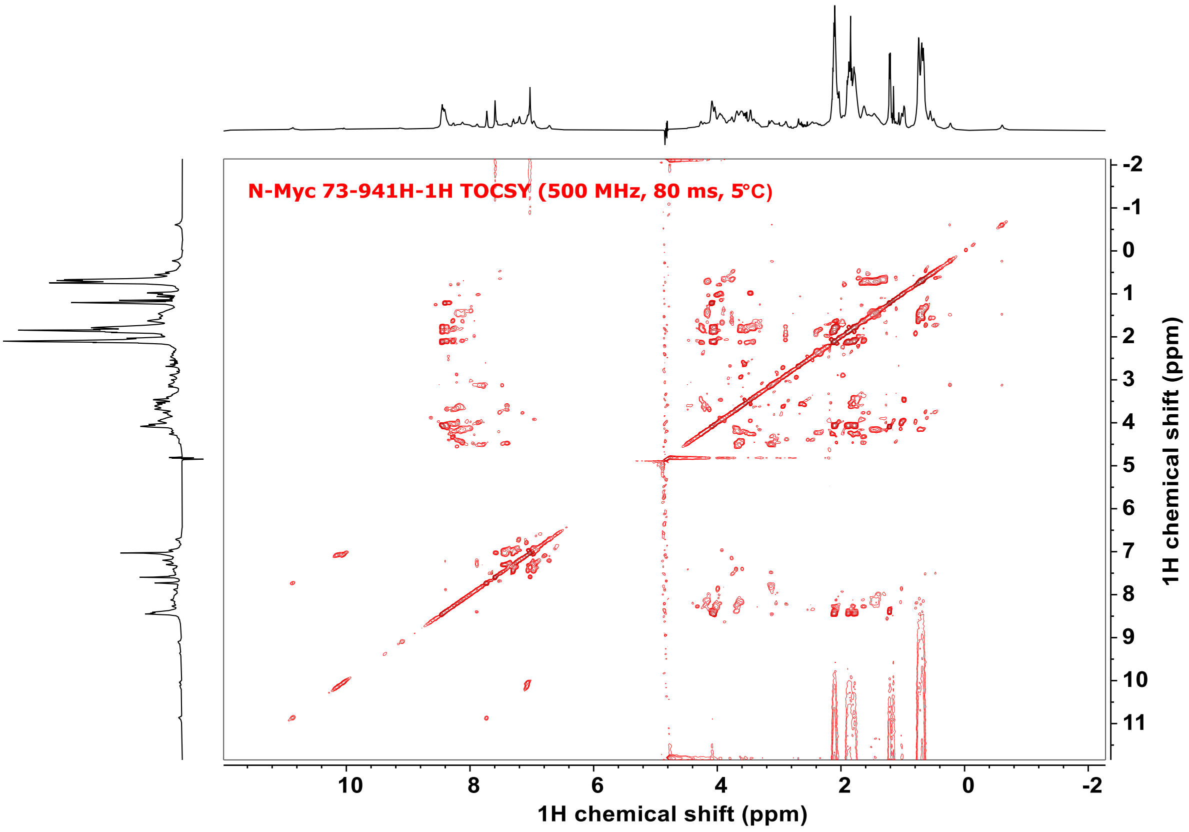

Figure S17. **^{1}H-^{1}H TOCSY NMR (500 MHz) spectra of N-Myc $_{73\text{-}94\text{-}N85C/G89C\text{-}mal}$** (buffer/$D_2O$ 90/10 vol/vol at 5 °C. Buffer: 25 mM potassium phosphate, 50 mM NaCl, 5 mM $MgCl_2$, pH= 7.5).

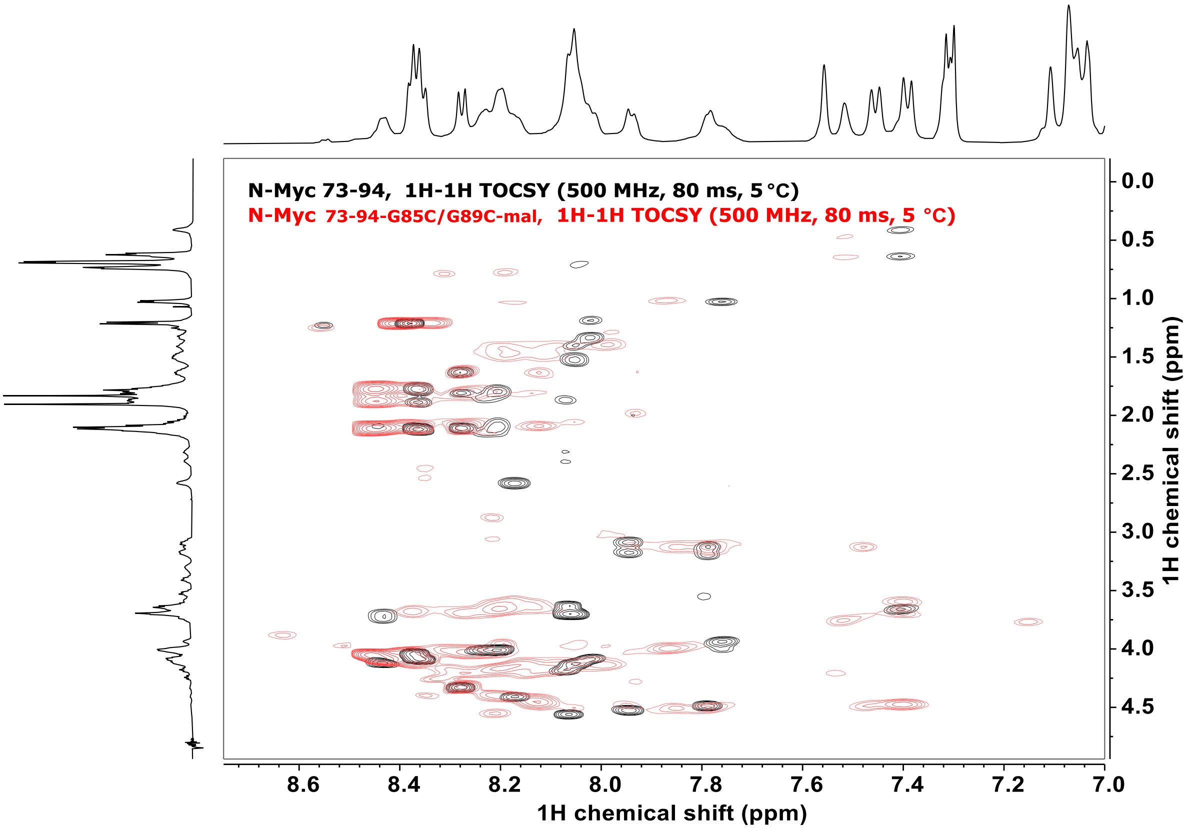

Figure S18. Overlaid ^{1}H-^{1}H TOCSY NMR (500 MHz) spectra of N-Myc $_{73-94}$ (in black) and N-Myc $_{73-94-N85C/G89C-mal}$ (red) showing broadly averaged signals for the constraint peptide at the NH-Hα region. All samples in buffer/D_2O 90/10 vol/vol at 5 °C. Buffer: 25 mM potassium phosphate, 50 mM NaCl, 5 mM $MgCl_2$, pH= 7.5.

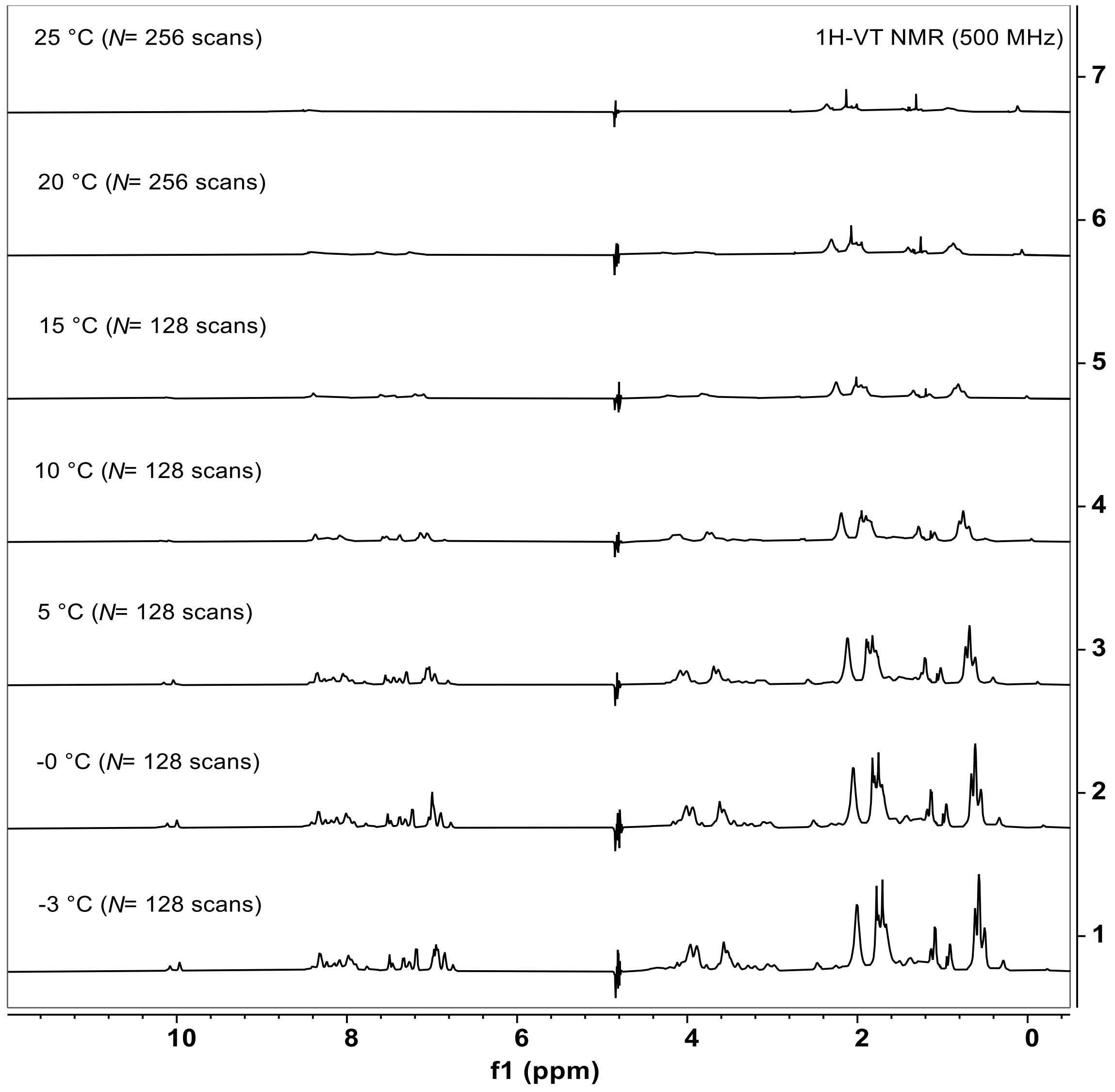

Figure S19. ^{1}H-VT NMR (500 MHz) traces observed for N-Myc $_{73-94}$ at increasing temperatures from -3 - 25 °C. Sample in 25 mM Tris, 150 mM NaCl, 5 mM $MgCl_2$, pH 7.5/ D_2O 90/10 v/v.

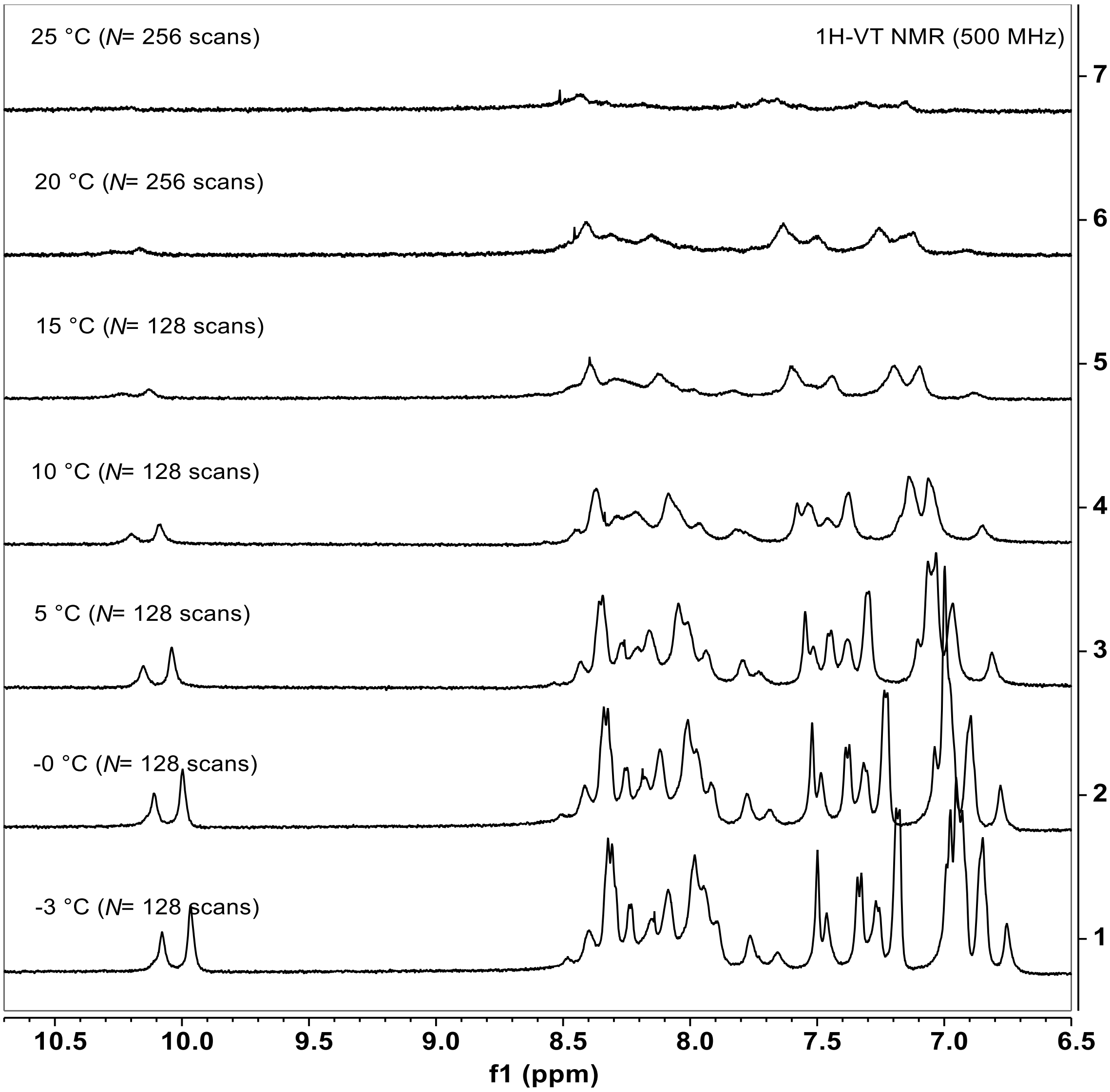

Figure S20. Inset of the ^{1}H-VT NMR (500 MHz) traces observed for N-Myc $_{73\text{-}94}$ at the amide NH region. Sample in 25 mM Tris, 150 mM NaCl, 5 mM $MgCl_2$, pH 7.5/ D_2O 90/10 v/v.

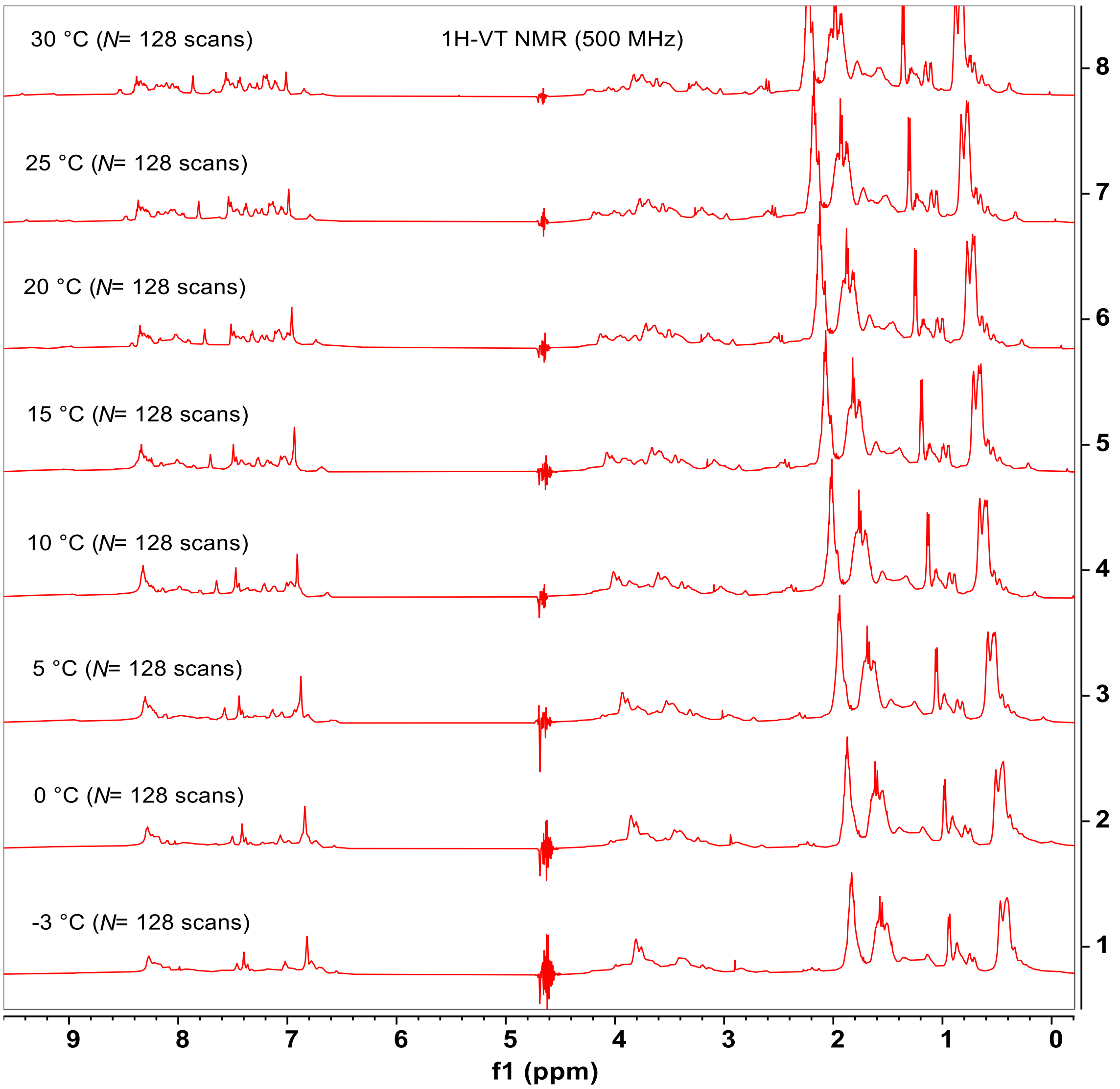

Figure S21. ^{1}H-VT NMR (500 MHz) traces observed for N-Myc $_{73\text{-}94\text{-}N85C/G89C\text{-}mal}$ at increasing temperatures from -3 - 30 °C. Sample in 25 mM Tris, 150 mM NaCl, 5 mM $MgCl_2$, pH 7.5/ D_2O 90/10 v/v.

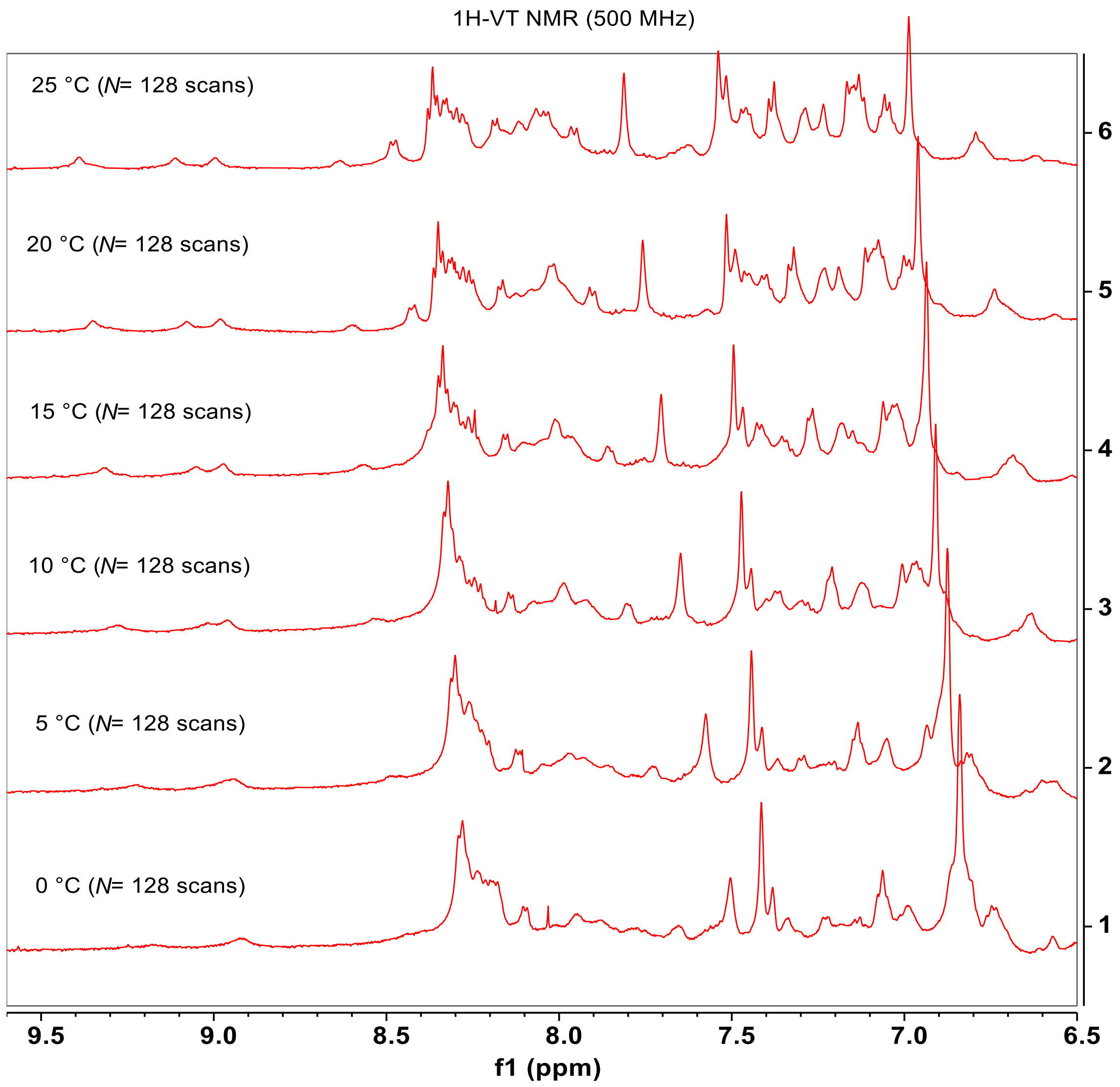

Figure S22. Inset showing the ^{1}H-VT NMR (500 mHz) traces observed for N-Myc$_{73\text{-}94\text{-}N85C/G89C\text{-}mal}$ at the amide NH region. Sample in 25 mM Tris, 150 mM NaCl, 5 mM $MgCl_2$, pH 7.5/ D_2O 90/10 v/v.

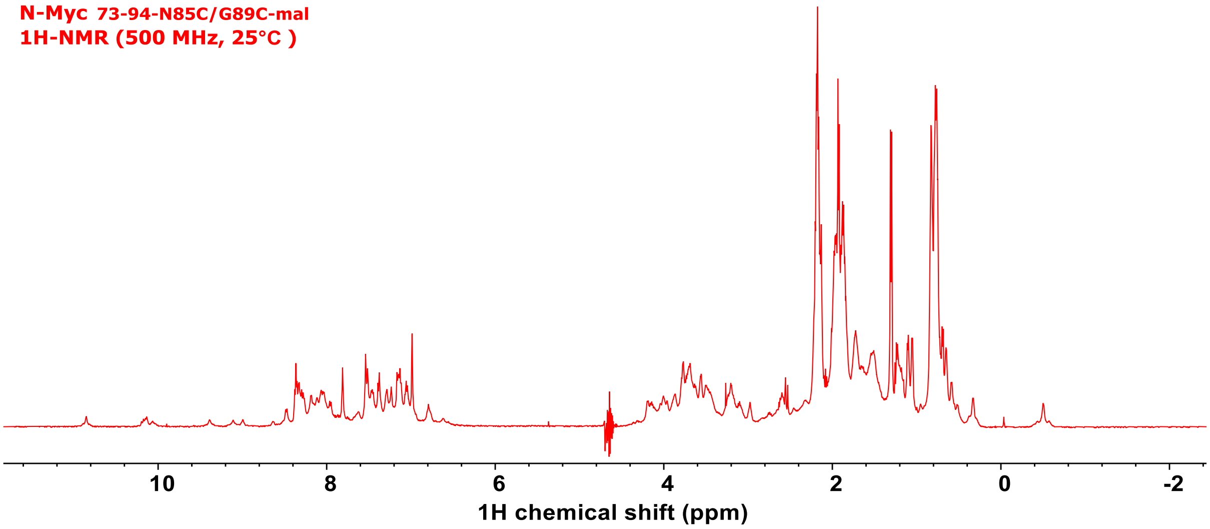

Figure S23. ^{1}H-NMR (500 MHz) trace of N-Myc$_{\text{73-94-N85C/G89C-mal}}$ (buffer/D_2O 90/10 vol/vol at 25 °C. Buffer: 25 mM potassium phosphate, 50 mM NaCl, 5 mM $MgCl_2$, pH= 7.5). Note the presence of exchangeable tryptophan indole NH protons at 10-10.5 ppm, maleimide NH proton at 10.9 ppm and presence of additional unassigned exchangeable NH/OH protons at 9.0-9.75 ppm.

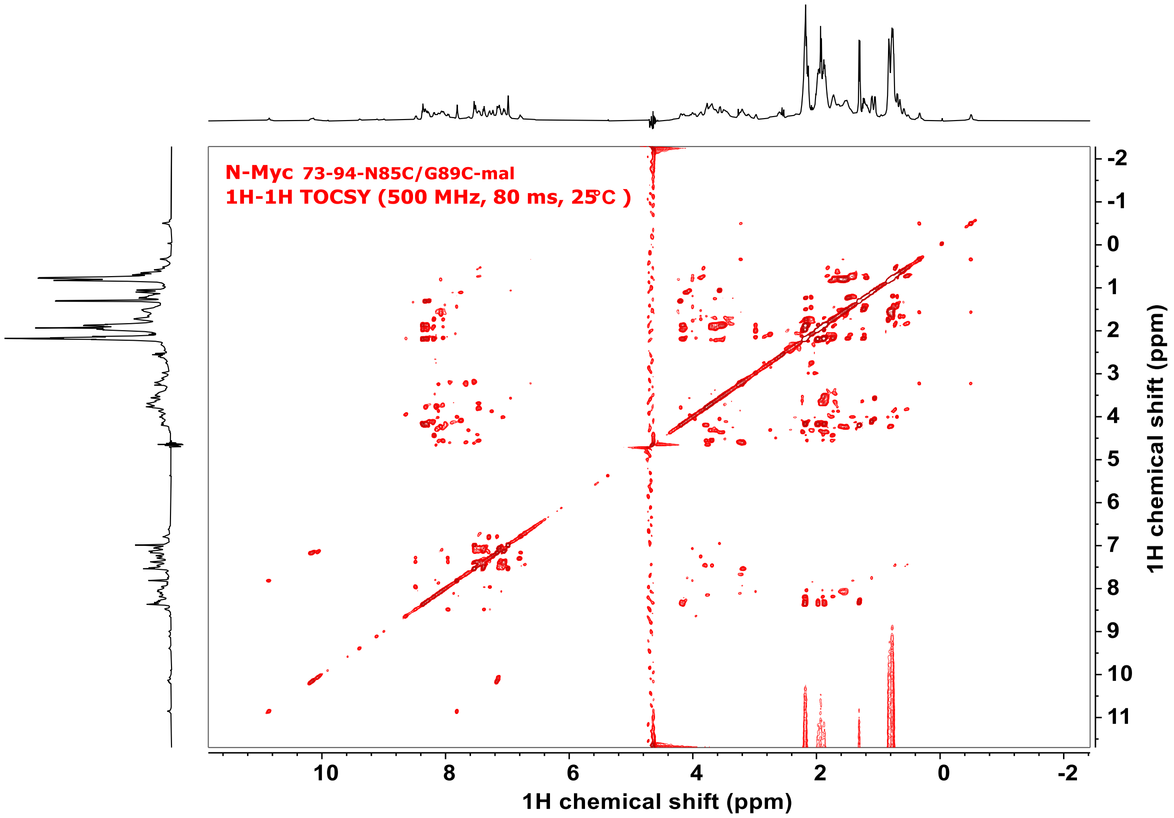

Figure S24. ^{1}H-^{1}H TOCSY NMR (500 MHz) trace of N-Myc$_{\text{73-94-N85C/G89C-mal}}$ (buffer/D_2O 90/10 vol/vol at 25 °C. Buffer: 25 mM potassium phosphate, 50 mM NaCl, 5 mM $MgCl_2$, pH= 7.5).

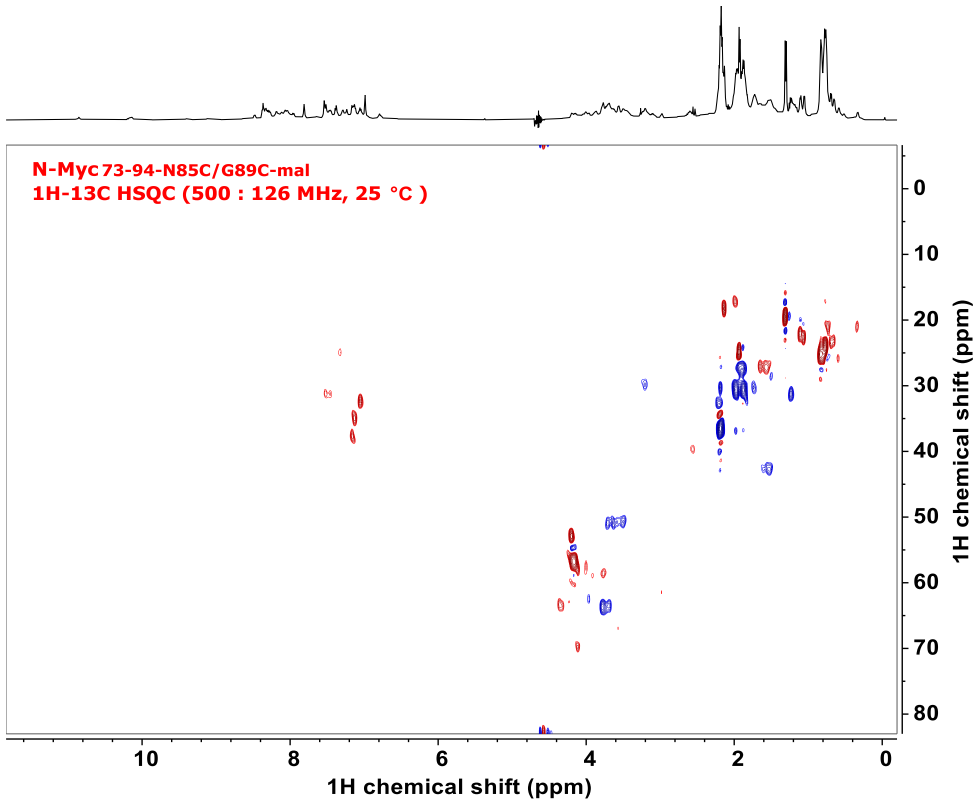

Figure S25. ^{1}H-^{13}C HSQC NMR (500:126 MHz) trace of N-Myc$_{73-94-N85C/G89C-mal}$ (buffer/D_2O 90/10 vol/vol at 25 °C. Buffer: 25 mM potassium phosphate, 50 mM NaCl, 5 mM $MgCl_2$, pH= 7.5).

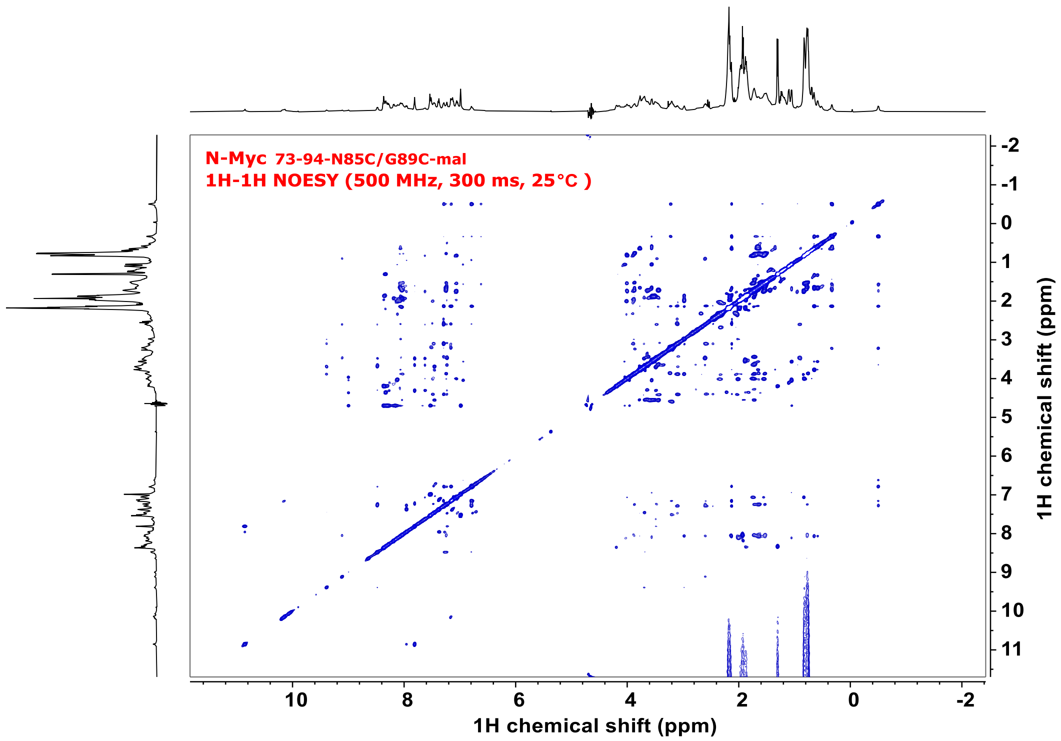

Figure S26. ^{1}H-^{1}H NOESY NMR (500 MHz) trace of N-Myc$_{73-94-N85C/G89C-mal}$ (buffer/D_2O 90/10 vol/vol at 25 °C).

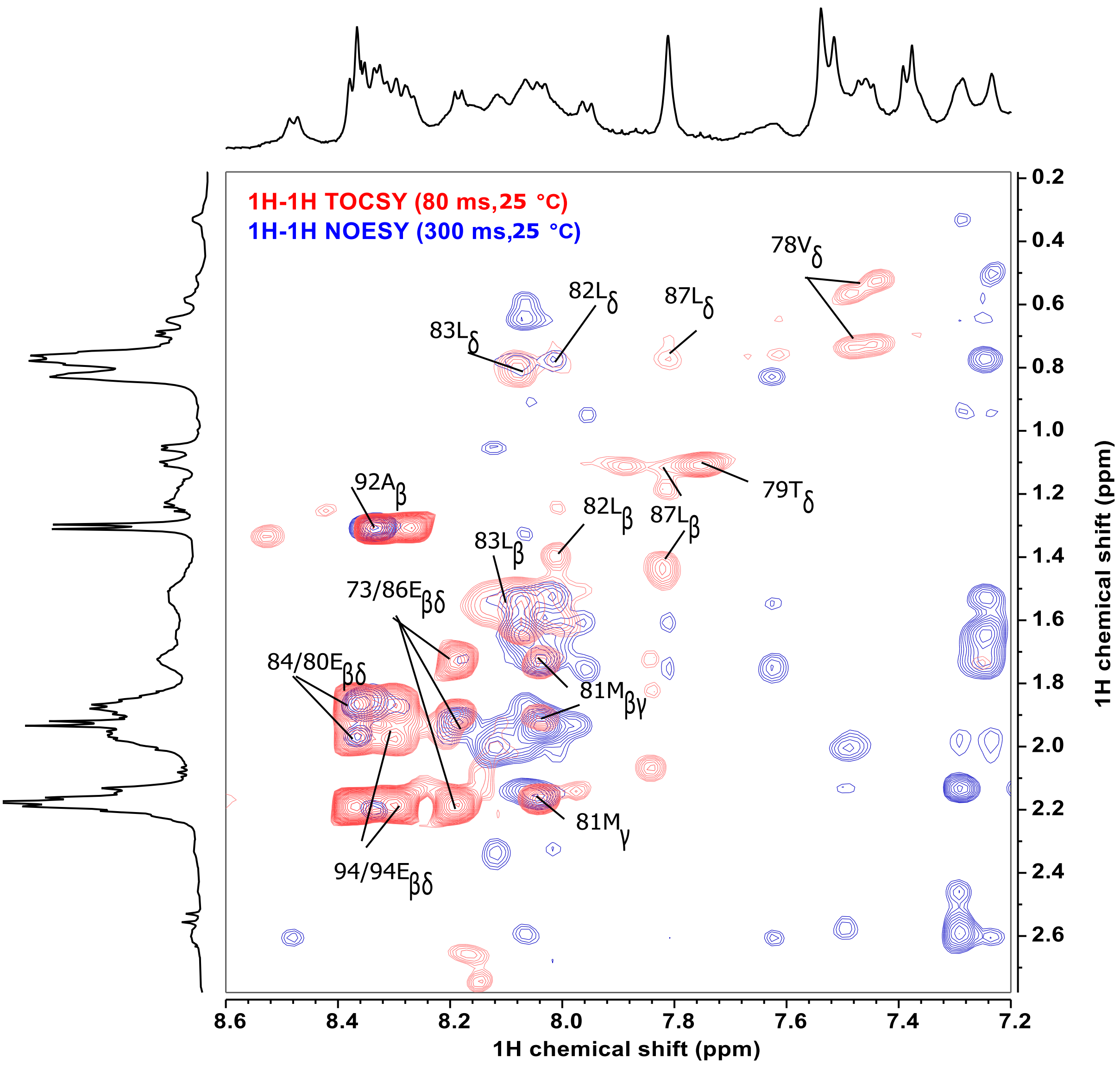

Figure S27. Magnified ^{1}H-^{1}H TOCSY spectra (red) and ^{1}H-^{1}H NOESY spectra (blue) of N-Myc $_{73\text{-}94\text{-}N85C/G89C\text{-}mal}$ at the amide NH-side chain region showing sequence signal assignments.

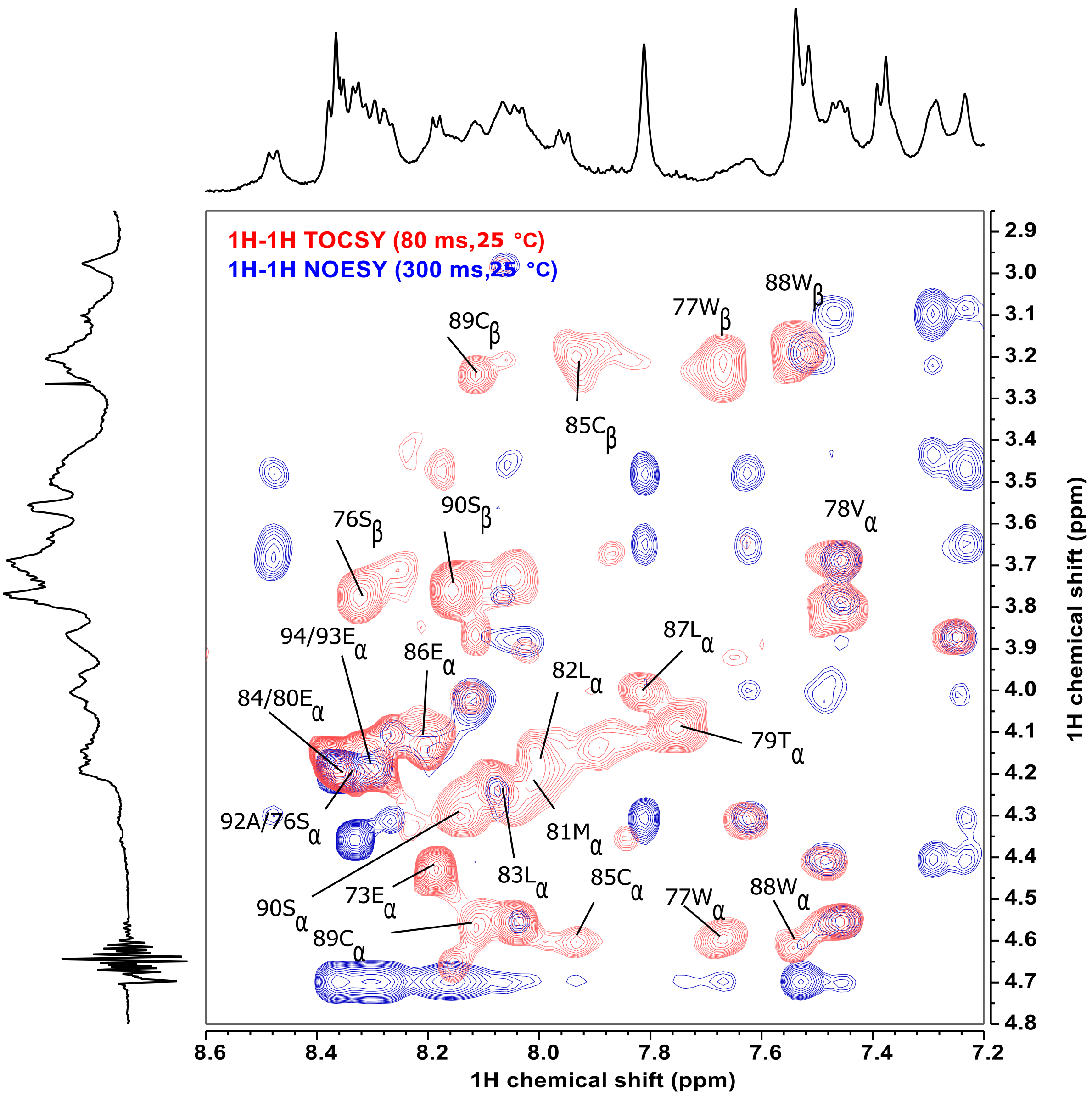

Figure S28. Magnified ^{1}H-^{1}H TOCSY spectra (red) and ^{1}H-^{1}H NOESY spectra (blue) of N-Myc$_{73\text{-}94\text{-}N85C/G89C\text{-}mal}$ at the amide NH-Hα region showing ^{1}H signal assignments.

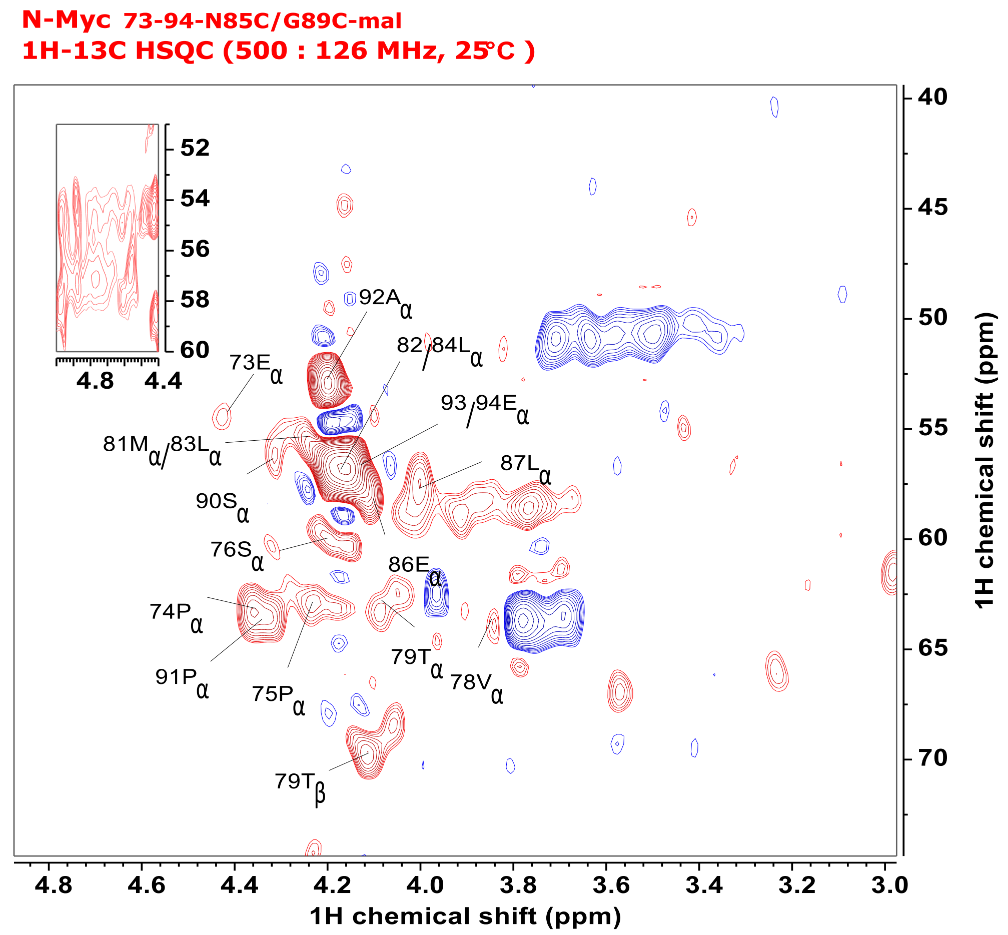

Figure S29. Magnified insert of the ^{1}H-^{13}C HSQC spectra of N-Myc $_{73\text{-}94\text{-}N85C/G89C\text{-}mal}$ at the Cα region showing ^{13}C signal assignments.

N-Myc 73-94 Residue[a]	$\delta_{C\alpha,RC}$[b]	$\delta_{C\alpha}$, obs[c] [d]	$\Delta\delta_{C\alpha}$[e]	$\delta_{H\alpha,RC}$ [b]	$\delta_{H\alpha}$, obs[c] [d]	$\Delta\delta_{H\alpha}$[e]	$\delta_{HN,RC}$ [b]	δ_{HN}, obs[c] [d]	$\Delta\delta_{HN}$[e]
E73	54.354	54.26 ± 0.19	-0.094	4.579	4.329 ± 0.005	-0.25	8.659	8.279 ± 0.002	-0.38
P74	60.954	61.52 ± 0.24	0.566	4.746	4.467 ± 0.003	-0.279			
P75	63.144	62.77 ± 0.34	-0.374	4.397	4.158 ± 0.011	-0.239			
S76	58.653	59.31 ± 0.24	0.657	4.345	4.121 ± 0.008	-0.224	8.582	8.436 ± 0.003	-0.146
W77	57.216	57.77 ± 0.22	0.554	4.773	4.487 ± 0.017	-0.286	8.185	7.789 ± 0.003	-0.396
V78	62.309	64.02 ± 0.24	1.711	4.036	3.659 ± 0.021	-0.377	7.809	7.404 ± 0.004	-0.405
T79	62.042	63.34 ± 0.28	1.298	4.191	3.949 ± 0.025	-0.242	8.076	7.767 ± 0.008	-0.309
E80	56.734	56.86 ± 0.24	0.126	4.273	4.008 ± 0.013	-0.265	8.535	8.235 ± 0.003	-0.3
M81	55.458	56.33 ± 0.31	0.872	4.463	4.179 ± 0.039	-0.284	8.566	8.068 ± 0.004	-0.498
L82	55.097	56.33 ± 0.31	1.233	4.372	4.134 ± 0.012	-0.238	8.406	8.051 ± 0.003	-0.355
L83	55.22	55.61 ± 0.22	0.39	4.358	4.134 ± 0.012	-0.224	8.363	8.051 ± 0.003	-0.312
E84	56.661	56.86 ± 0.24	0.199	4.299	4.006 ± 0.008	-0.293	8.559	8.197 ± 0.004	-0.362
N85	53.426	53.73 ± 0.19	0.304	4.687	4.412 ± 0.007	-0.275	8.566	8.171 ± 0.004	-0.395
E86	56.765	56.86 ± 0.24	0.095	4.199	4.012 ± 0.012	-0.187	8.61	8.207 ± 0.005	-0.403
L87	55.604	55.7 ± 0.40	0.096	4.277	4.098 ± 0.021	-0.179	8.353	8.025 ± 0.006	-0.328
W88	57.467	57.24 ± 0.27	-0.227	4.717	4.52 ± 0.016	-0.197	8.178	7.944 ± 0.003	-0.234
G89	45.102	45.09 ± 0.20	-0.012	3.912	3.722 ± 0.005	-0.19	7.993	8.061 ± 0.001	0.068
S90	56.467	56.67 ± 0.20	0.203	4.588	4.583 ± 0.005	-0.005	8.012	8.0633 ± 0.001	0.0513
P91	63.141	63.3 ± 0.29	0.159	4.491	4.247 ± 0.005	-0.244			
A92	52.71	52.68 ± 0.27	-0.03	4.278	4.044 ± 0.001	-0.234	8.51	8.376 ± 0.002	-0.134
E93	56.696	55.7 ± 0.40	-0.996	4.261	4.061 ± 0.015	-0.2	8.547	8.365 ± 0.007	-0.182
E94	56.78	56.62 ± 0.29	-0.16	4.258	4.061 ± 0.015	-0.197	8.597	8.365 ± 0.007	-0.232

Table S2. Secondary chemical shifts in N-Myc 73-94. [a] One letter code for amino acids. [b] Theoretical random coil $\delta_{C\alpha,RC}$, $\delta_{H\alpha,RC}$ and $\delta_{NH,RC}$ chemical shifts according to https://www1.bio.ku.dk/english/research/bms/sbinlab/randomchemicalshifts2/,[1–3] as calculated for N-Myc 73-94 at **5 °C** and pH= 7.5. [c] Observed experimental Cα, Hα and amide HN chemical shifts (ppm). [d] Error represents the uncertainly in peak centre determination (peak broad) as observed at the 1.2 contour level. [e]Calculated secondary (δ_{RC} - δ_{obs}) $\Delta\delta_{C\alpha}$, $\Delta\delta_{H\alpha}$ and $\Delta\delta_{NH}$ chemical shifts (ppm).

N-Myc $_{73\text{-}94\text{-}N85C/G89C\text{-}mal}$ Residue[a]	$\delta_{C\alpha,RC}$[b]	$\delta_{C\alpha}$, obs[c] [d]	$\Delta\delta_{C\alpha}$[e]	$\delta_{H\alpha,RC}$ [b]	$\delta_{H\alpha}$, obs[c] [d]	$\Delta\delta_{H\alpha}$[e]	$\delta_{HN,RC}$ [b]	δ_{HN}, obs[c] [d]	$\Delta\delta_{HN}$[e]
E73	54.373	54.27 ± 0.245	-0.103	4.585	4.429 ± 0.029	-0.156	8.53	8.187 ± 0.008	-0.343
P74	60.976	63.27 ± 0.26	2.294	4.746	4.392 ± 0.024	-0.354			
P75	63.166	62.98 ± 0.18	-0.186	4.397	4.307 ±	-0.09			
S76	58.619	59.46 ± 0.33	0.841	4.346	4.217 ± 0.028	-0.129	8.43	8.320 ± 0.008	-0.11
W77	57.162	56.84 ± 0.37	-0.322	4.781	4.594 ± 0.021	-0.187	8.029	7.672 ± 0.002	-0.357
V78	62.253	63.74 ± 0.205	1.487	4.045	3.794 ± 0.025	-0.251	7.656	7.460 ± 0.013	-0.196
T79	62.041	63.2 ± 0.235	1.159	4.19	4.176 ± 0.029	-0.014	7.931	7.756 ± 0.028	-0.175
E80	56.753	56.8 ± 0.21	0.047	4.279	4.174 ± 0.005	-0.105	8.406	8.368 ± 0.007	-0.038
M81	55.54	55.92 ± 0.185	0.38	4.453	4.234 ± 0.023	-0.219	8.425	8.043 ± 0.015	-0.382
L82	55.132	56.76 ± 0.22	1.628	4.373	4.17 ± 0.041	-0.203	8.257	8.007 ± 0.013	-0.25
L83	55.249	55.91 ± 0.19	0.661	4.343	4.231 ± 0.003	-0.112	8.201	8.078 ± 0.013	-0.123
E84	56.537	56.8 ± 0.21	0.263	4.297	4.174 ± 0.033	-0.123	8.243	8.368 ± 0.007	0.125
N85	58.625	56.84 ± 0.37	-1.785	4.489	4.605 ± 0.022	0.116	8.381	7.933 ± 0.020	-0.448
E86	56.588	58.21 ± 0.31	1.622	4.215	4.122 ± 0.037	-0.093	8.438	8.204 ± 0.009	-0.234
L87	55.605	57.127 ± 0.218	1.522	4.255	4 ± 0.048	-0.255	8.262	7.817 ± 0.008	-0.445
W88	57.185	56.84 ± 0.37	-0.345	4.701	4.608 ± 0.016	-0.093	7.834	7.539 ± 0.009	-0.295
G89	58.134	56.84 ± 0.37	-1.294	4.433	4.563 ± 0.001	0.13	7.818	8.112 ± 0.027	0.294
S90	56.387	56.26 ± 0.27	-0.127	4.588	4.29 ± 0.017	-0.298	8.025	8.156 ± 0.017	0.131
P91	63.105	63.5 ± 0.235	0.395	4.484	4.355 ± 0.019	-0.129			
A92	52.666	52.93 ± 0.255	0.264	4.292	4.201 ± 0.008	-0.0912	8.331	8.333 ± 0.09	0.002
E93	56.715	56.8 ± 0.21	0.085	4.267	4.168 ± 0.031	-0.099	8.418	8.303 ± 0.001	-0.115
E94	56.799	56.8 ± 0.21	0.001	4.264	4.168 ±	-0.096	8.468	8.303 ± 0.001	-0.165

Table S3. Secondary chemical shifts in N-Myc $_{73\text{-}94\text{-}N85C/G89C\text{-}mal}$. [a] One letter code for amino acids. [b] Theoretical random coil $\delta_{C\alpha,RC}$, $\delta_{H\alpha,RC}$ and $\delta_{NH,RC}$ chemical shifts according to https://www1.bio.ku.dk/english/research/bms/sbinlab/randomchemicalshifts2/,[1–3] as calculated for N-Myc $_{73\text{-}94}$ at **25 °C** and pH= 7.5. [c] Observed experimental Cα, Hα and amide HN chemical shifts (ppm). [d] Error represents the uncertainly in peak centre determination (peak broad) as observed at the 1.2 contour level. [e]Calculated secondary (δ_{RC} - δ_{obs}) $\Delta\delta_{C\alpha}$, $\Delta\delta_{H\alpha}$ and $\Delta\delta_{NH}$ chemical shifts (ppm).

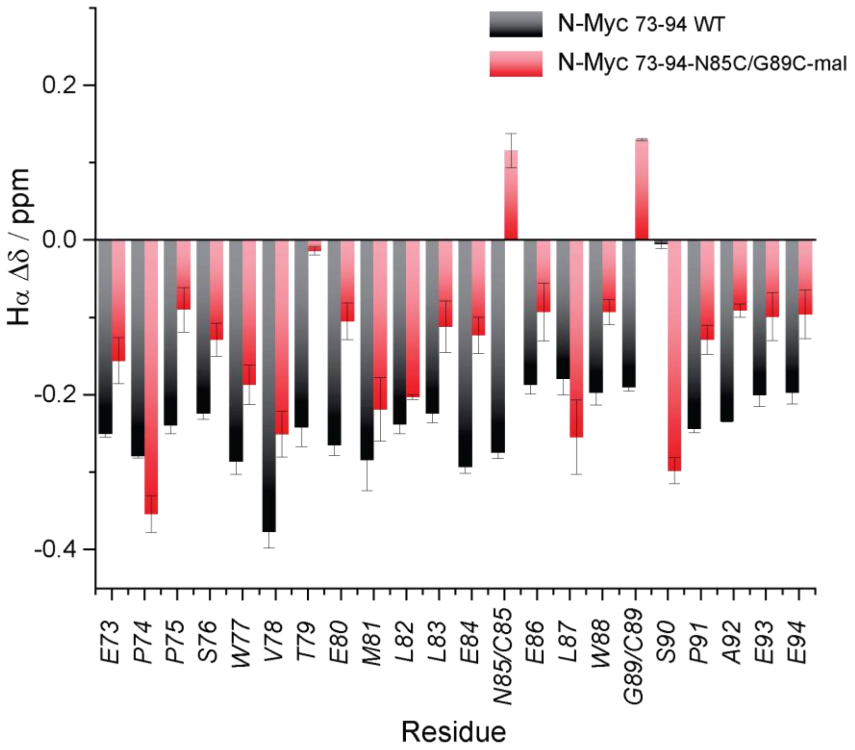

Figure S30. **Secondary ΔδHα chemical shifts by residue calculated for N-Myc $_{73\text{-}94}$ at 5 °C (black bars) and N-Myc $_{73\text{-}94\text{-}N85/G89C\text{-}mal}$ at 25 °C (red bars)** as based in their NMR ^{1}H and ^{1}H-^{13}C HSQC spectra.

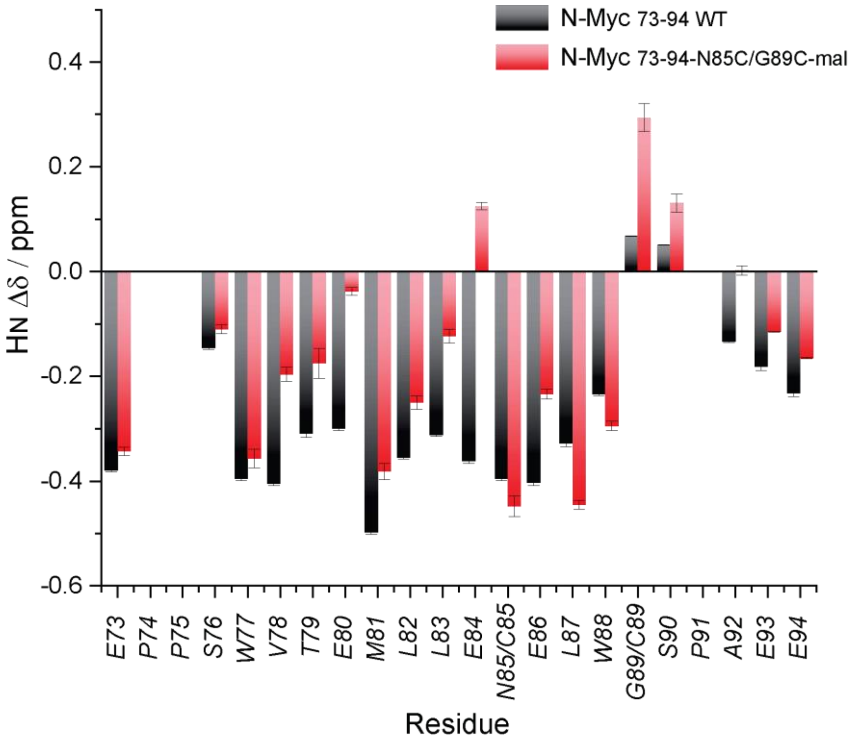

Figure S31. **Secondary ΔδN_H chemical shifts by residue calculated for N-Myc $_{73\text{-}94}$ at 5 °C (black bars) and N-Myc $_{73\text{-}94\text{-}N85/G89C\text{-}mal}$ at 25 °C (red bars)** as based in their NMR ^{1}H and ^{1}H-^{13}C HSQC spectra.

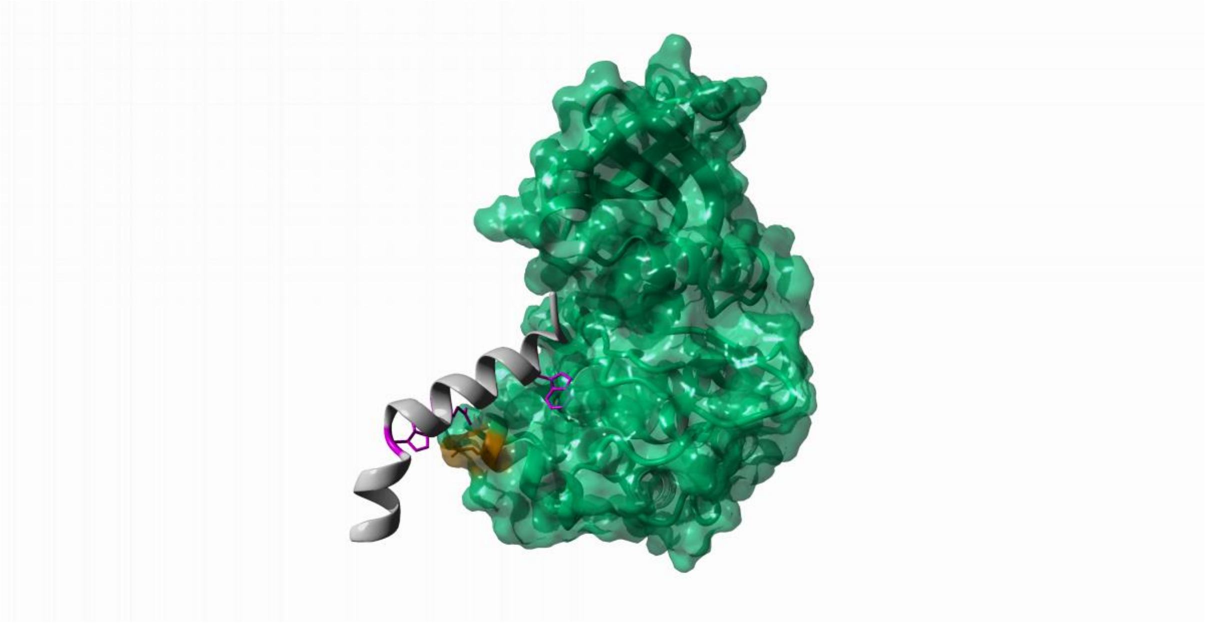

Figure S32. **Energy minimum structure of Aurora-A** $_{122\text{-}403\text{-}C290A/C393A}$ **/ N-Myc** $_{73\text{-}94}$.

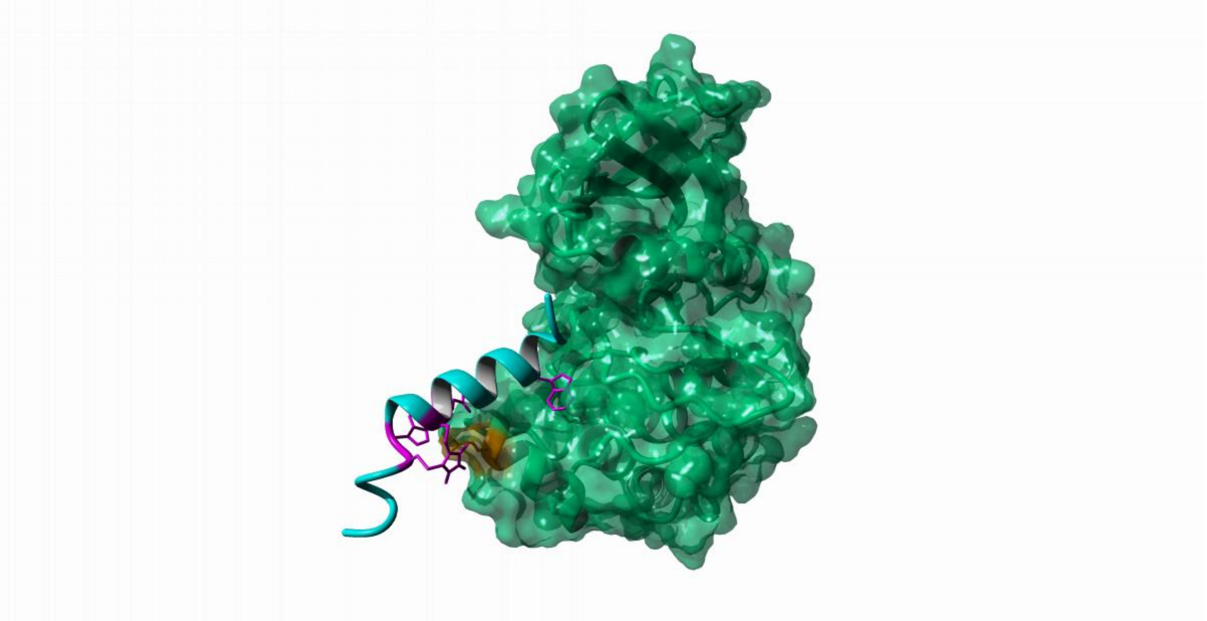

Figure S33. Energy minimum structure of Aurora-A $_{122\text{-}403\text{-}C290A/C393A}$ **/ N-Myc** $_{73\text{-}94\text{-}N85C/G89C\text{-}mal}$.

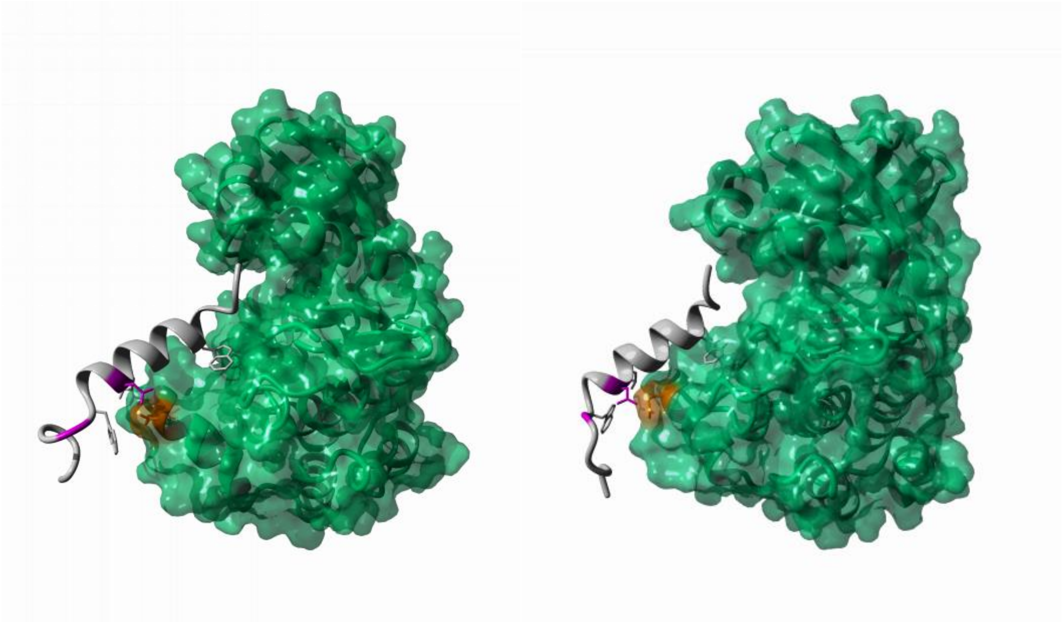

Figure S34. Average structure of Aurora-A $_{122\text{-}403\ \text{-C290A/C393A}}$ / N-Myc $_{73\text{-}94}$ after 80ns of simulation (320 frames).

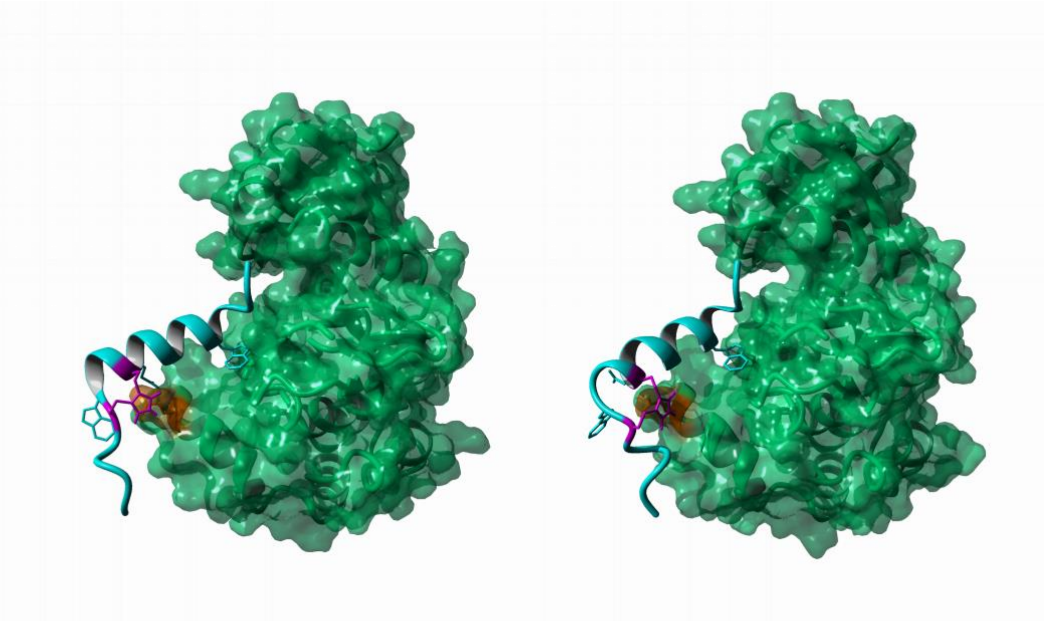

Figure S35. **Average structure of Aurora-A $_{122\text{-}403\ \text{-C290A/C393A}}$ / N-Myc $_{73\text{-}94\text{-N85C/G89C-mal}}$ after 80ns of simulation (320 frames).**

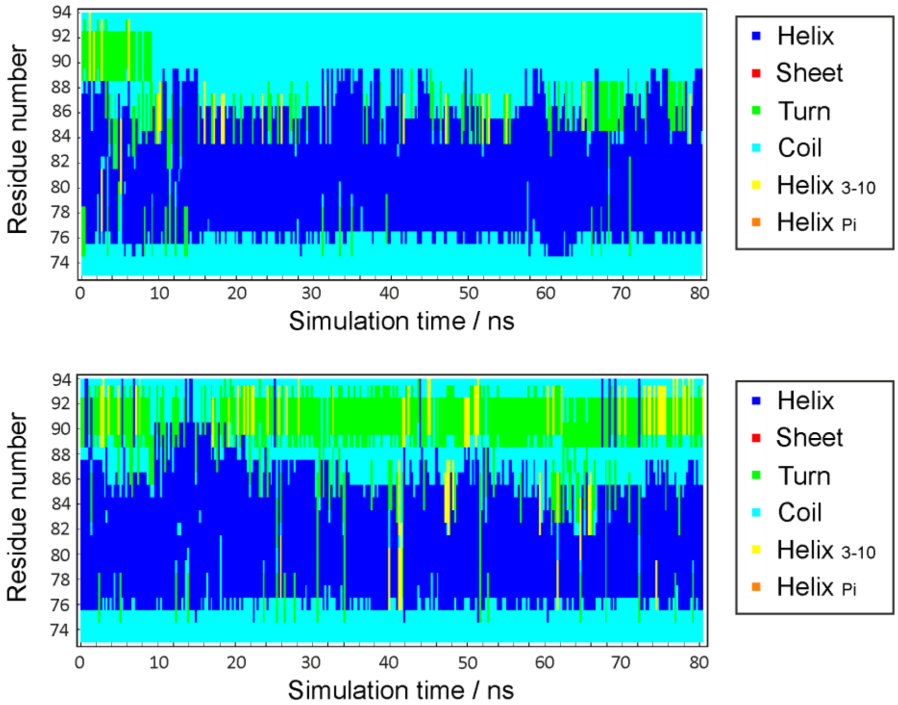

Figure S36. Replicate MD simulations of bound N-Myc$_{73\text{-}94}$ in Aurora-A $_{122\text{-}403\ \text{-C290A/C393A}}$ / N-Myc $_{73\text{-}94}$ complex.

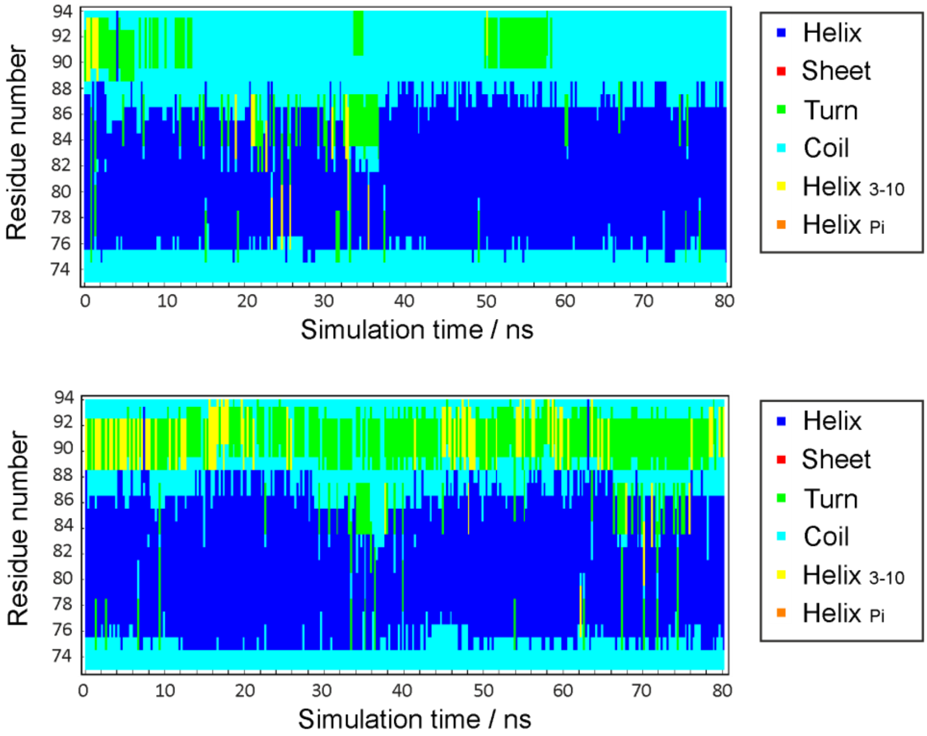

Figure S37. Replicate MD simulations of bound N-Myc$_{73\text{-}94}$ $^{N85C/G89C}$ mal in Aurora-A $_{122\text{-}403\ \text{-C290A/C393A}}$ / N-Myc $_{73\text{-}94\text{-N85C/G89C -mal}}$ complex.

Supplementary methods

Peptide NMR analysis: Peptide structure NMR studies were recorded on a Bruker AV4 NEO 11.75 T (500 MHz ^{1}H) NMR spectrometer (500-4C) at either 278 or 298 K, using water suppression by means of excitation sculpting with gradients using perfect echo.[24,25] ^{1}H-NMR spectra were obtained at 500 MHz using 128 scans with a relaxation delay of 1 s. ^{13}C{^{1}H}-NMR was obtained at 126 MHz with 0.8 s of relaxation delay. Bi-dimensional ^{1}H-^{1}H TOCSY experiments were performed using mixing times of 20 and 80 ms, a spectral width of 5,000 Hz in both dimensions and a minimum of 16 transients with 16 x 512 increments. Final minimum FT size = 2048 x 1024 points. Bi-dimensional ^{1}H-^{1}H COSY were obtained with a spectral width of 5,000 Hz in both dimensions using a minimum of 16 transients with 16 x 512 increments. Final minimum FT size = 2048 x 1024 points. Bi-dimensional ^{1}H-^{13}C HSQC experiments were performed with a spectral width of 5,000 x 10,000 Hz in both dimensions and a minimum of 64 transients with 64 x 64 increments. Final minimum FT size = 4096 x 1024 points. 1H-1H NOESY experiments were performed with a mixing time of 300 ms, a spectral width of 5,000 Hz in both dimensions and a minimum of 16 transients with 16 x 512 increments. Final minimum FT size = 2048 x 1024 points. All NMR data were processed using Topspin 4.1.4 analysis software.

Abbreviations

DMF Dimethylformamide
DIPEA N,N-Diisopropylethylamine
HCTU O-(1H-6-Chlorobenzotriazole-1-yl)-1,1,3,3-tetramethyluronium hexafluorophosphate
Oxyma Ethyl cyanohydroxyiminoacetate
DIC N,N'-Diisopropylcarbodiimide
TFA Trifluoroacetic acid
TIPS Triisopropyl silane
DODT 3,6-dioxa-1,8-octanedithiol
TCEP Tris Carboxy Ethyl Phosphene

References

[1] M. Kjaergaard, F. M. Poulsen, *J. Biomol. NMR* **2011**, *50*, 157–165.
[2] M. Kjaergaard, S. Brander, F. M. Poulsen, *J. Biomol. NMR* **2011**, *49*, 139–149.
[3] S. Schwarzinger, G. J. A. Kroon, T. R. Foss, J. Chung, P. E. Wright, H. J. Dyson, *J. Am. Chem. Soc.* **2001**, *123*, 2970–2978.

Summary of peptide characterisation

Below are tabulated HRMS data for all N-Myc peptides within this work. Peptide identity was confirmed by the inspection of multiple charge states which are quoted as the monoisotopic peak for the Expected (Exp) and Observed (Obs) masses.

Table S4. A summary of HRMS data of the synthesised peptides.

Peptide	$[M+H]^{1+}$ Obs	$[M+H]^{1+}$ Exp	$[M+2H]^{2+}$ Obs	$[M+2H]^{2+}$ Exp	$[M+3H]^{3+}$ Obs	$[M+3H]^{3+}$ Exp	$[M+4H]^{4+}$ Obs	$[M+4H]^{4+}$ Exp
**N-Myc $_{61\text{-}89}$ FAM**	3756.7088	3756.7061	1879.3549	1879.3531	1253.2415	1253.2354	940.1827	940.1765
**N-Myc $_{61\text{-}89}$**	3326.5851	3326.5787	1664.7973	1664.2894	1109.8668	1109.8596	832.9017	832.6447
**N-Myc $_{73\text{-}89}$**	2070.9990	2070.9841	1036.4995	1036.4921	691.3342	691.3280	-	518.7460
**N-Myc $_{76\text{-}89}$**	1748.8426	1748.8433	874.4240	875.4217	-	583.9478	-	438.2108
**N-Myc $_{73\text{-}94}$**	2584.2032	2584.1792	1293.1016	1293.0896	862.4037	862.3931	-	647.0448
**N-Myc $_{73\text{-}97}$**	2917.3390	2917.3237	1459.6695	1459.6619	973.4480	973.4412	-	730.3309
**N-Myc $_{73\text{-}89}^{E73pS}$**	2109.9415	2108.9399	1055.4774	1055.4700	703.9844	703.9800	-	528.2350
**N-Myc $_{73\text{-}89}^{E73S}$**	2029.9751	2028.9735	1015.4941	1015.4868	677.3289	677.3245	-	508.2434
**N-Myc $_{61\text{-}89}^{L83C/L87C}$ red**	3306.4337	3306.4289	1654.7219	1654.2145	1103.1499	1103.1430	827.6136	827.6072
**N-Myc $_{61\text{-}89}^{L83C/L87C}$ ox**	3304.4183	3304.4132	1653.7148	1653.2066	1102.4787	1102.4711	827.3612	827.1033
**N-Myc $_{61\text{-}89}^{L83C/L87C}$ mal**	3400.4292	3400.4212	1701.2146	1701.2106	1134.4789	1134.4737	850.8597	851.1053
**N-Myc $_{61\text{-}89}^{L82C/E86C}$ red**	3290.4792	3290.4704	1646.7420	1646.2352	1097.8317	1097.8235	823.6234	823.6176
**N-Myc $_{61\text{-}89}^{L82C/E86C}$ ox**	3288.4604	3288.4547	1645.7333	1645.2274	1097.1591	1097.1516	823.1196	823.1137
**N-Myc $_{61\text{-}89}^{L82C/E86C}$ mal**	3384.4696	3384.4627	1693.2348	1693.2314	1129.1607	1129.1542	-	847.1157
**N-Myc $_{61\text{-}89}^{M81C/N85C}$ red**	3288.5200	3288.5209	1645.2600	1645.2605	1096.8427	1097.1736	823.1323	823.1302
**N-Myc $_{61\text{-}89}^{M81C/N85C}$ ox**	3286.5146	3286.5052	1644.2573	1644.2526	1096.5082	1096.5017	822.6315	822.6263
**N-Myc $_{61\text{-}89}^{M81C/N85C}$ mal**	3381.5118	3381.5060	1691.7559	1691.7530	1128.1743	1128.1687	-	846.3765
**N-Myc $_{61\text{-}89}^{E80C/E84C}$ red**	3275.5254	3275.5191	1638.7627	1638.7596	1092.8451	1092.8397	819.8834	819.8798
**N-Myc $_{61\text{-}89}^{E80C/E84C}$ ox**	3273.5134	3273.5035	1637.7567	1637.7518	1092.5108	1092.1678	-	819.3759
**N-Myc $_{61\text{-}89}^{E80C/E84C}$ mal**	3368.5066	3368.5042	1685.2533	1685.2521	1123.8397	1123.8347	-	843.1261
**N-Myc $_{61\text{-}89}^{T79C/L83C}$ red**	3319.4729	3319.4726	1660.7410	1660.7363	1107.1624	1107.4909	830.8733	830.8682
**N-Myc $_{61\text{-}89}^{T79C/L83C}$ ox**	3317.4614	3317.4569	1659.7307	1659.7285	1106.8238	1106.8190	830.3689	830.3642
**N-Myc $_{61\text{-}89}^{T79C/L83C}$ mal**	3412.4619	3412.4576	1707.2346	1707.2288	1138.4927	1138.4859	854.1205	854.1144
**N-Myc $_{61\text{-}89}^{V78C/L82C}$ red**	3321.4578	3321.4518	1661.7289	1661.7259	1107.8218	1108.1506	831.3684	831.3630
**N-Myc $_{61\text{-}89}^{V78C/L82C}$ ox**	3319.4486	3319.4362	1660.7243	1660.7181	1107.4866	1107.4787	830.8653	830.8591
**N-Myc $_{61\text{-}89}^{V78C/L82C}$ mal**	3414.4379	3414.4369	1708.2225	1708.2185	1139.1512	1139.1456	-	854.6092
**N-Myc $_{61\text{-}89}^{S76C/E80C}$ red**	3317.5311	3317.5297	1659.7673	1659.7649	1106.5150	1106.8432	830.3884	830.3824
**N-Myc $_{61\text{-}89}^{S76C/E80C}$ ox**	3315.5292	3315.5140	1658.7646	1658.7570	1106.5153	1106.1713	830.1378	829.8785
**N-Myc $_{61\text{-}89}^{S76C/E80C}$ mal**	3410.5141	3410.5148	1706.2615	1706.2574	1137.8434	1137.8383	853.6329	853.6287
**N-Myc $_{73\text{-}94}^{E86C/S90C}$ red**	2574.1468	2574.1349	1288.0734	1288.0675	859.0508	859.0450	-	644.5337
**N-Myc $_{73\text{-}94}^{E86C/S90C}$ ox**	2572.1328	2572.1193	1287.0664	1287.0597	858.3796	858.3731	-	644.0298
**N-Myc $_{73\text{-}94}^{E86C/S90C}$ mal**	2667.1344	2667.1200	1334.5672	1334.5600	890.0463	890.0400	-	667.7800
**N-Myc $_{73\text{-}94}^{N85C/G89C}$ red**	2619.1564	2619.1452	1310.5782	1310.5726	874.0539	874.0484	-	655.7863
**N-Myc $_{73\text{-}94}^{N85C/G89C}$ ox**	2617.1420	2617.1295	1309.5710	1309.5648	873.3829	873.3765	-	655.2824
**N-Myc $_{73\text{-}94}^{N85C/G89C}$ mal**	2712.1428	2712.1303	1357.0714	1357.0652	905.0501	905.0434	-	679.0326

Analytical HPLC traces and high-resolution mass spectra of synthesised peptides

N-Myc $_{61-89}$ FAM

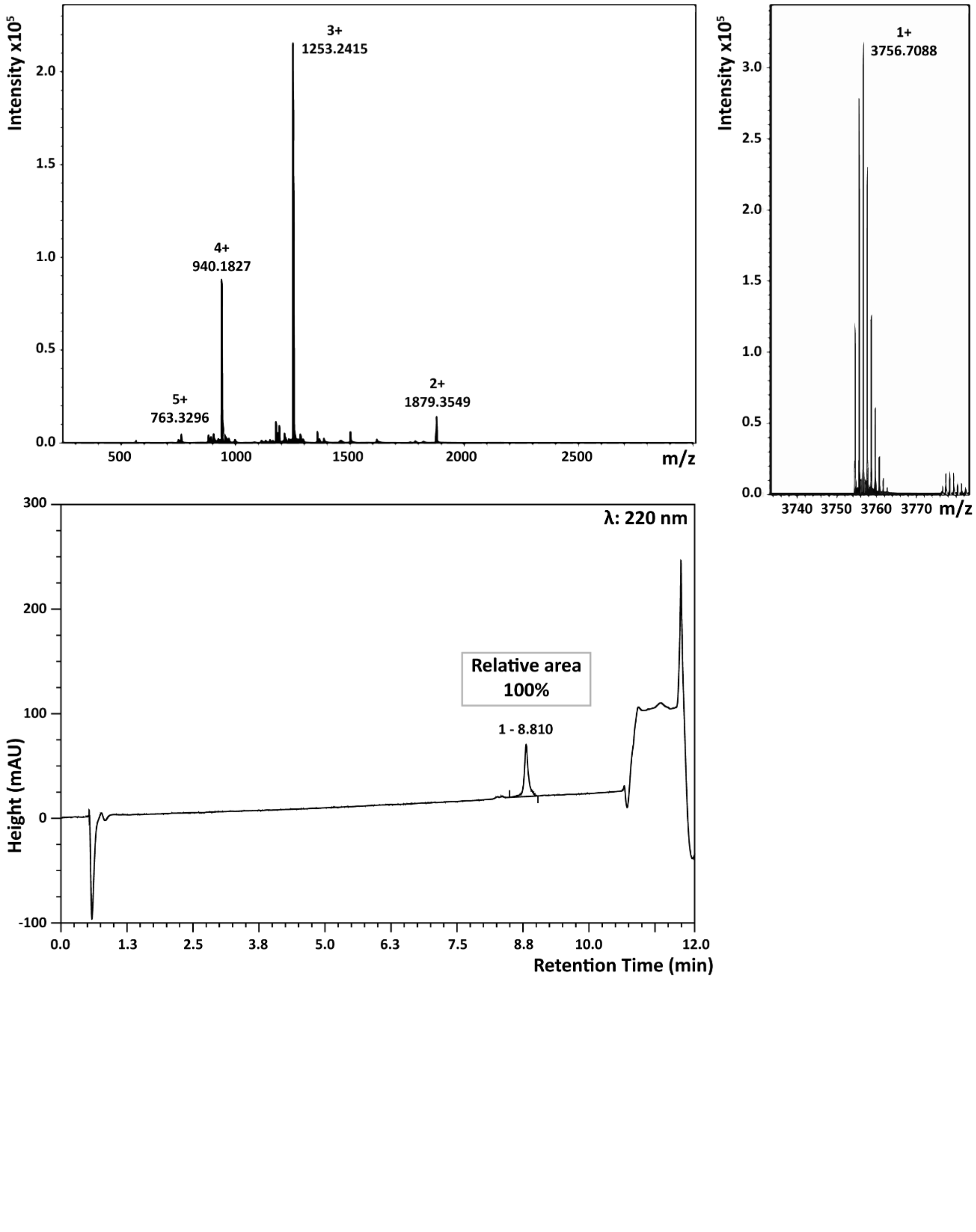

N-Myc $_{61-89}$

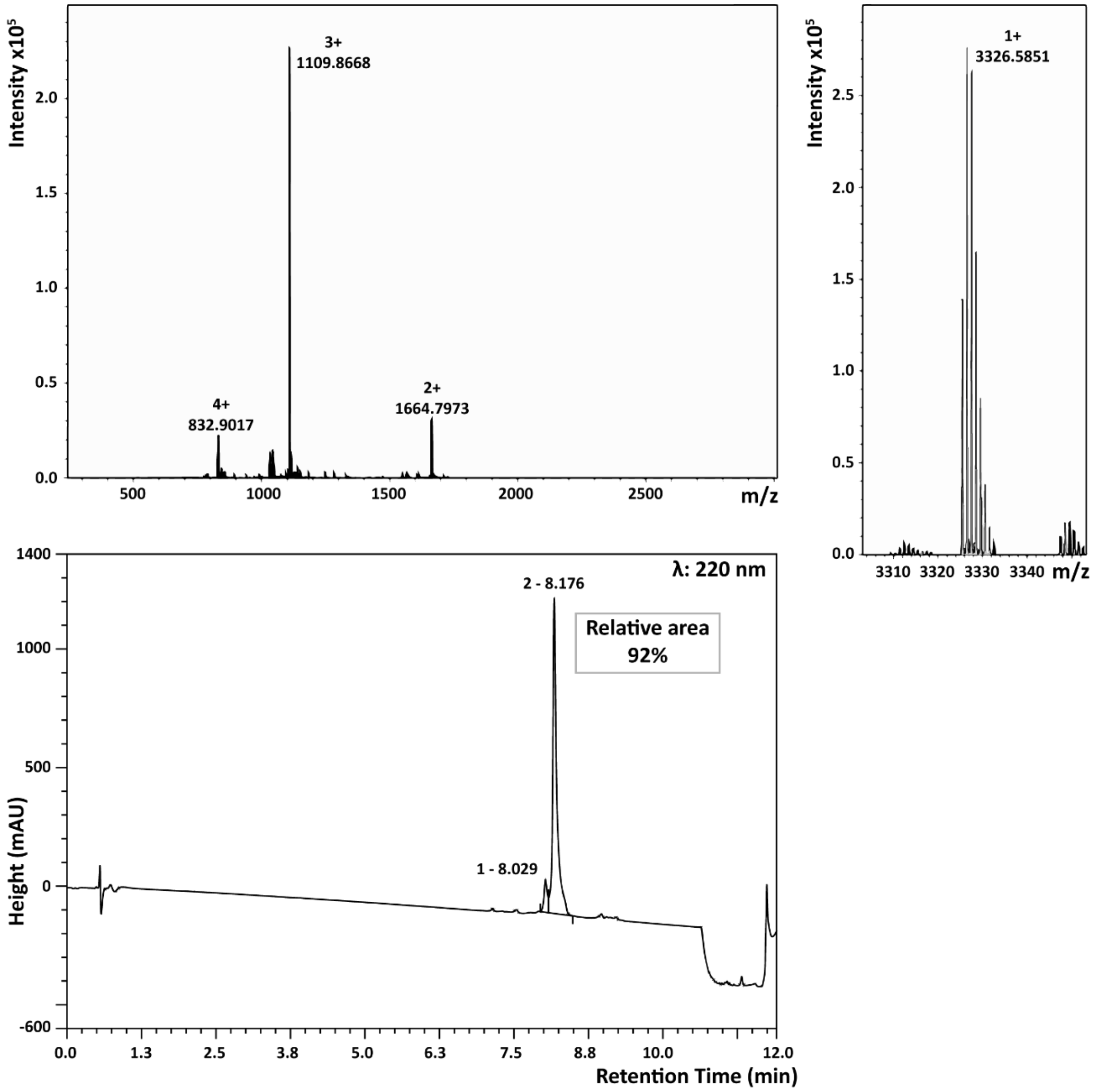
Intensity x10^5
3+
1109.8668
4+
832.9017
2+
1664.7973
m/z
1+
3326.5851
λ: 220 nm
2 - 8.176
1 - 8.029
Relative area
92%
Height (mAU)
Retention Time (min)

N-Myc $_{73\text{-}89}$

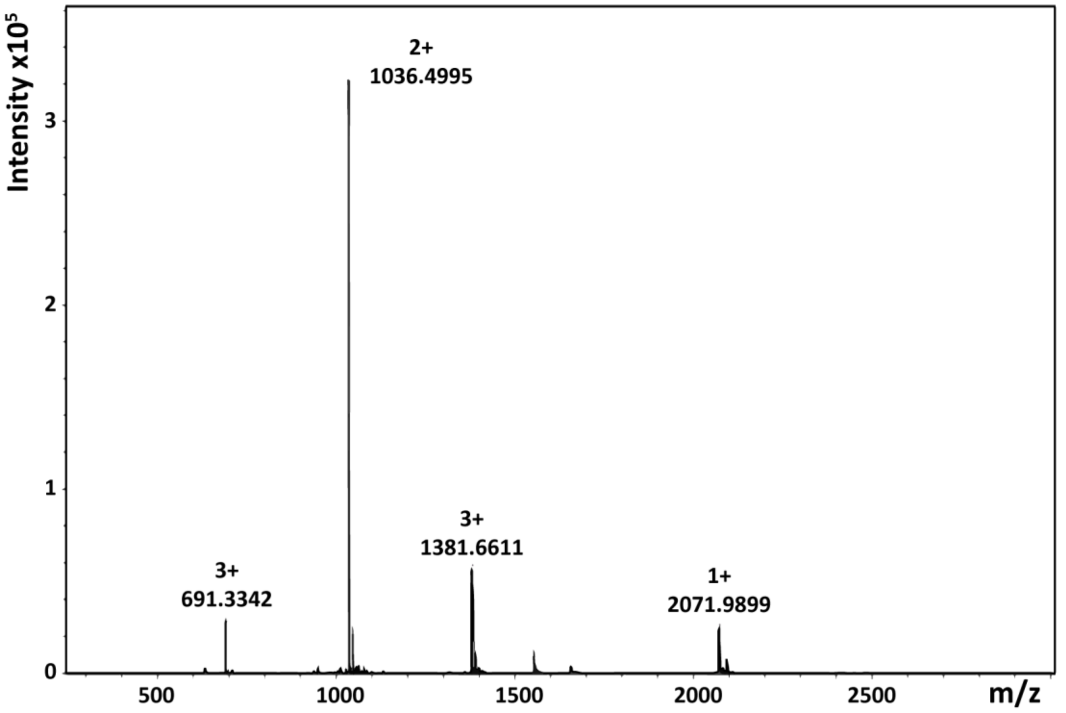

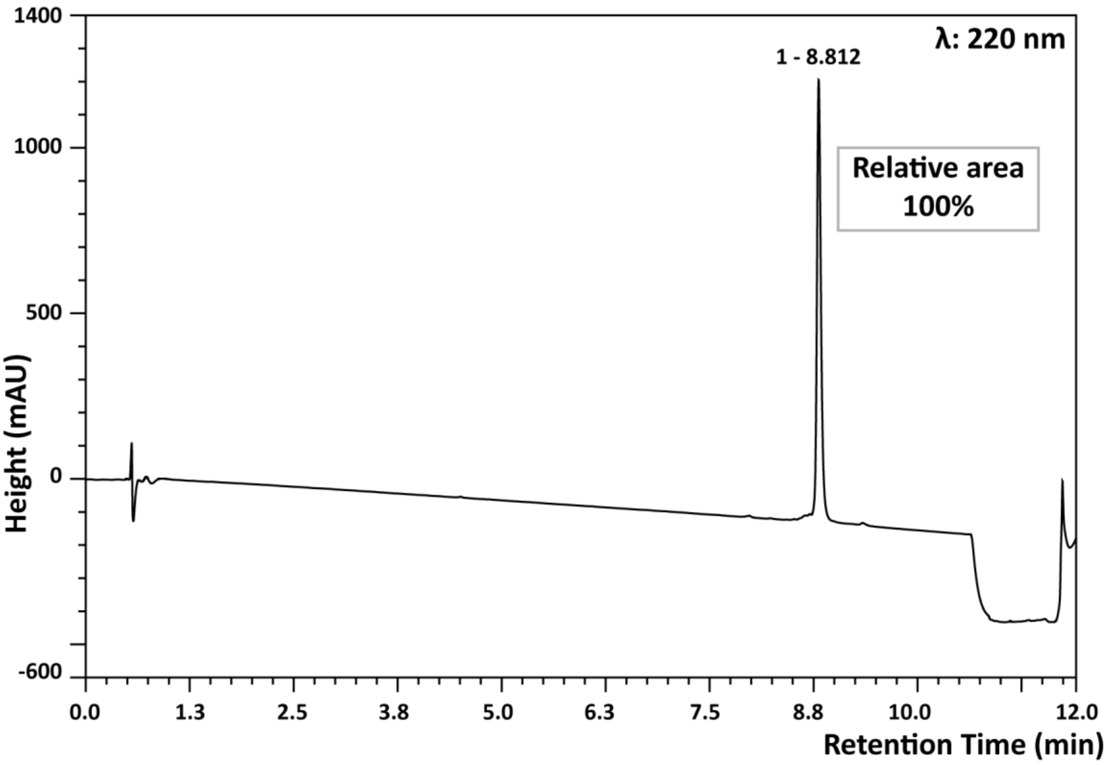

N-Myc $_{76\text{-}89}$

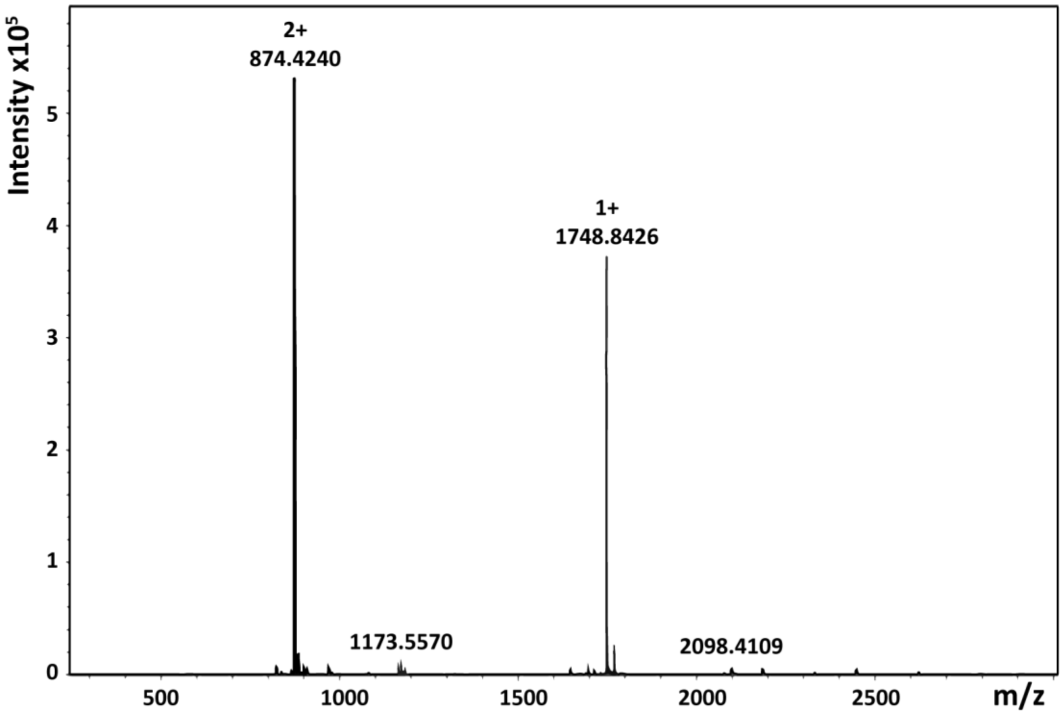

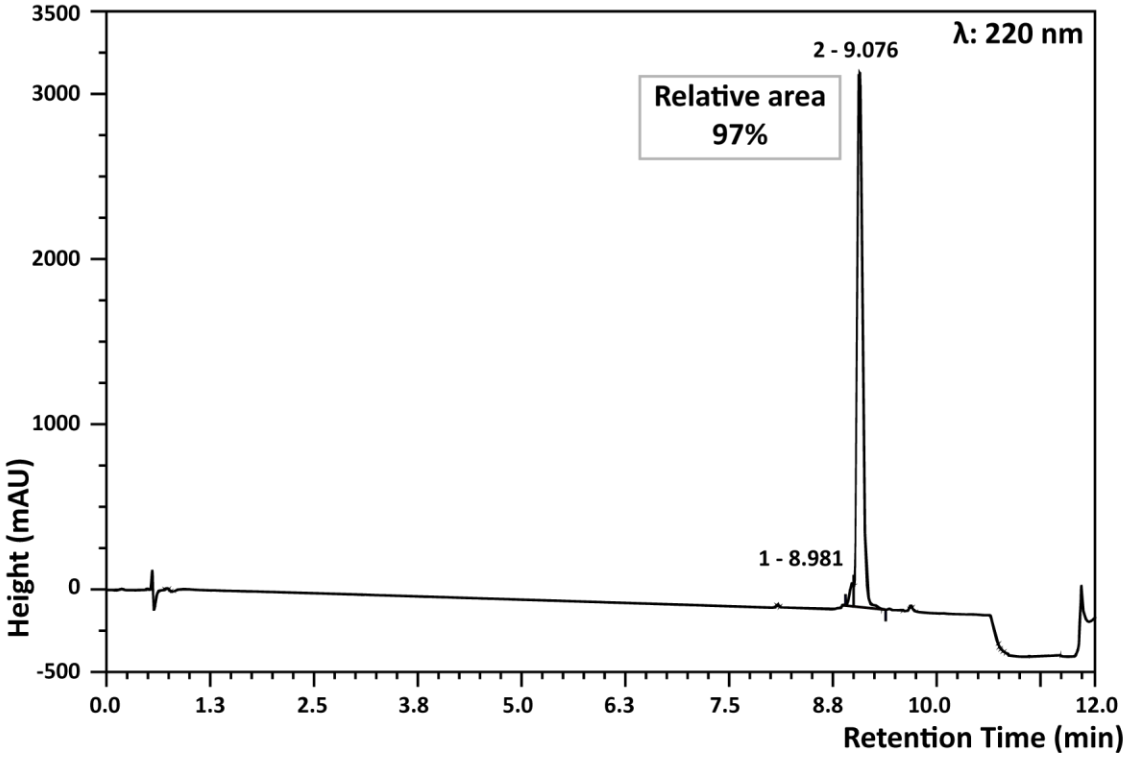

N-Myc $_{73-94}$

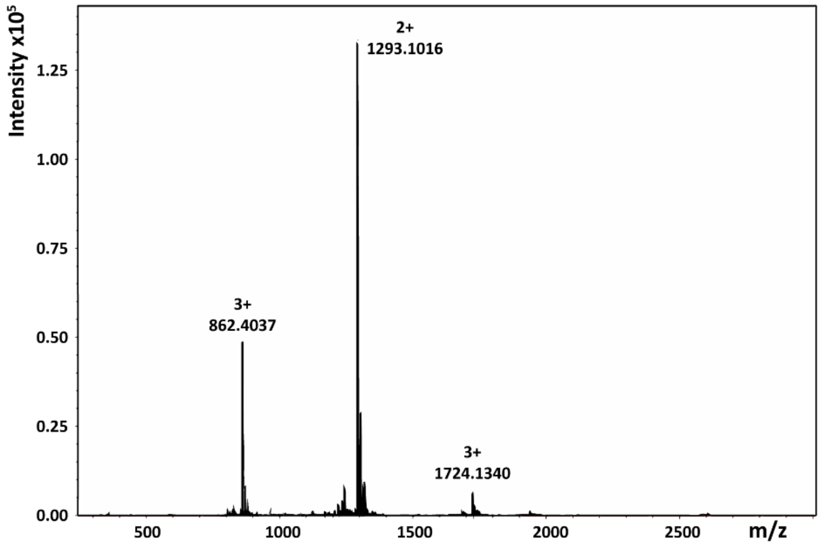
Intensity x10^5
2+
1293.1016
3+
862.4037
3+
1724.1340
m/z

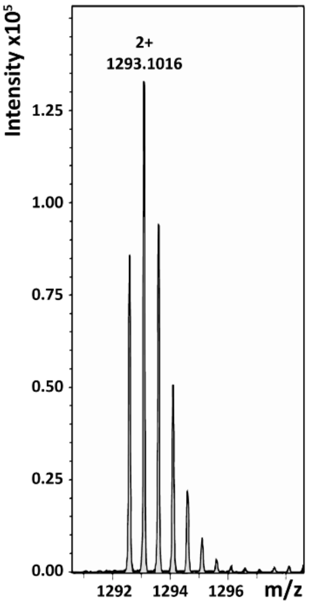
Intensity x10^5
2+
1293.1016
m/z

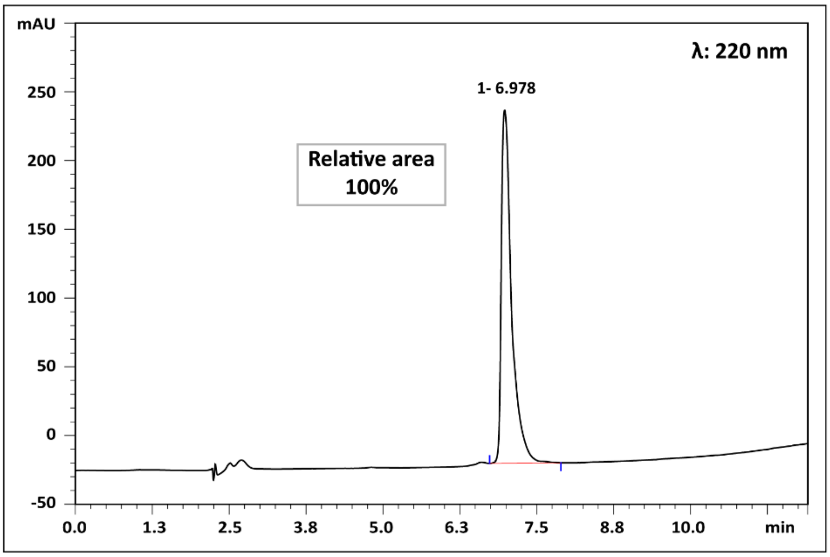
mAU
λ: 220 nm
1- 6.978
Relative area
100%
min

N-Myc $_{73-97}$

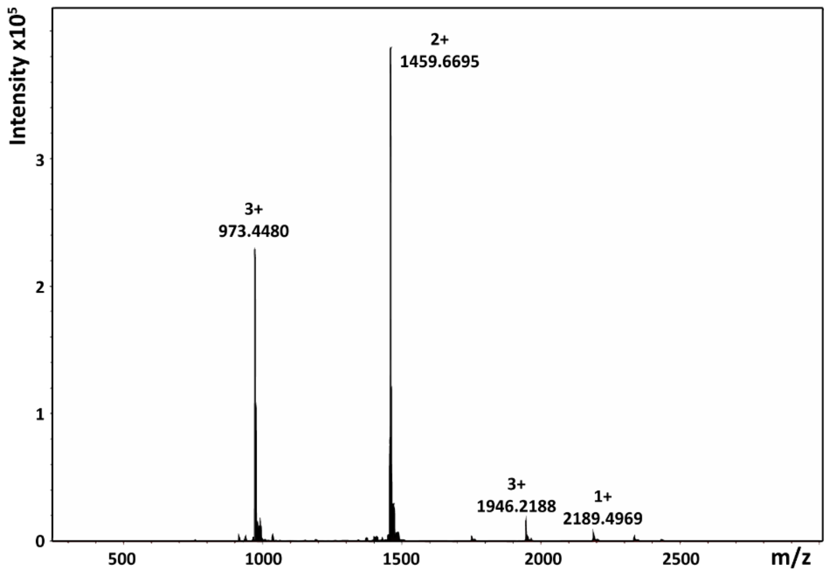

Intensity x10^5
3+
973.4480
2+
1459.6695
3+
1946.2188
1+
2189.4969
m/z

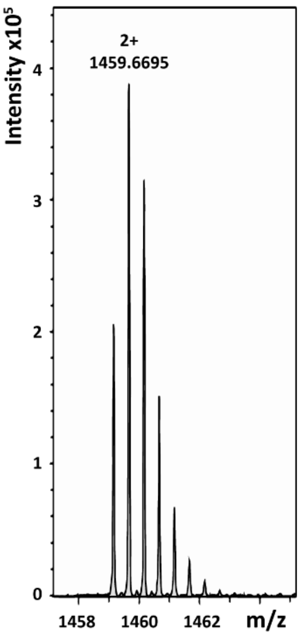

Intensity x10^5
2+
1459.6695
m/z

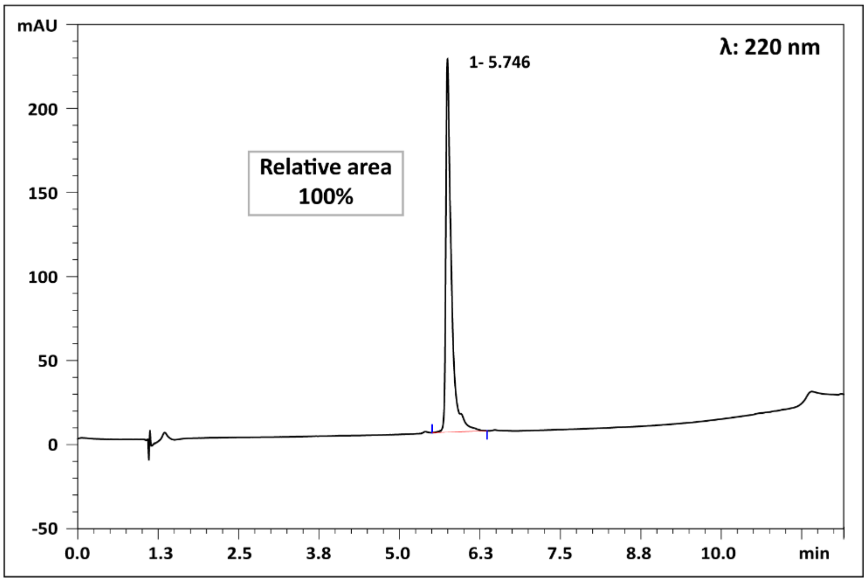

mAU
λ: 220 nm
1- 5.746
Relative area
100%
min

N-Myc $_{73\text{-}89}^{E73pS}$

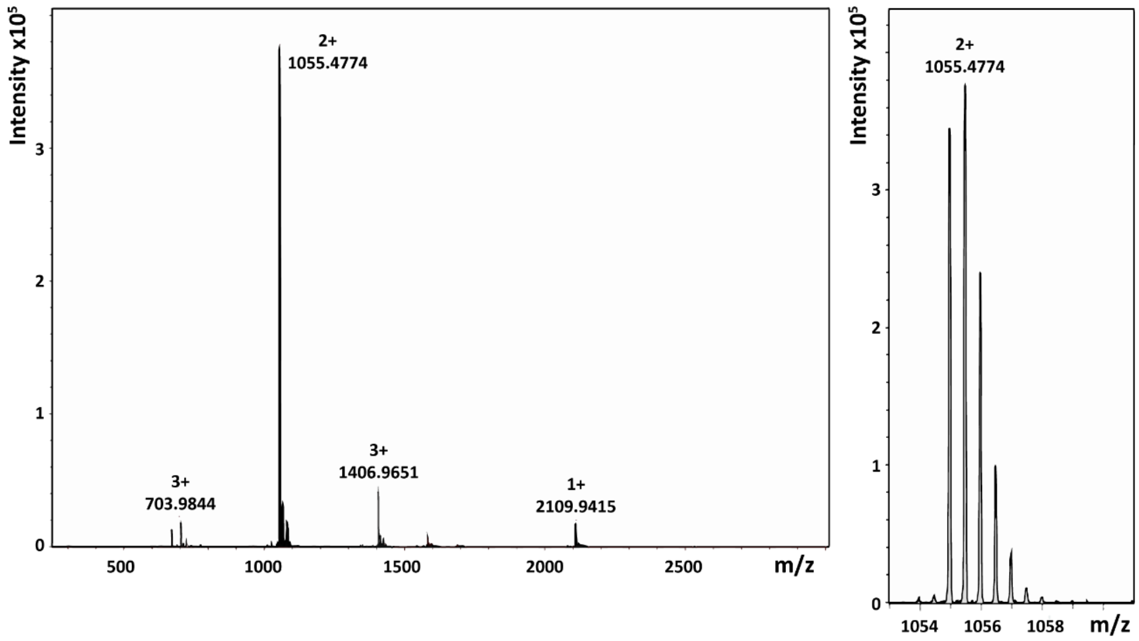

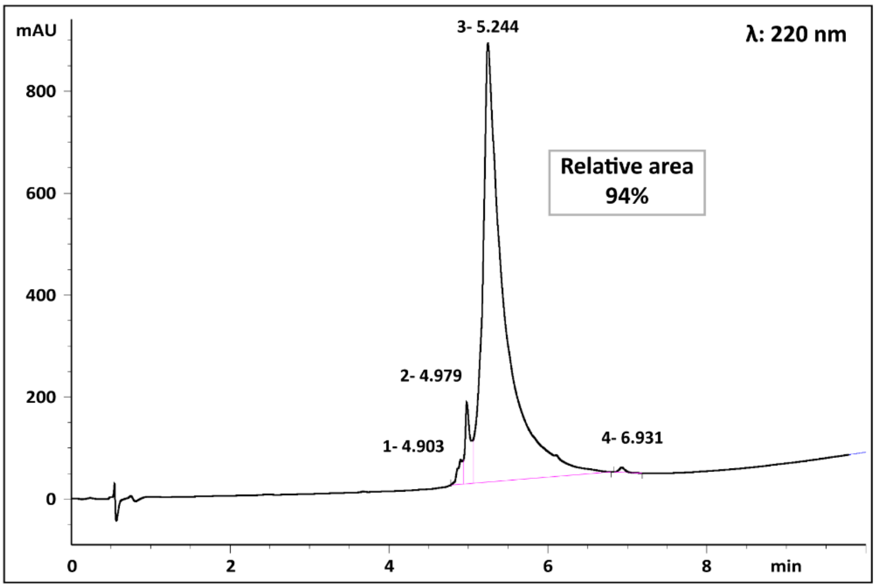

N-Myc $_{73\text{-}89}^{E73S}$

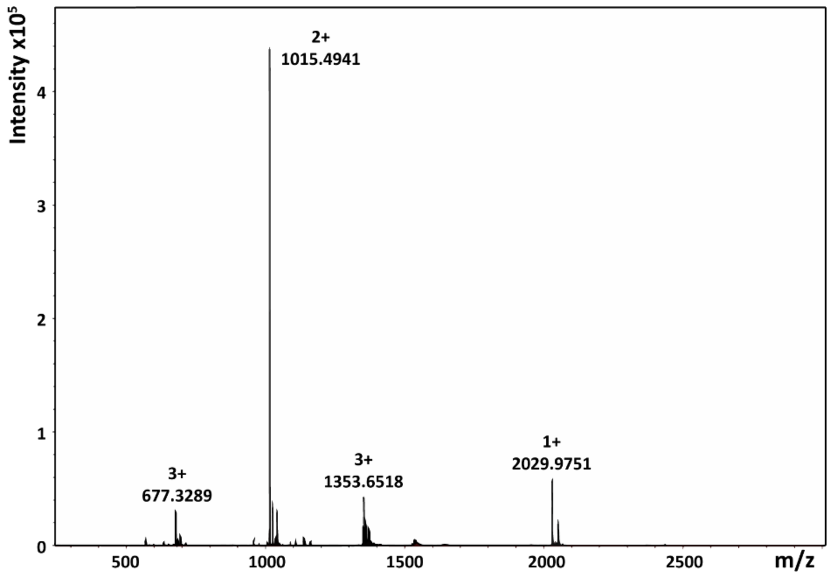

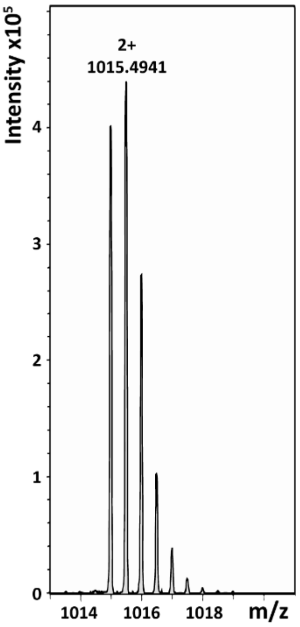

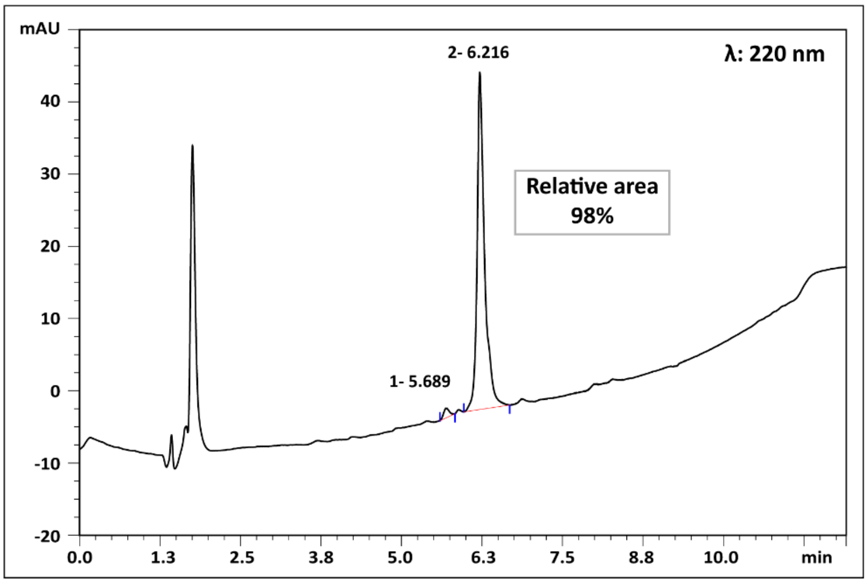

N-Myc $_{61\text{-}89}^{L83C/L87C}$ red

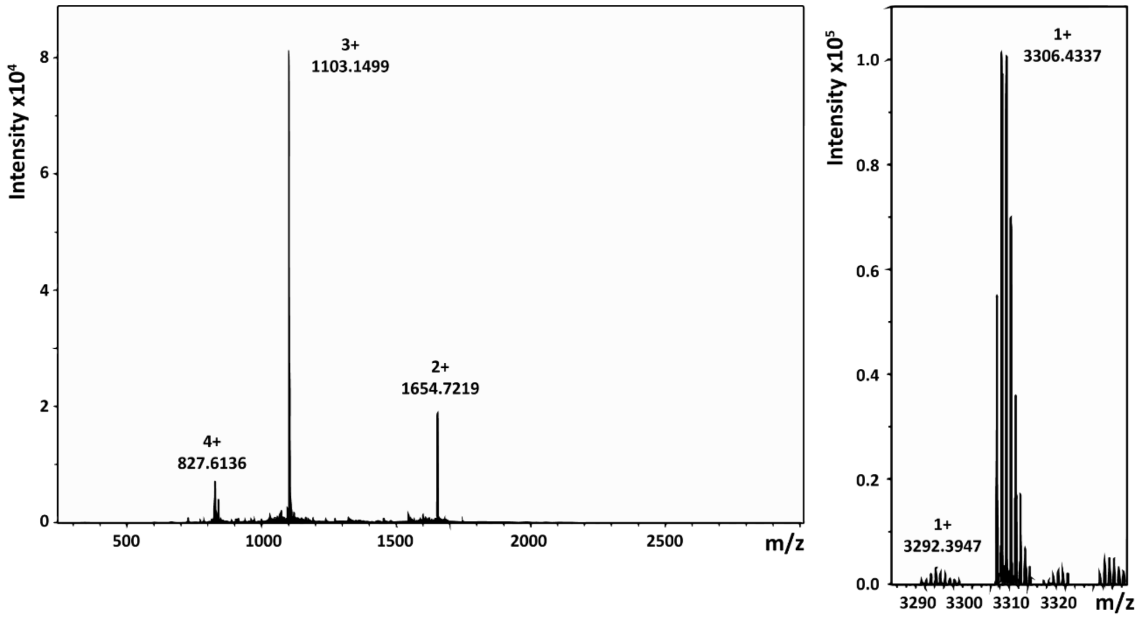

All free-thiol (red) and disulfide bridged (ox) variants originated from the same solid lyophilized sample; analytical HPLC spectra for disulfide bridged (ox) variants are provided.

N-Myc $_{61\text{-}89}^{L83C/L87C}$ ox

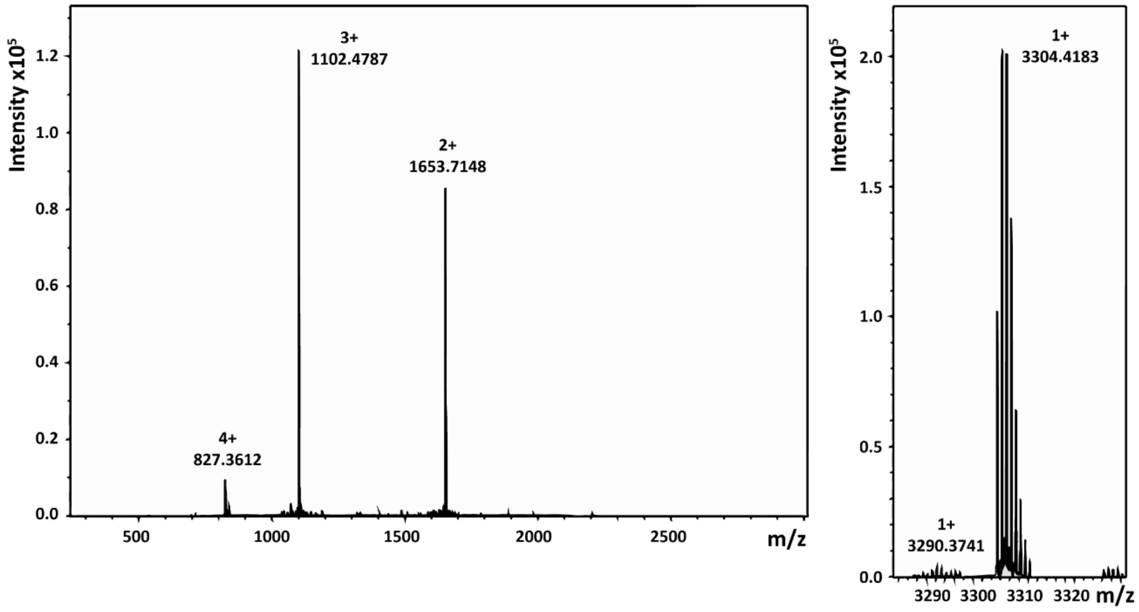

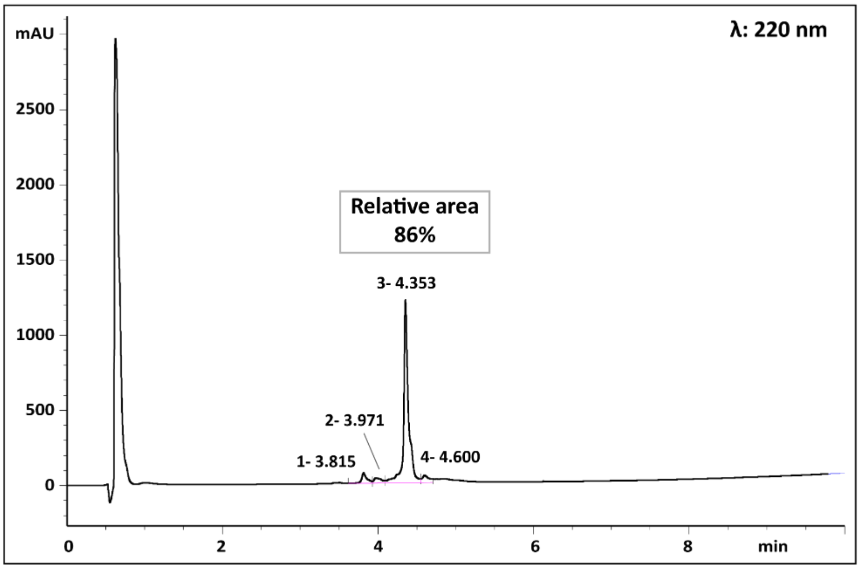

N-Myc $_{61-89}^{L83C/L87C}$ mal

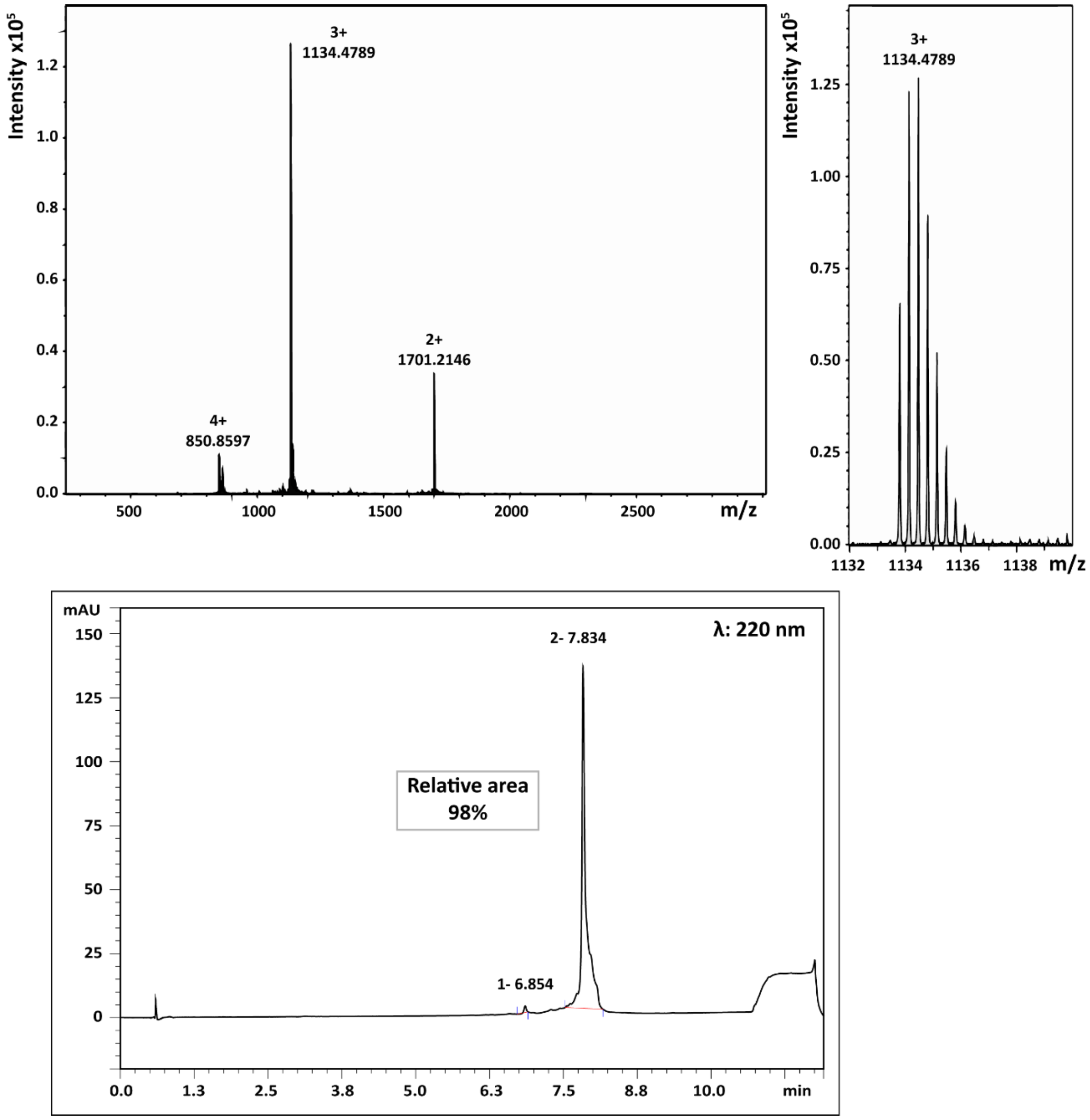

N-Myc $_{61\text{-}89}^{L82C/E86C}$ red

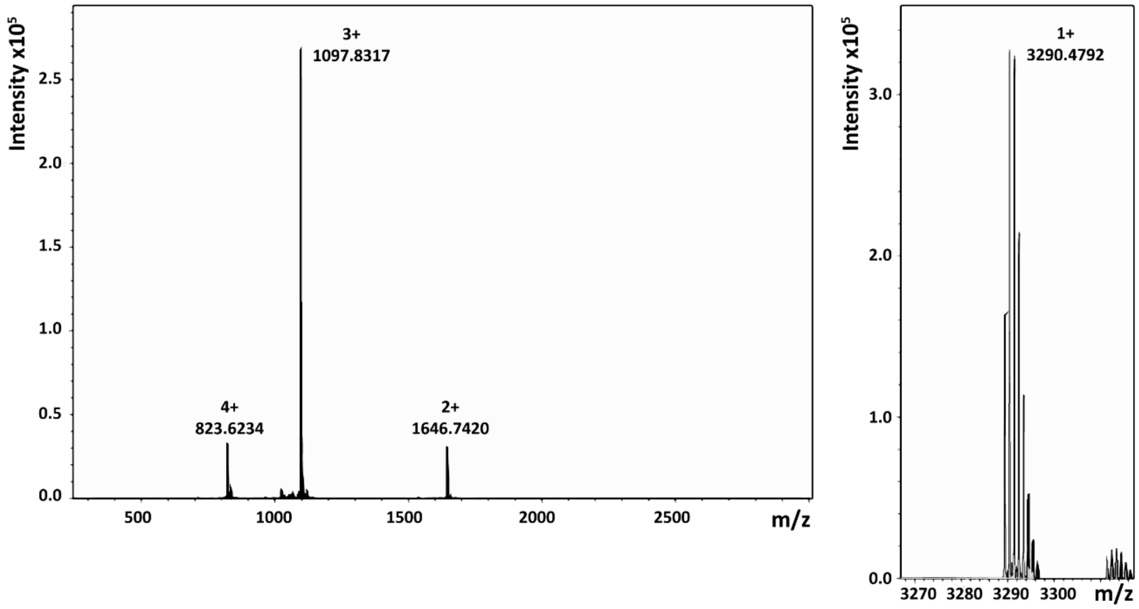

All free-thiol (red) and disulfide bridged (ox) variants originated from the same solid lyophilized sample; analytical HPLC spectra for disulfide bridged (ox) variants are provided.

N-Myc $_{61-89}$ $^{L82C/E86C}$ ox

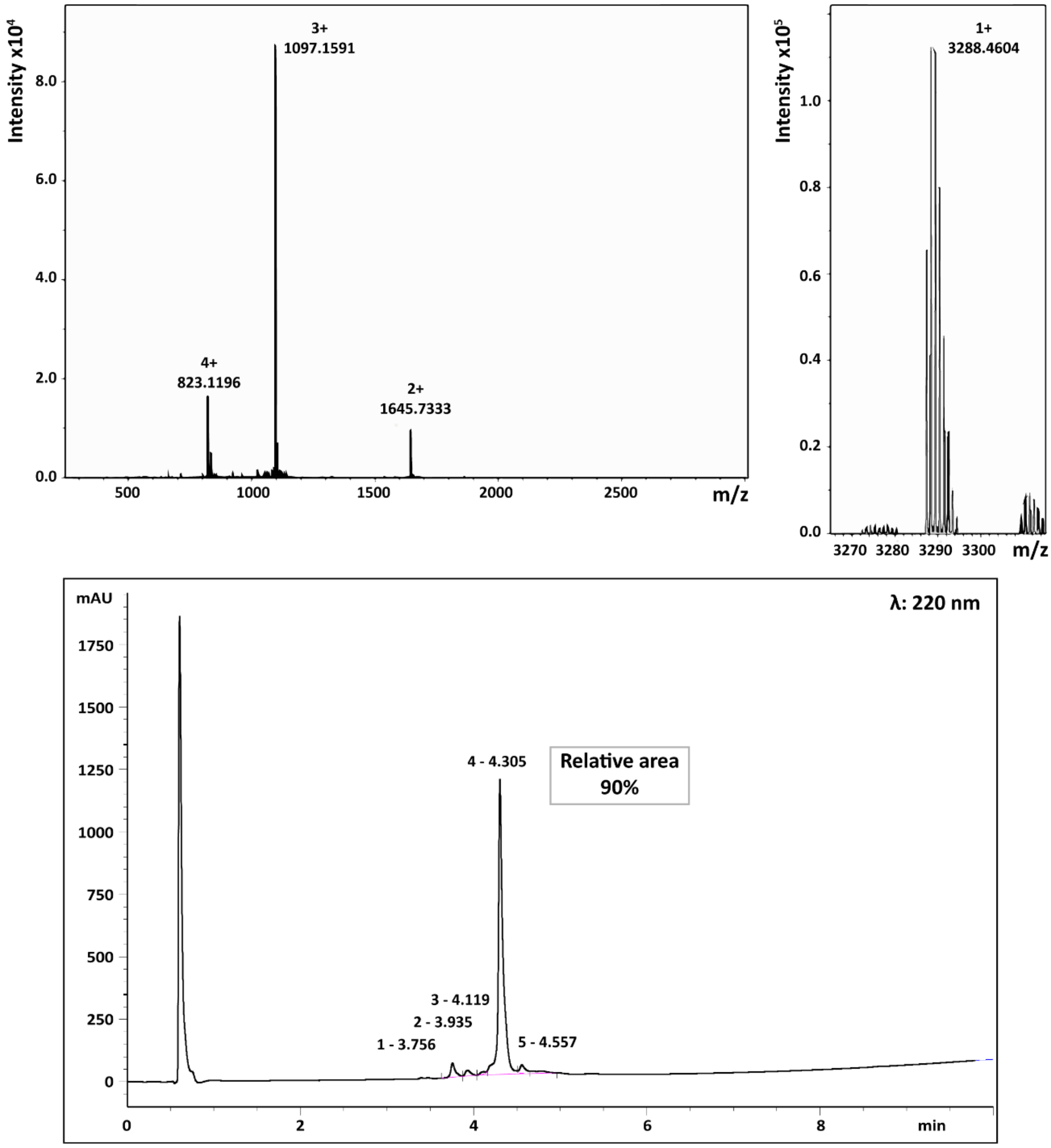

N-Myc $_{61\text{-}89}^{L82C/E86C}$ mal

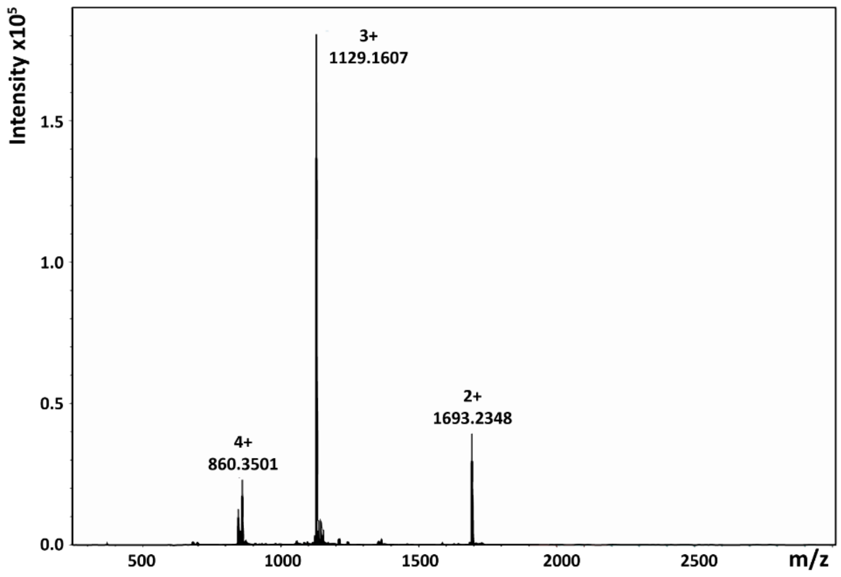

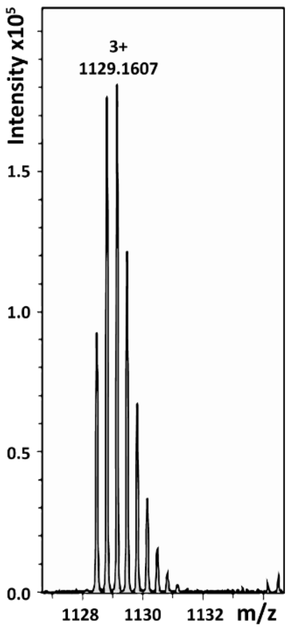

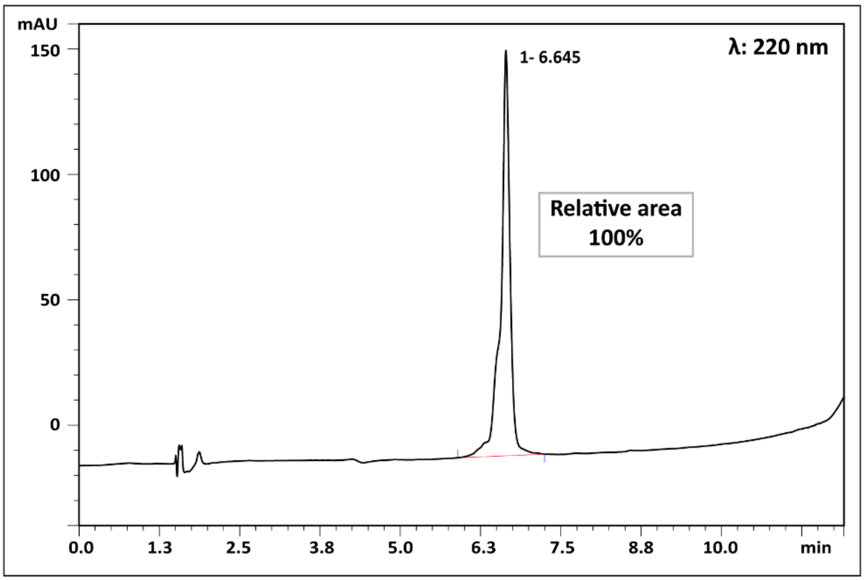

N-Myc $_{61\text{-}89}^{M81C/N85C}$ red

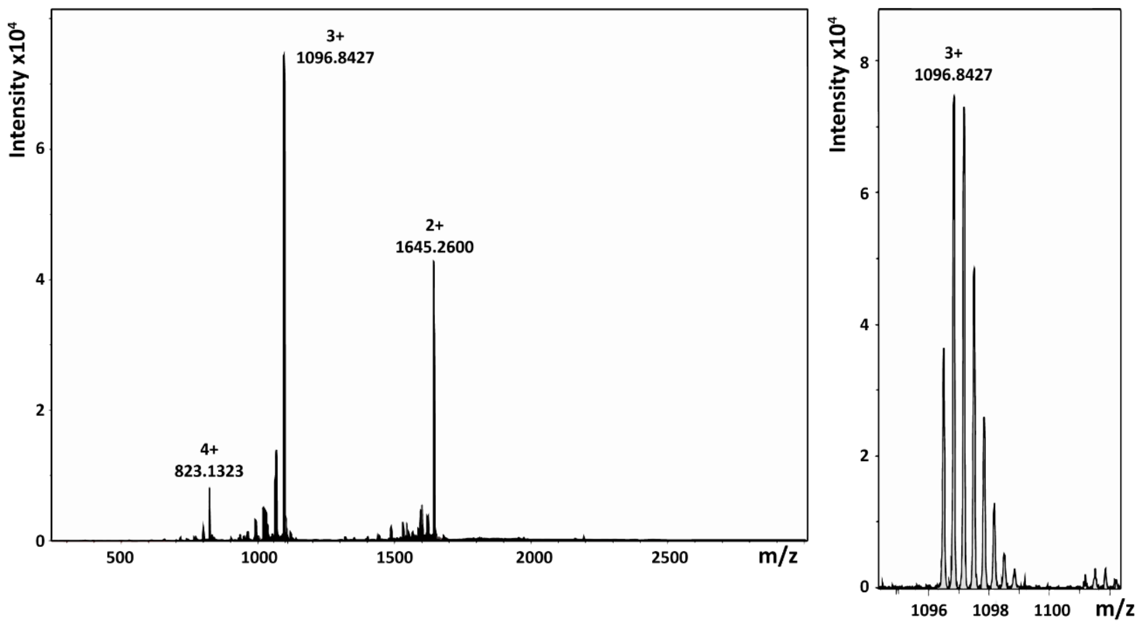

All free-thiol (red) and disulfide bridged (ox) variants originated from the same solid lyophilized sample; analytical HPLC spectra for disulfide bridged (ox) variants are provided.

N-Myc $_{61\text{-}89}^{M81C/N85C}$ ox

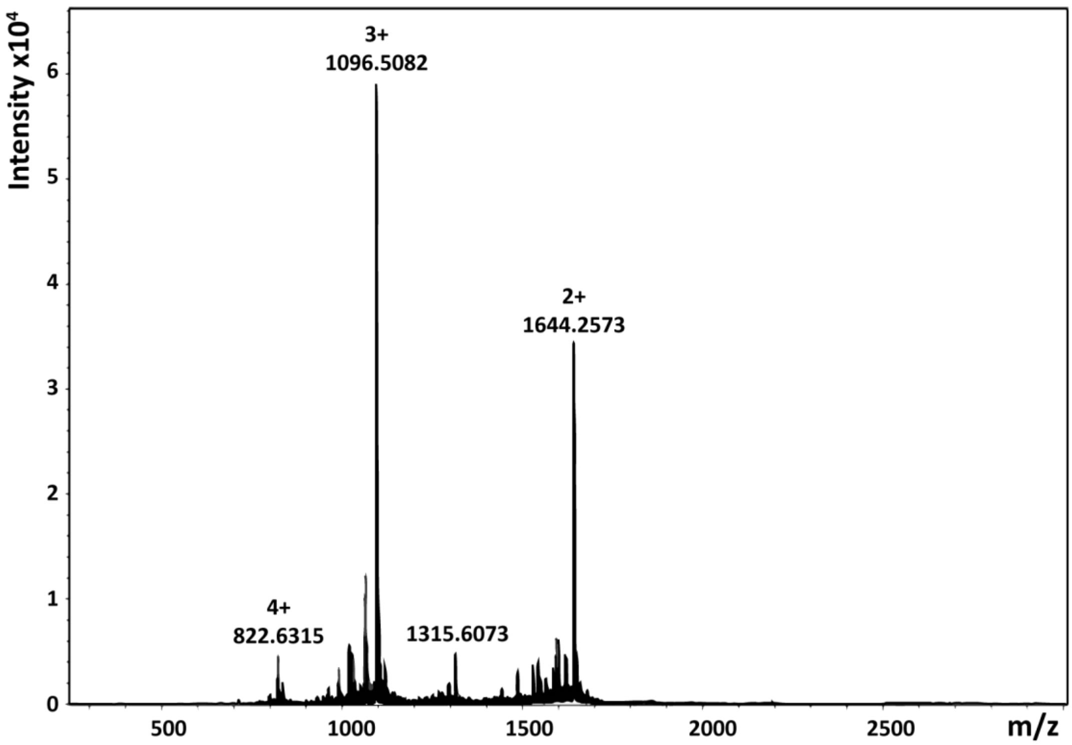

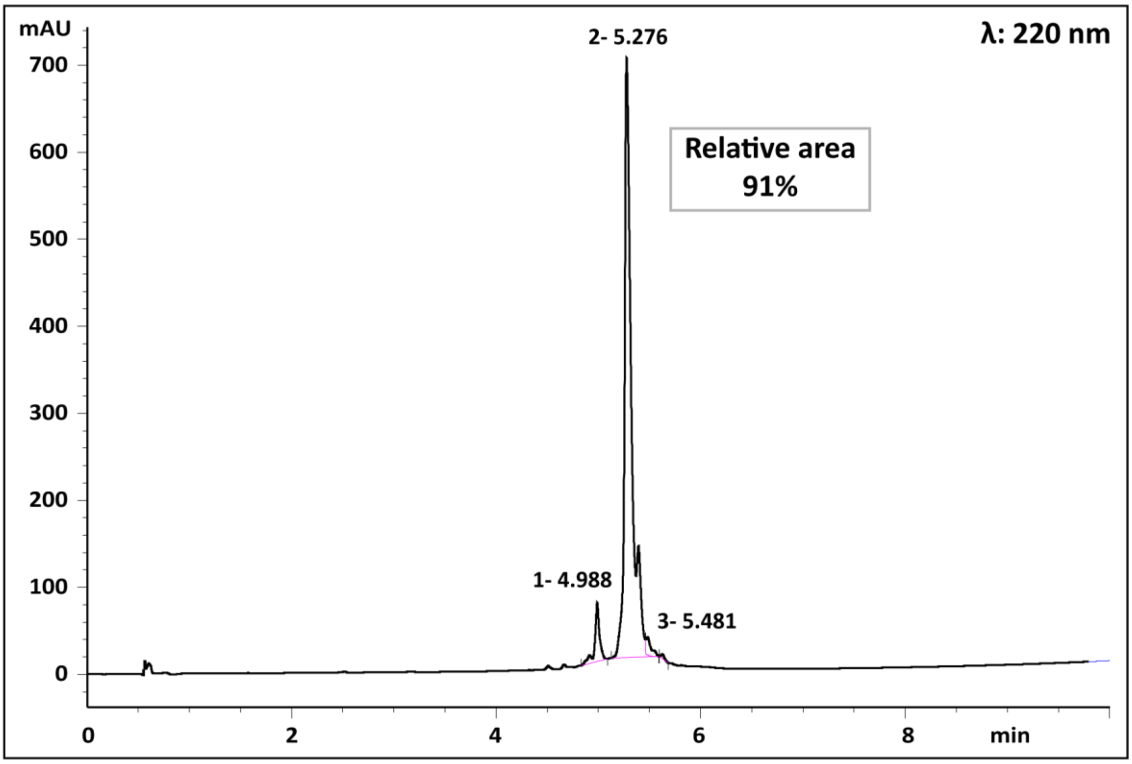

N-Myc $_{61\text{-}89}^{M81C/N85C}$ mal

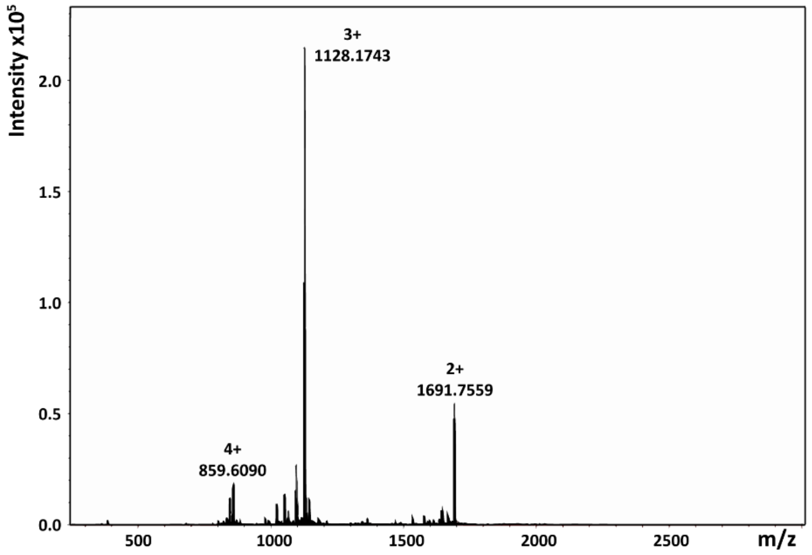

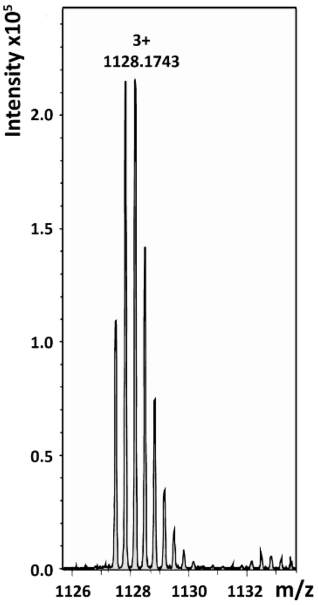

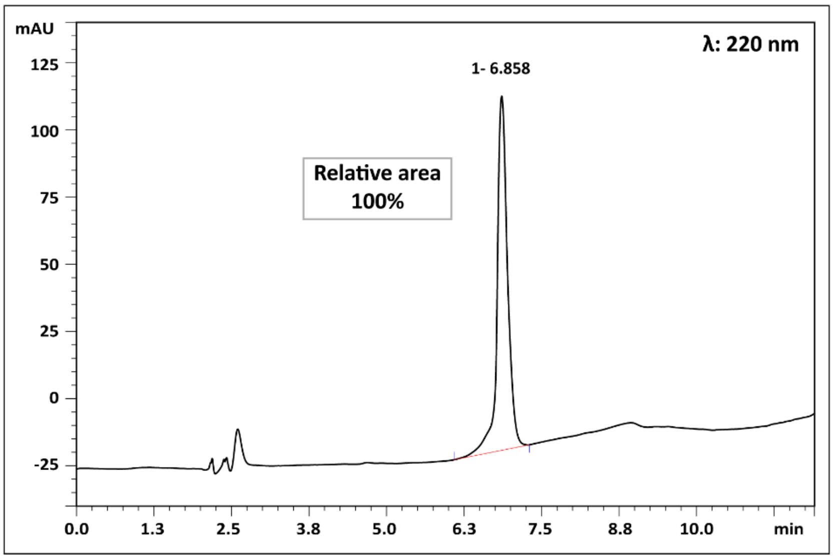

N-Myc $_{61-89}{}^{E80C/E84C}$ red

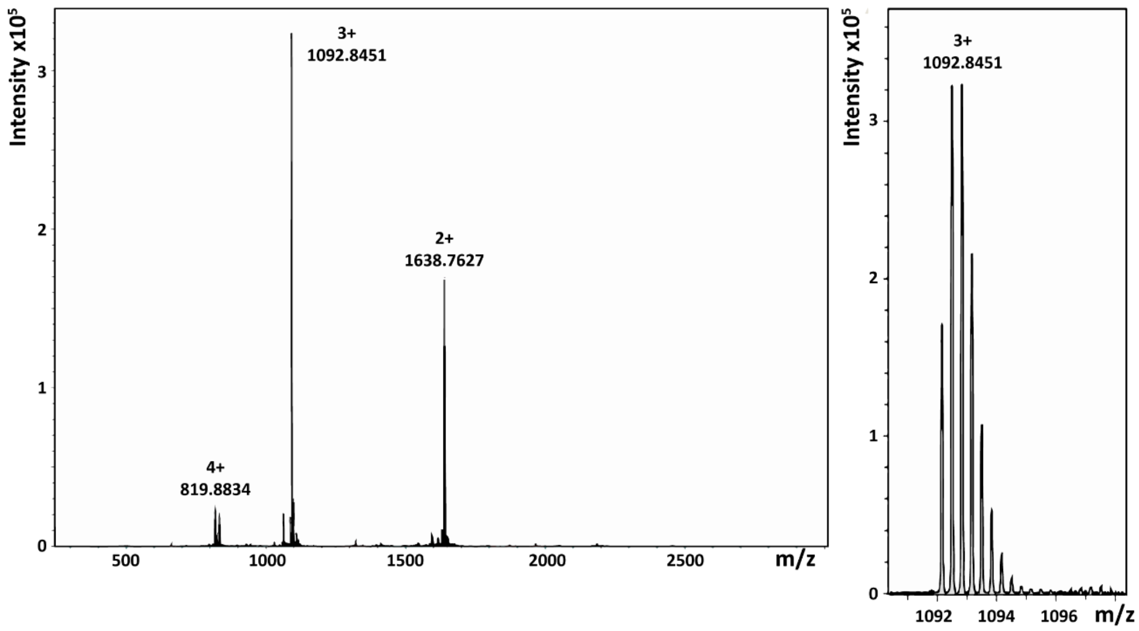

All free-thiol (red) and disulfide bridged (ox) variants originated from the same solid lyophilized sample; analytical HPLC spectra for disulfide bridged (ox) variants are provided.

N-Myc $_{61\text{-}89}^{E80C/E84C}$ ox

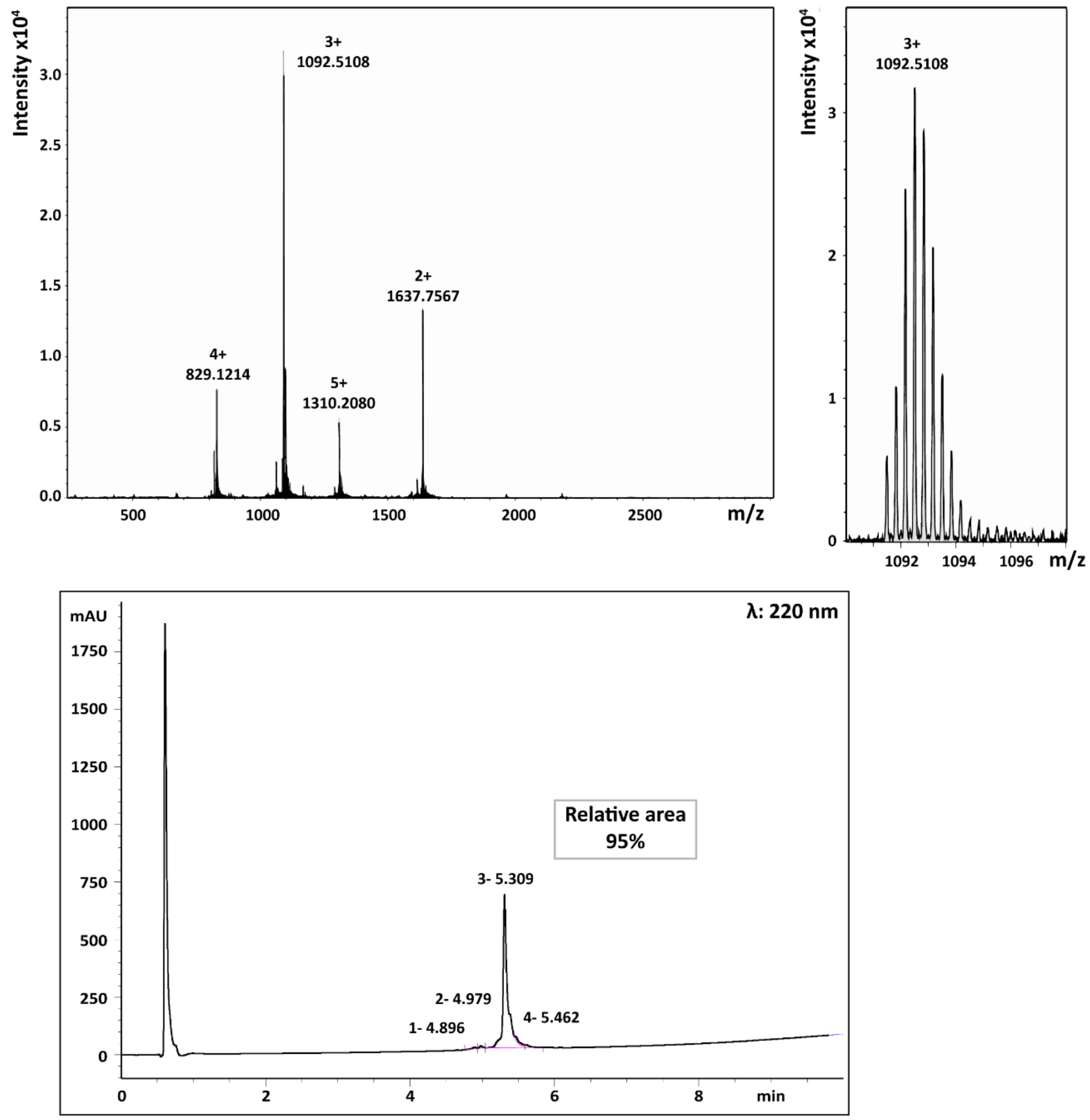

N-Myc $_{61\text{-}89}^{E80C/E84C}$ mal

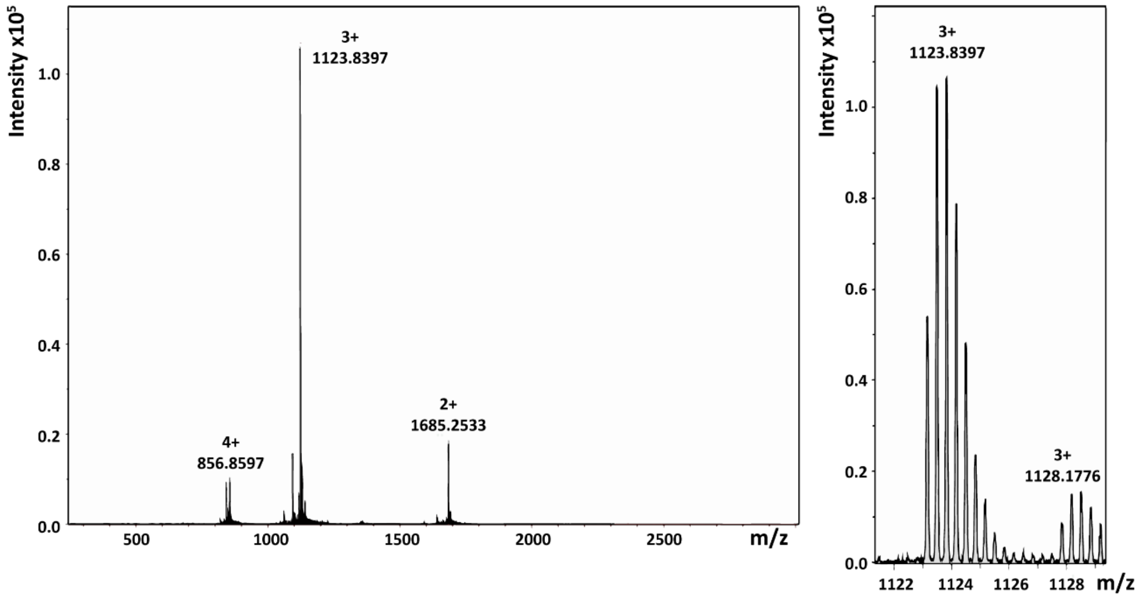

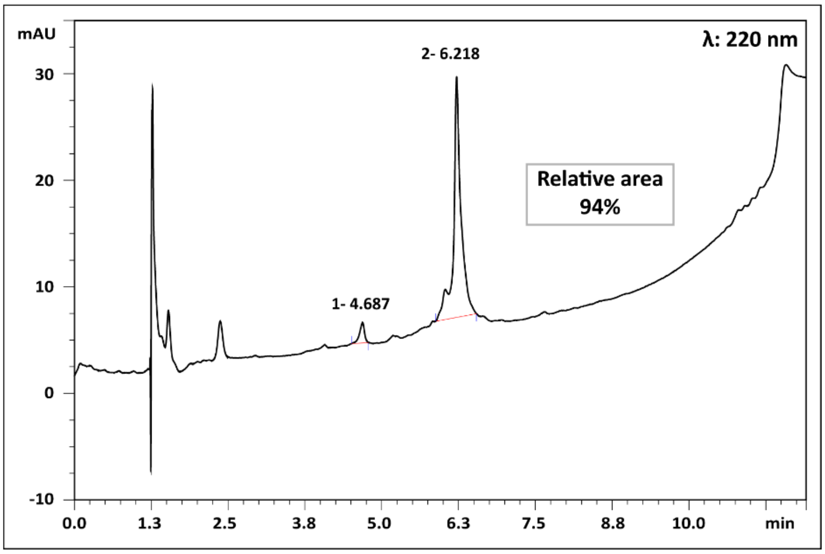

N-Myc $_{61\text{-}89}^{T79C/L83C}$ red

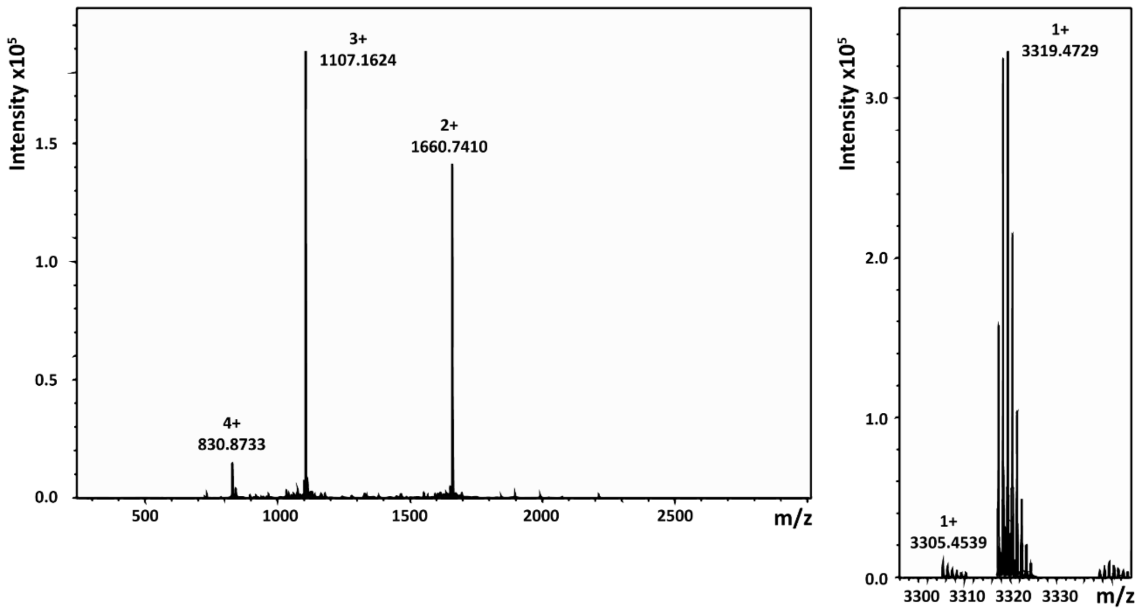

All free-thiol (red) and disulfide bridged (ox) variants originated from the same solid lyophilized sample; analytical HPLC spectra for disulfide bridged (ox) variants are provided.

N-Myc $_{61\text{-}89}^{T79C/L83C}$ ox

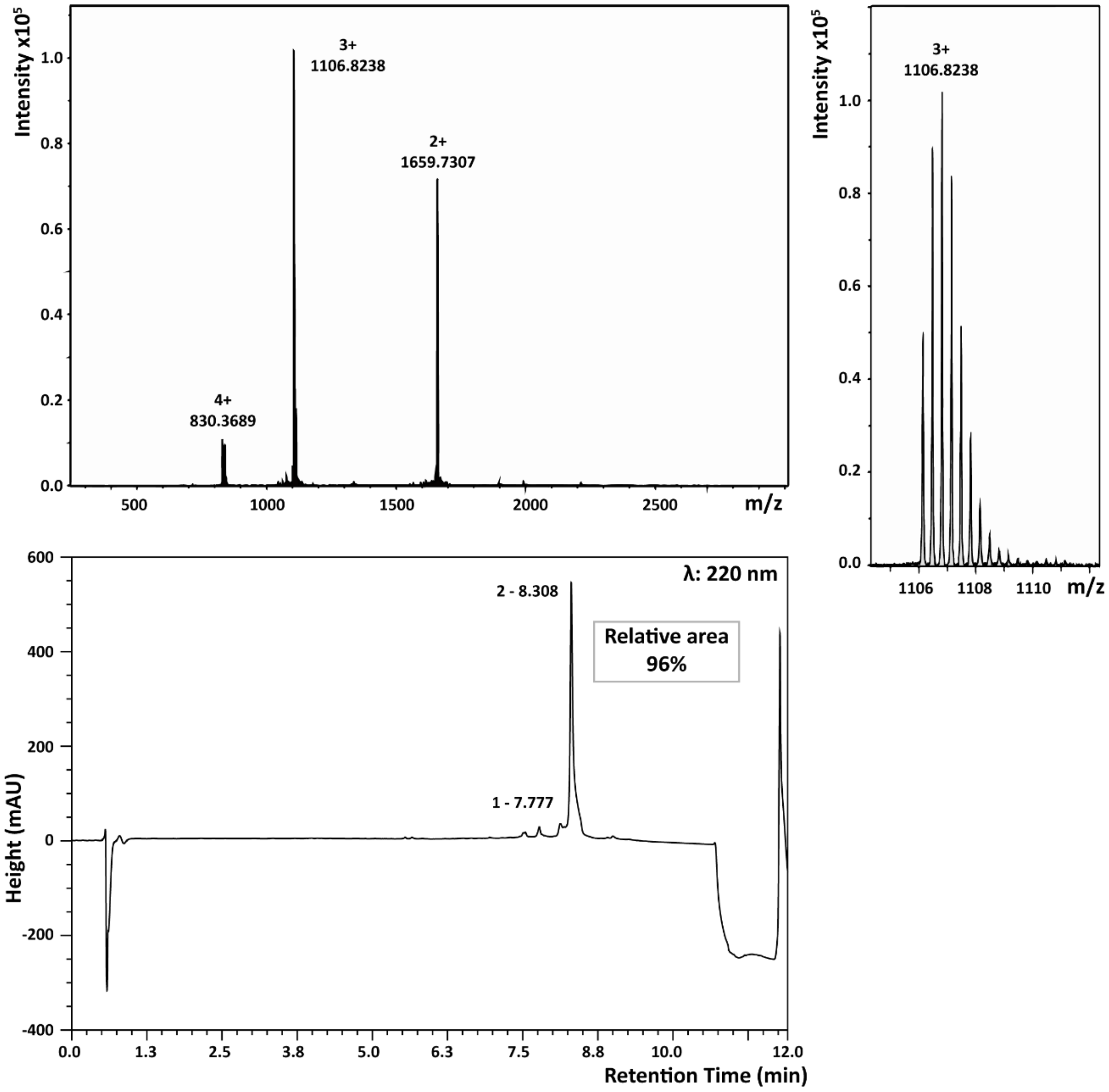

N-Myc $_{61\text{-}89}^{T79C/L83C}$ mal

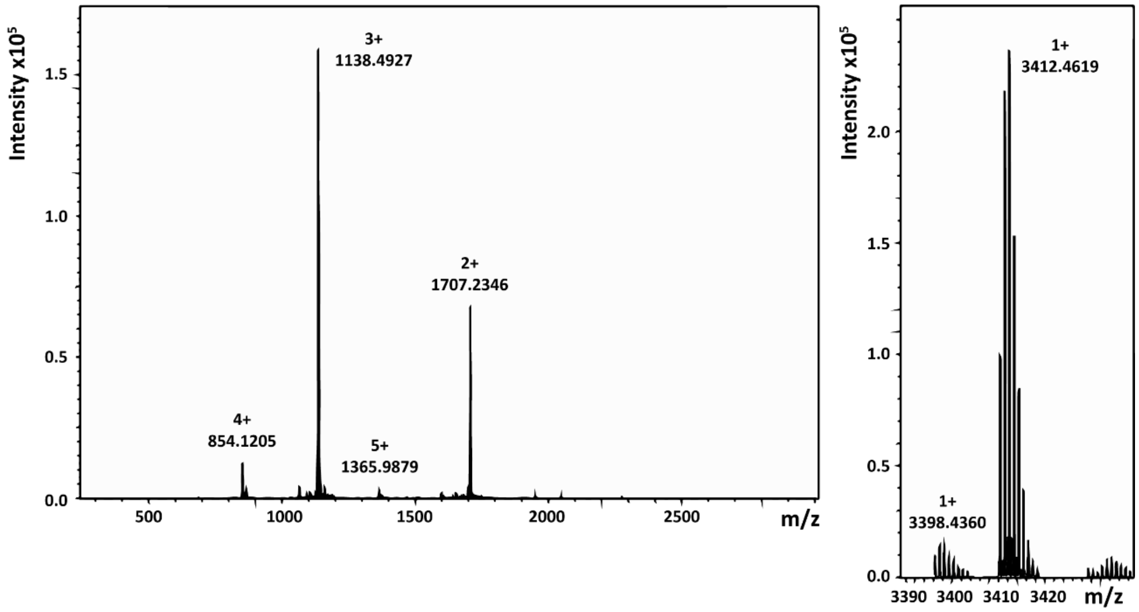

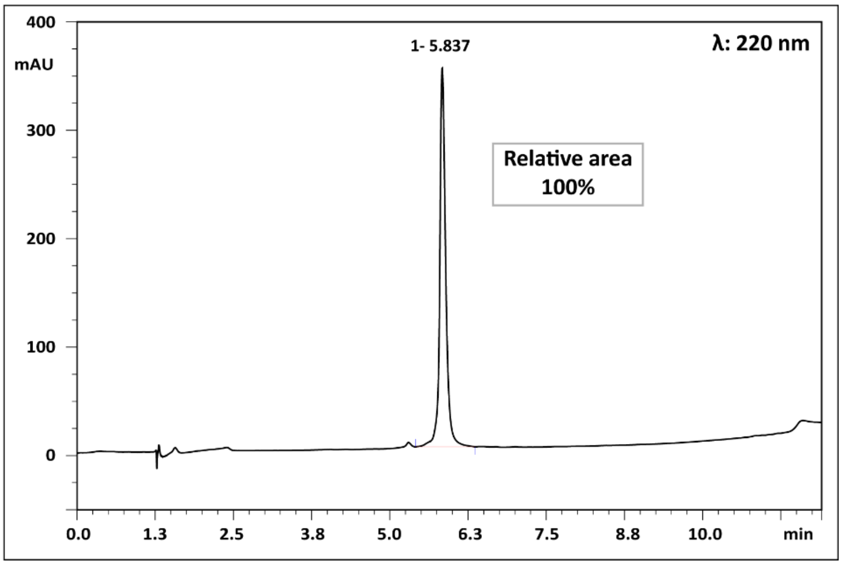

N-Myc $_{61\text{-}89}^{V78C/L82C}$ red

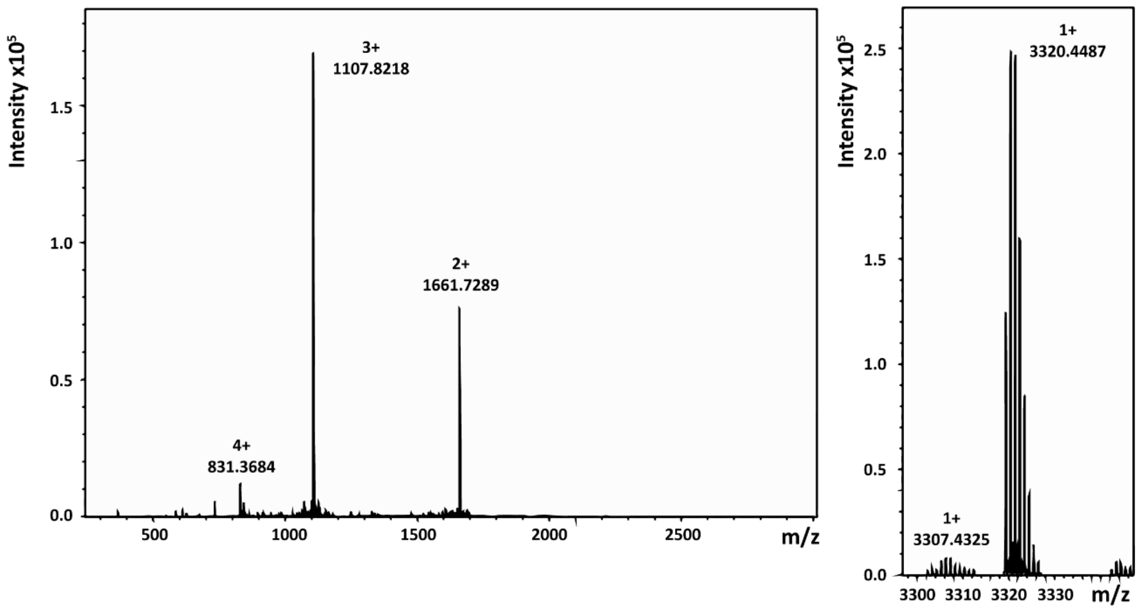

All free-thiol (red) and disulfide bridged (ox) variants originated from the same solid lyophilized sample; analytical HPLC spectra for disulfide bridged (ox) variants are provided.

N-Myc $_{61\text{-}89}^{V78C/L82C}$ ox

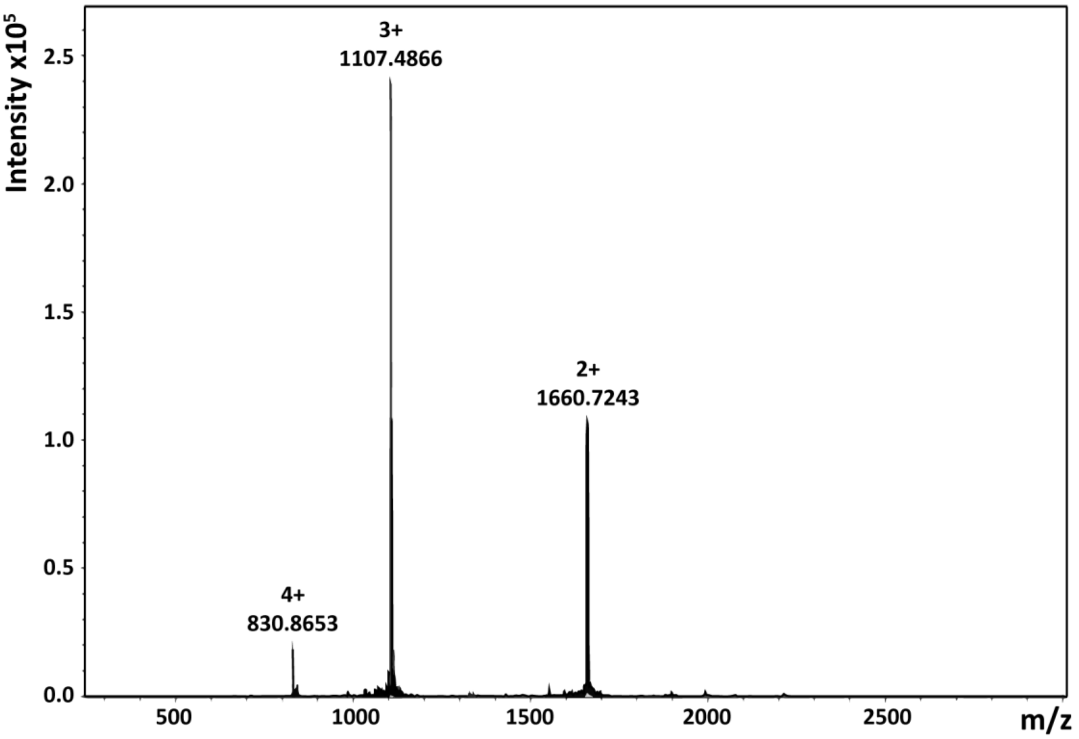

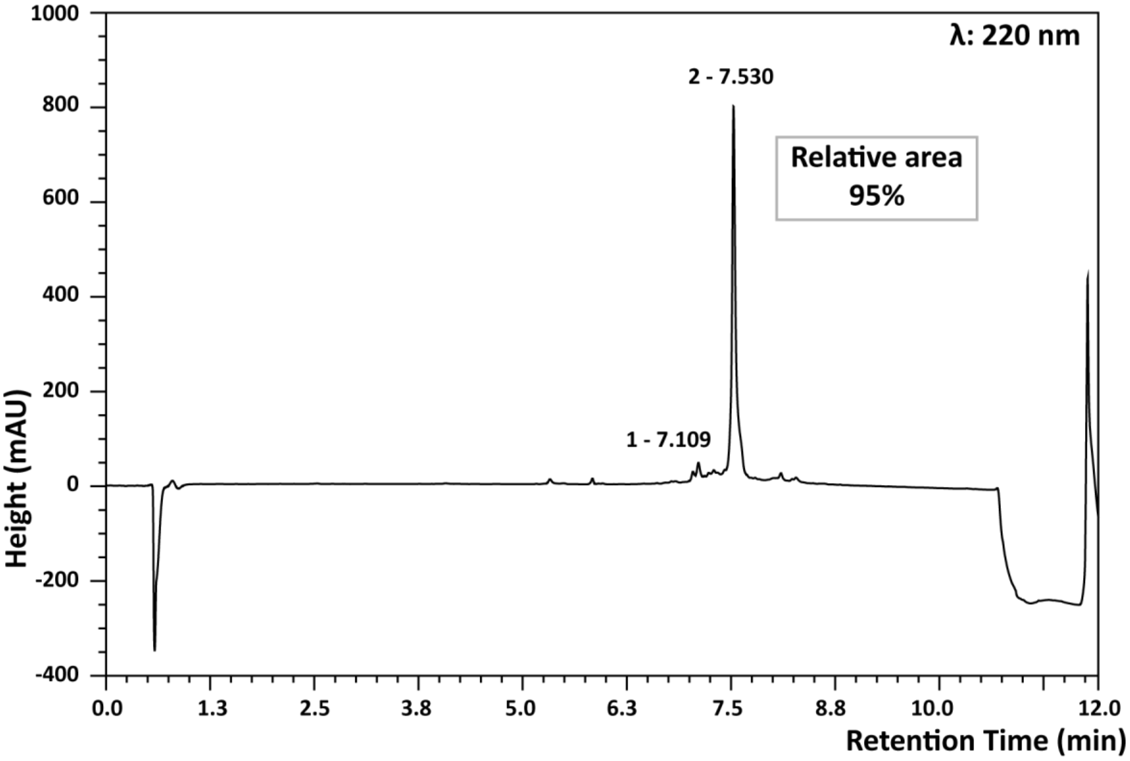

N-Myc $_{61-89}^{V78C/L82C}$ mal

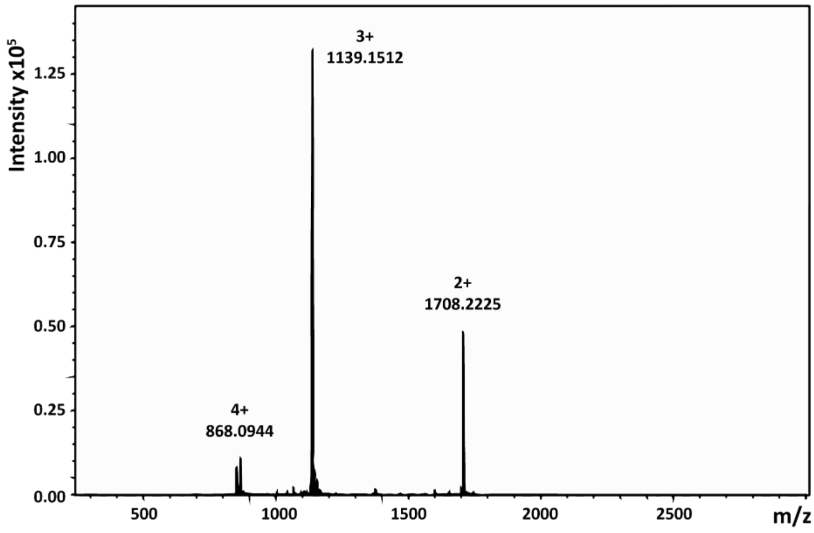

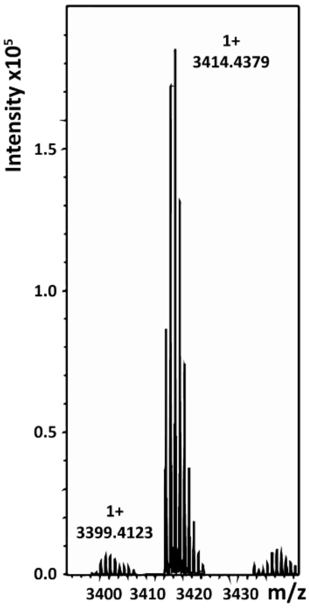

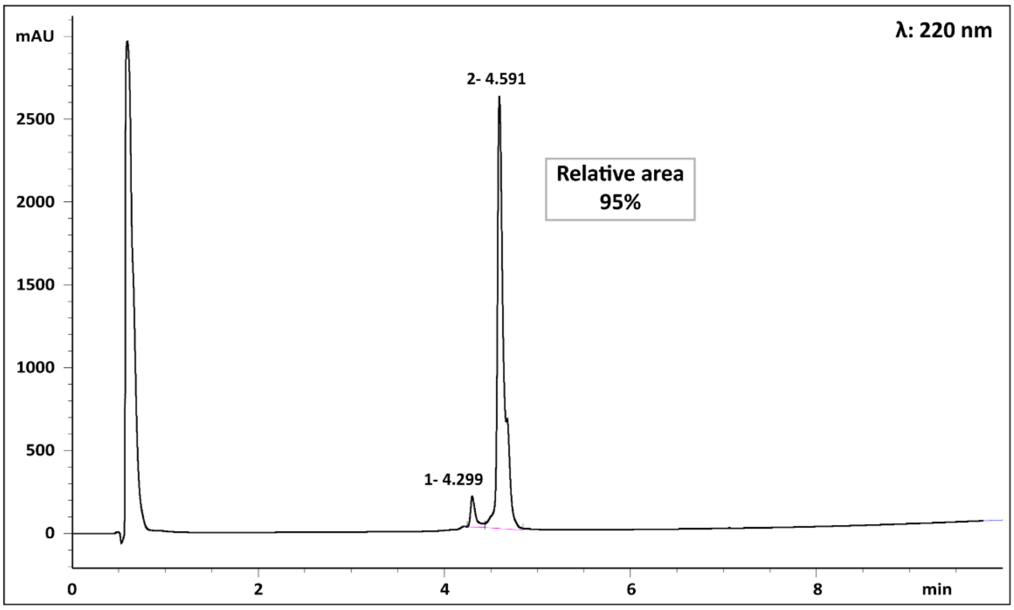

N-Myc $_{61\text{-}89}^{S76C/E80C}$ red

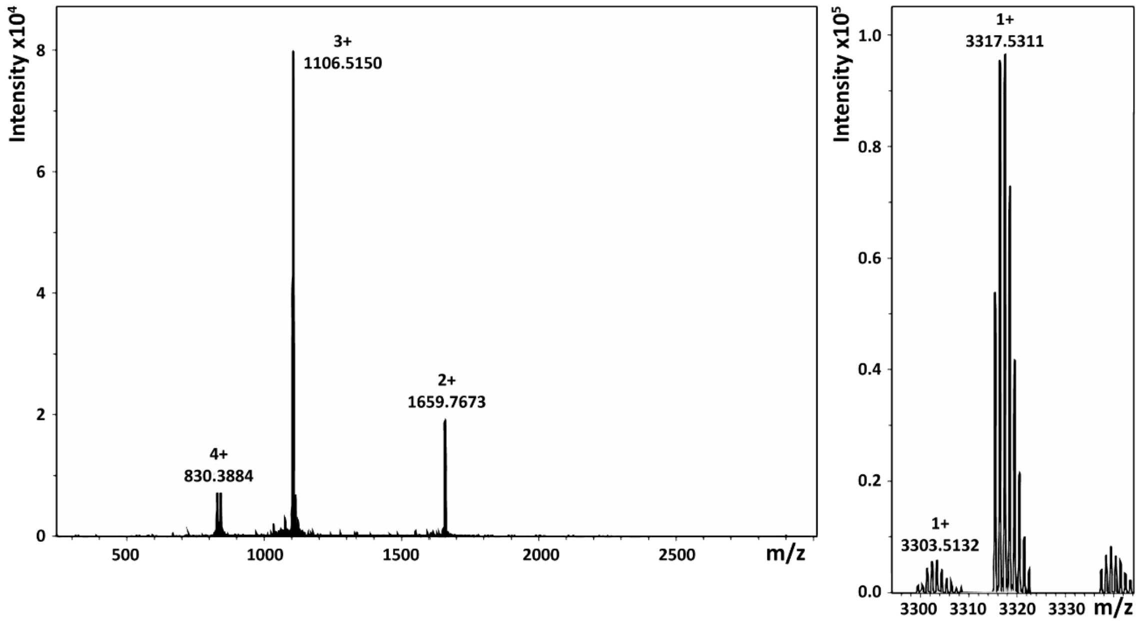

All free-thiol (red) and disulfide bridged (ox) variants originated from the same solid lyophilized sample; analytical HPLC spectra for disulfide bridged (ox) variants are provided.

N-Myc $_{61\text{-}89}^{S76C/E80C}$ ox

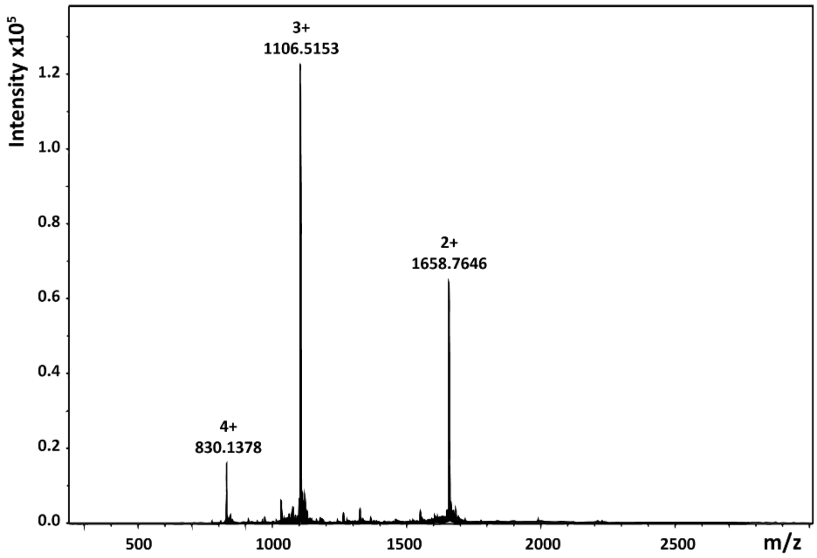

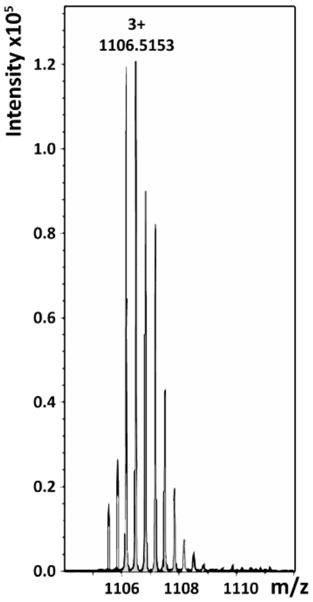

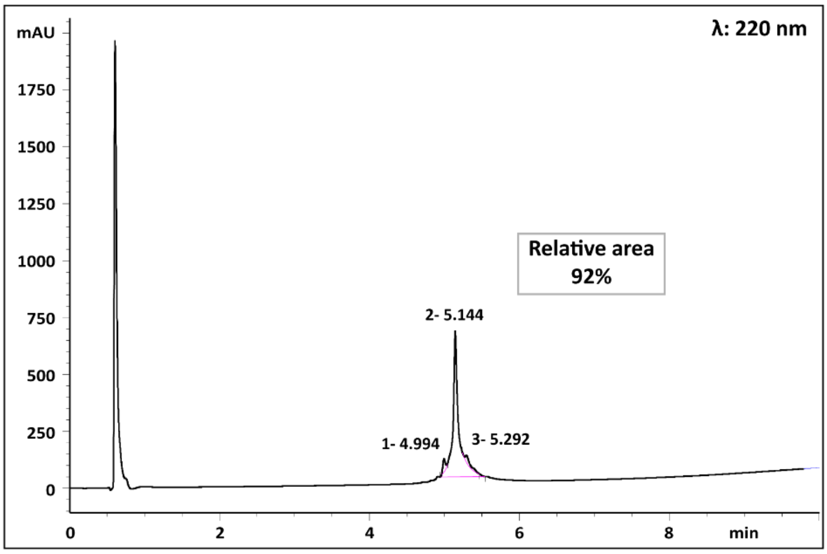

N-Myc $_{61\text{-}89}^{S76C/E80C}$ mal

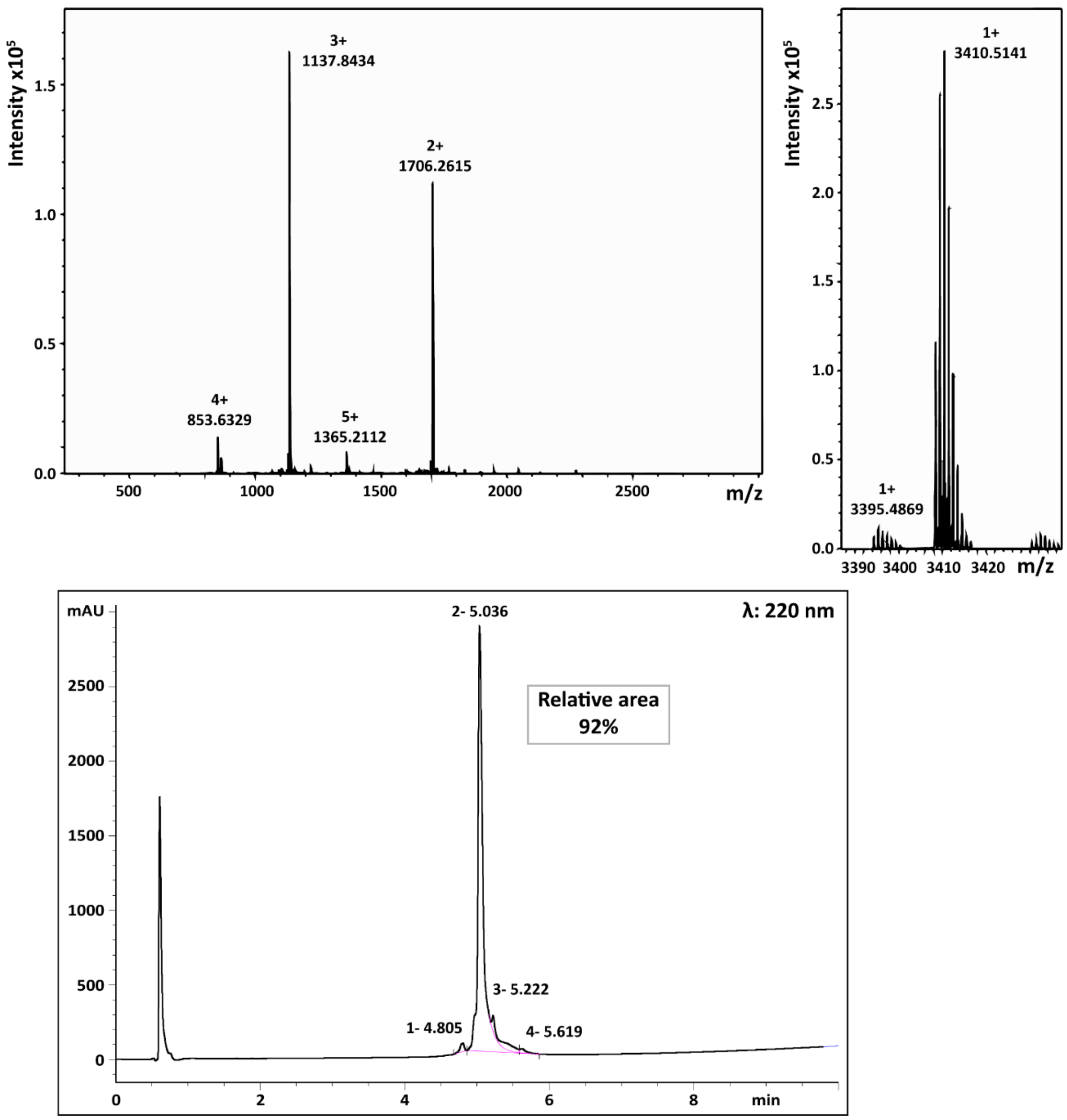

N-Myc $_{73\text{-}94}^{E86C/S90C}$ red

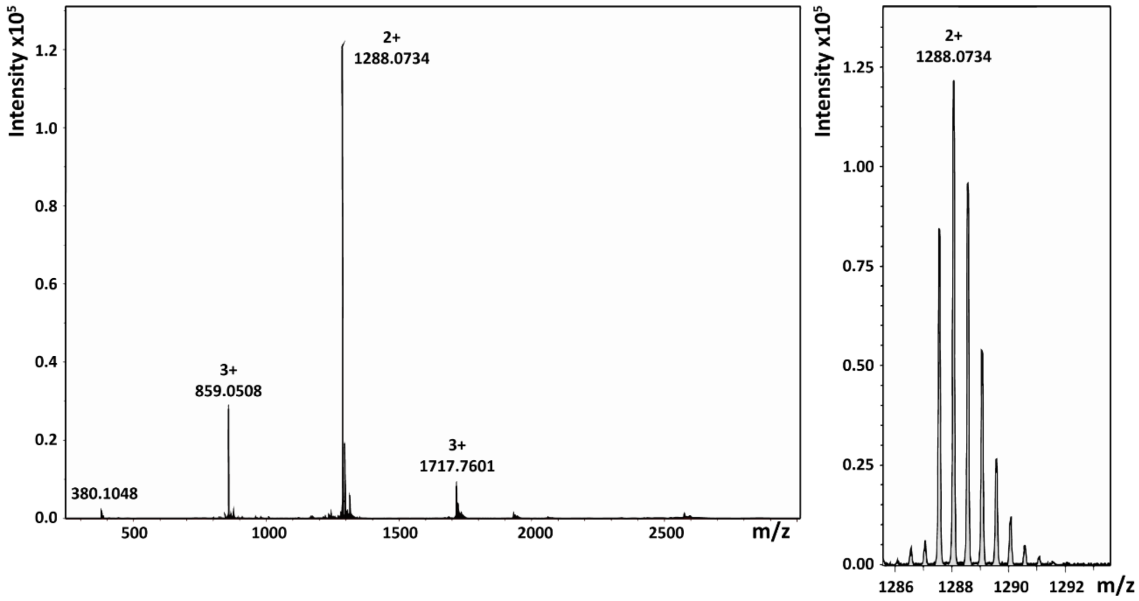

All free-thiol (red) and disulfide bridged (ox) variants originated from the same solid lyophilized sample; analytical HPLC spectra for disulfide bridged (ox) variants are provided.

N-Myc $_{73\text{-}94}{}^{E86C/S90C}$ ox

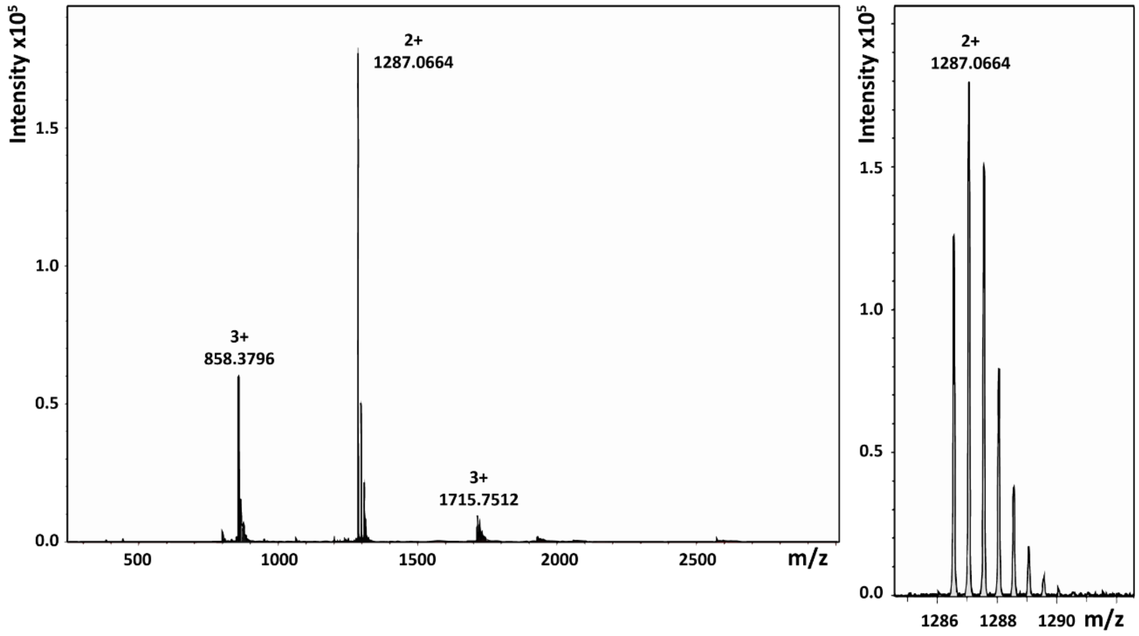

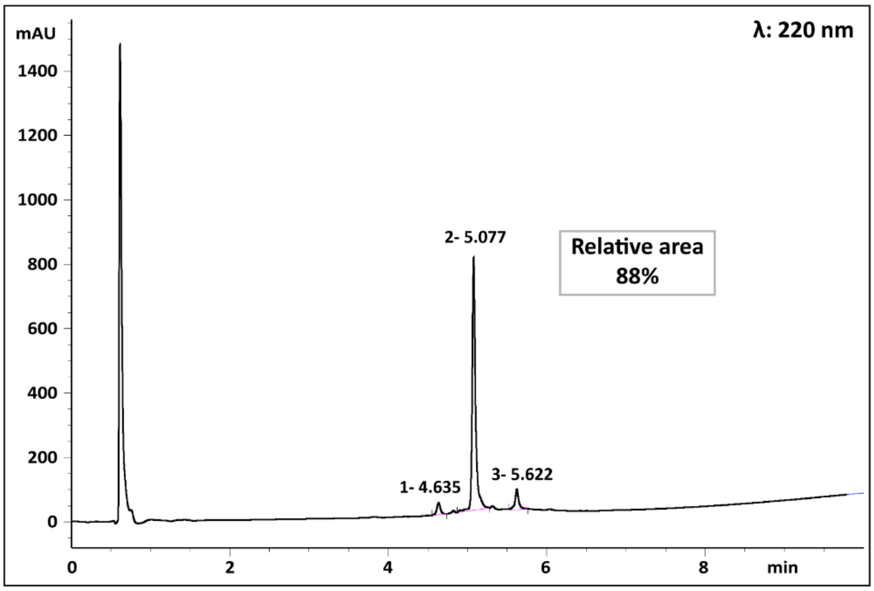

N-Myc $_{73\text{-}94}$ $^{E86C/S90C}$ mal

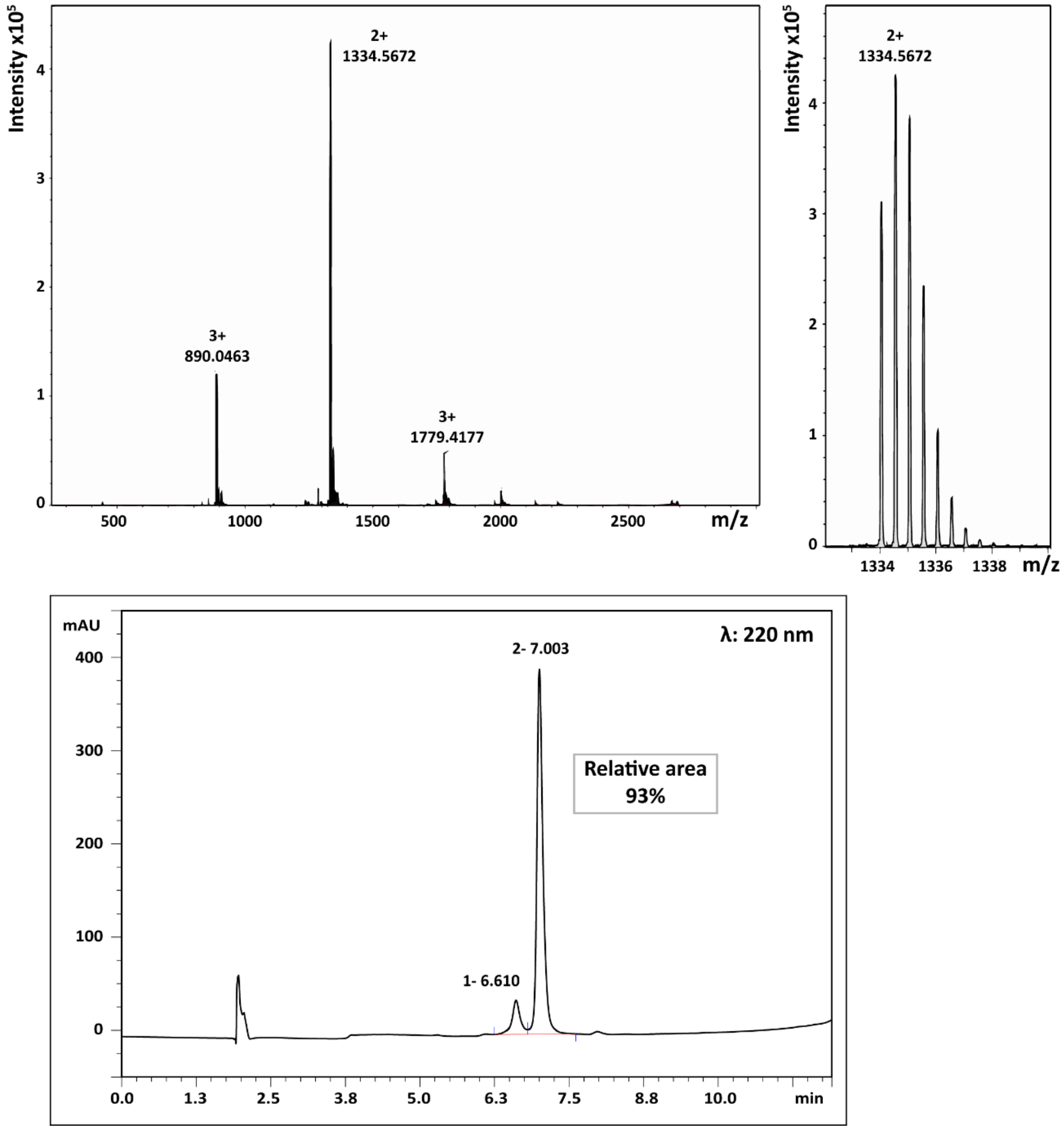

N-Myc $_{73\text{-}94}^{N85C/G89C}$ red

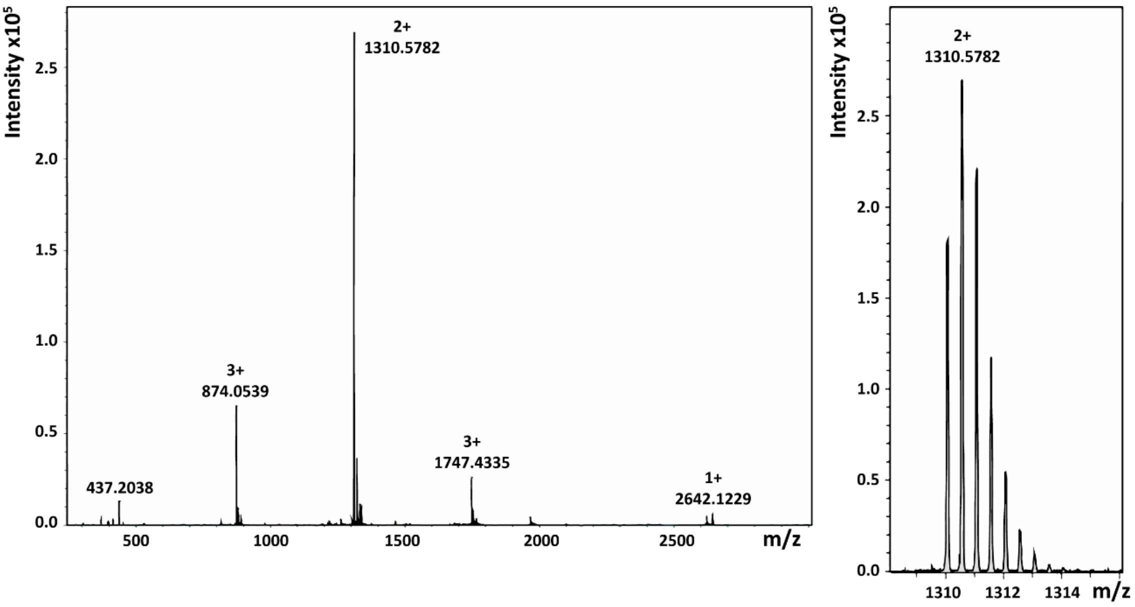

All free-thiol (red) and disulfide bridged (ox) variants originated from the same solid lyophilized sample; analytical HPLC spectra for disulfide bridged (ox) variants are provided.

N-Myc $_{73\text{-}94}^{N85C/G89C}$ ox

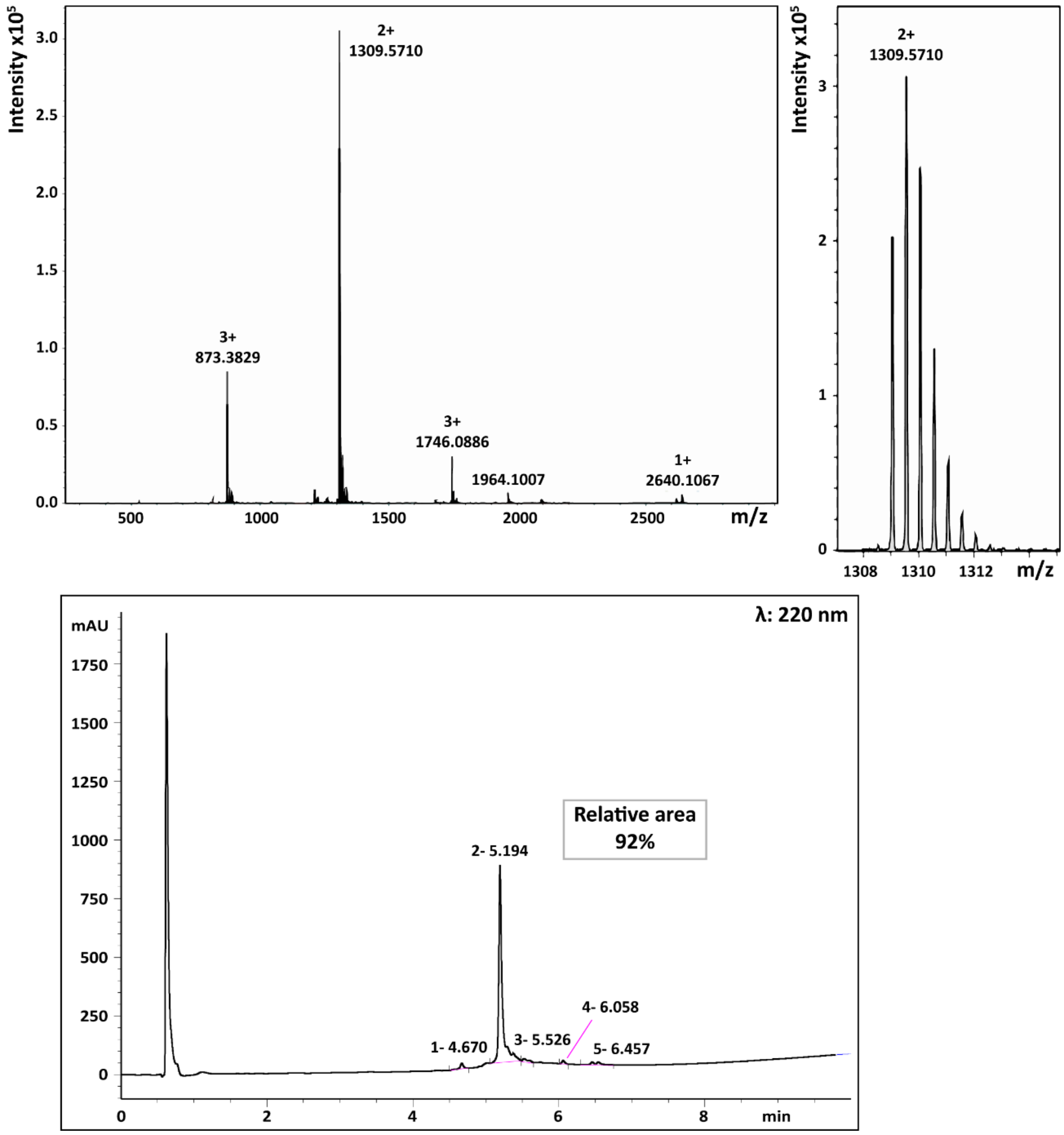

N-Myc $_{73\text{-}94}^{N85C/G89C}$ mal

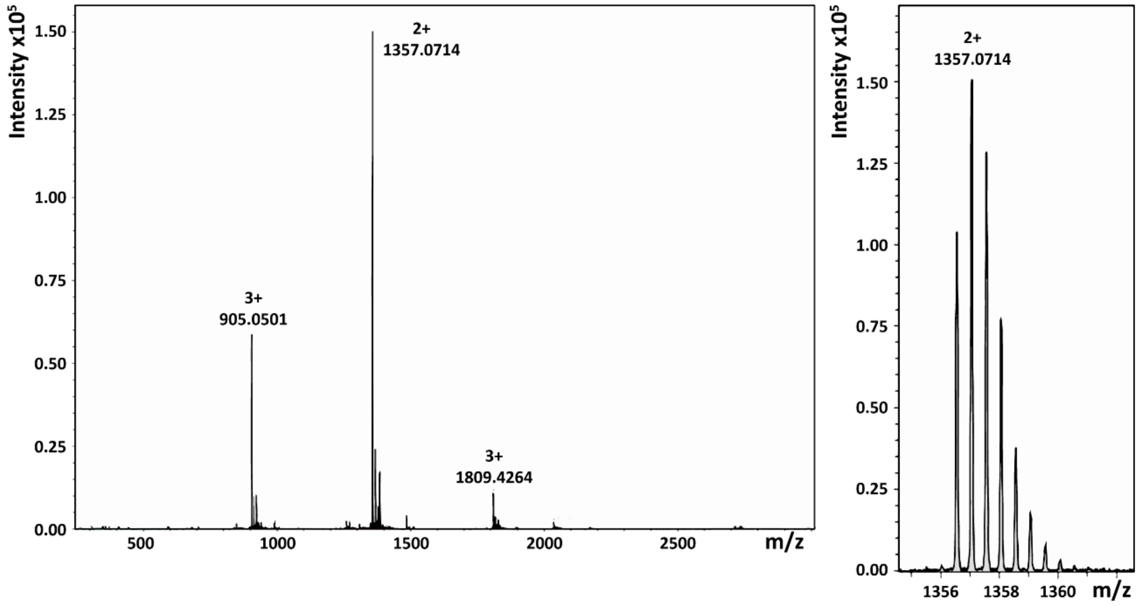

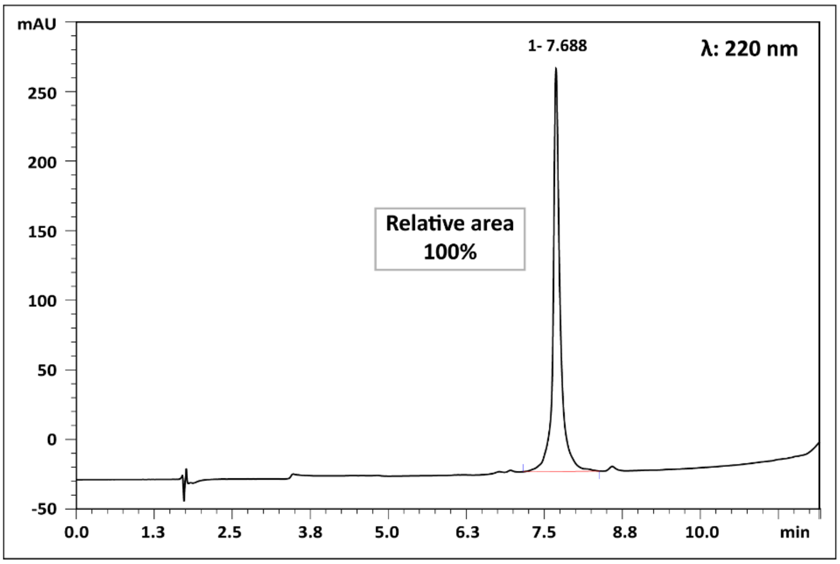

Supplement: Supplementary file 1 — Supporting Information [file CBIC-25-0-s001.pdf]
